# Melamine phosphate-modified magnetic chitosan: a novel biocompatible catalyst for the synthesis of biological tetrahydrodipyrazolopyridine and pyrazolopyranopyrimidine derivatives

**DOI:** 10.3389/fchem.2024.1395008

**Published:** 2024-05-15

**Authors:** Maryam Mousavi-Ebadi, Javad Safaei-Ghomi

**Affiliations:** Department of Organic Chemistry, Faculty of Chemistry, University of Kashan, Kashan, Iran

**Keywords:** melamine phosphate, modified magnetic chitosan, biodegradable catalyst, multicomponent reaction, pyrazole derivatives

## Abstract

A novel biocompatible composite was fabricated by the functionalization of magnetic chitosan with the melamine phosphate (MP) ionic compound to serve as a recoverable and bifunctional catalyst, aiming at the diversity-oriented generation of biological tetrahydropyrazolopyridine and pyrazolopyrimidine derivatives. This involved a meticulously orchestrated reaction, exploiting the in situ generated pyrazole alongside aromatic aldehydes, ammonium acetate, and (thio) barbituric acid. The present work manifests outstanding advantages, offering a novel and great method for the optimal synthesis of two valuable heterocyclic series especially five new derivatives. The resulting novel biocompatible composite was comprehensively characterized through a range of analytical techniques, including FT-IR, NH_3_ and CO_2_-TPD, XRD, TEM, FE-SEM, VSM, EDX, elemental CHNS analysis, ICP-MS, and NMR spectroscopy. Notably, the study represents a critical step in the preparation of advanced materials from accessible and cost-effective precursors.

## Introduction

**SCHEME 1:**
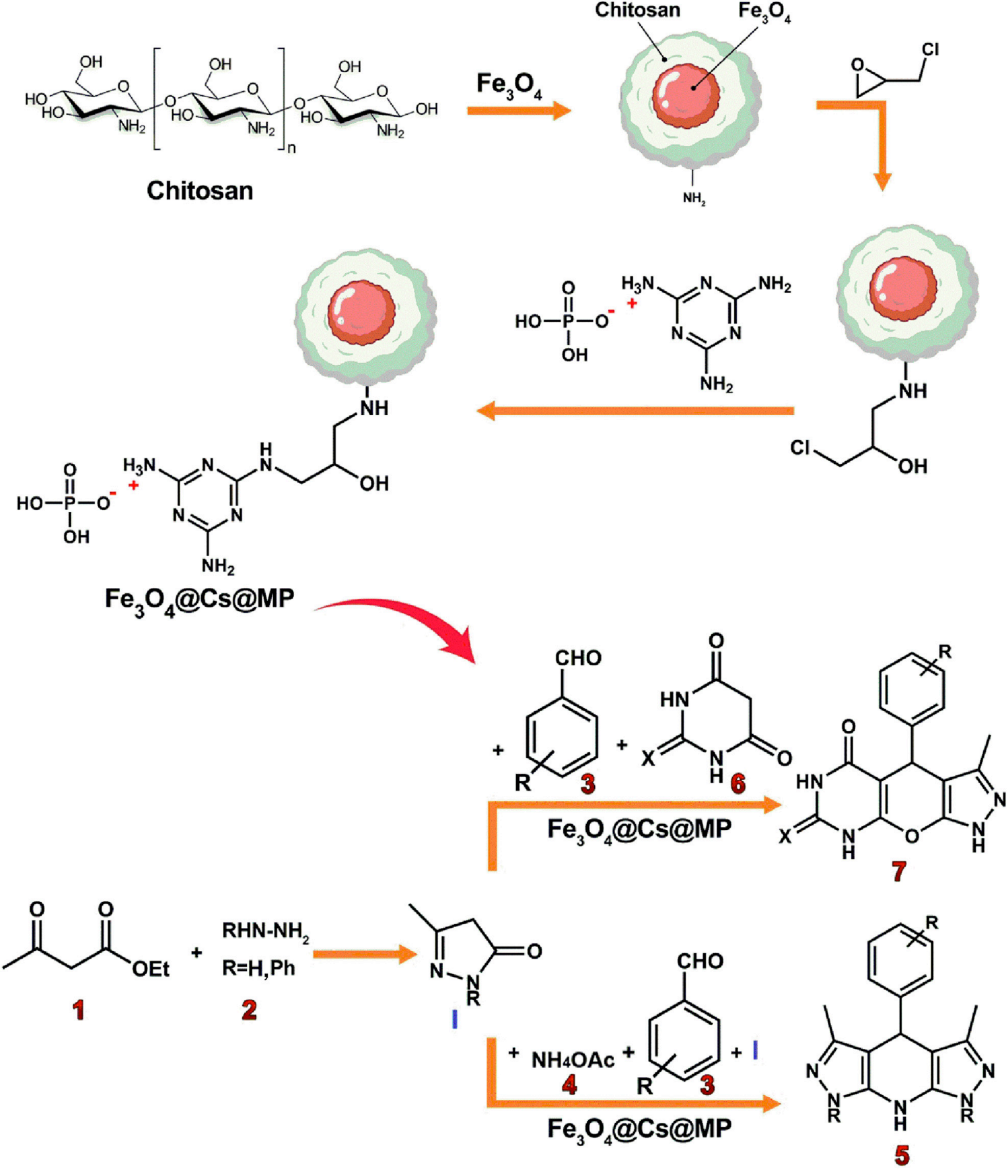
An illustrated summary of the fabrication of the Fe_3_O_4_@Chitosan-Melamine Phosphate for the synthesis of the tetrahydrodipyrazolopyridine and pyrazolopyranopyrimidine scaffolds.

Within diverse arenas, nanoparticles exhibit an expansive capacity, particularly in catalytic systems. Among nanomaterial, metal nanoparticles harness enhanced efficacy within the catalytic process owing to their heterogeneity, high surface area, and customizable morphologies (Cahyana et al., 2021). Notably, magnetite nanoparticles (MNPs) stand out as the most utilizable catalyst solid support in the design of core-shell catalysts. MNPs demonstrate exclusive electrical and magnetic properties (Guo et al., 2012; Ramesh et al., 2019), thus serving as the foundation for the development of novel nanocomposites. Despite the mentioned advantages, challenges such as surface contamination and particle aggregation necessitate particle coating. This coating is implemented in various manners to suit the intended application (Cui et al., 2020; Marandi et al., 2021). Examples of nanoparticle surface modification include the preparation of composites (Safaei-Ghomi et al., 2019; Cahyana et al., 2022), metal-organic frameworks (Sadjadi et al., 2022), and ceramics (Feng et al., 2018). Additionally, biodegradable composites, incorporating a matrix and reinforcement of natural fibers, have gained attention. Polysaccharides such as cellulose, chitosan, and lignin rank among the most widely used natural materials for these structures (Khalilzadeh et al., 2022; Rezaei, 2022). Notably, chitosan a biological, inexpensive, and readily available macromolecule with active functional groups (Beiranvand and Dekamin, 2023) holds promise in enhancing clinical trials and improving physical attributes such as solubility, stability, corrosion resistance, and catalytic activity through surface modification (Mohamed et al., 2014; Özkan et al., 2022). Chitosan surface modification can be achieved by incorporating other active groups through physical loading or covalent grafting (Simkovich and Wagner, 1962; Welton, 1999; Negm et al., 2020). Furthermore, the manufacture of ionic liquids or salts depends on the designed systems. These ionic compounds typically serve as benign and efficient substrates for supporting nanoparticles or modifying porous surfaces (Yun et al., 2010; Safaei-Ghomi et al., 2020). Notably, the capabilities of ionic liquids vary based on the type of anions they contain (Wei et al., 2013; Kumar and Kaur, 2021). In a different domain, melamine, a nitrogen-rich and versatile organic compound, finds application in various chemistry areas such as polymerization (Pedroso et al., 2005), production of Schiff bases (Schwab et al., 2009; Suo et al., 2018), resins (Li and Xue, 2014), covalent organic frameworks (Li et al., 2023), and carbon nitrides (Khan et al., 2019; Ashouri et al., 2023). Melamine exhibits vibrant nucleophilic properties and swiftly transforms into an ionic compound when exposed to active acids (Liu et al., 2017). Its ability to graft onto polymer surfaces through various linkers enhances its efficiency (Wu et al., 2015; Valiey et al., 2019; Bakry et al., 2020). Amidst these advancements, the quest for promoting multicomponent reactions (MCRs) in the field of organic chemistry remains constant (Sakthivel et al., 2023). These reactions, characterized by the coordinated arrangement of functional groups to form the desired structure, offer essential advantages such as one-pot synthesis, high atomic efficiency, and the production of valuable pharmaceutical compounds with excellent efficiency, attracting numerous researchers to this field (Masoumi et al., 2020; Hootifard et al., 2023). Notably, the fusion of hetero polycycles represents one of the applications of multicomponent reactions, allowing for the synthesis of sophisticated structures at one step (Zare et al., 2017, Mohammadi Ziarani et al., 2022). Moving into the realm of heterocyclic chemistry, pyrazoles emerge as critical due to their diverse biological features and active role in drug design (Devi et al., 2018). Pyrazoles serve as the pharmacophore of commercial drugs such as metamizole, aminophenazone, phenylbutazone, and dipyrone (Naim et al., 2016), and act as the building blocks of coenzymes (Tamaddon and Khorram, 2020). Additionally, pyrazole compounds find application in industry as agricultural nutrients and market dyes (Zhang et al., 2016). Notably, fusing the pyrazole ring with other heterocycles gives rise to valuable structures known as biological hybrid compounds, exhibiting diverse properties such as anti-tumor (Singh et al., 2006; Bekhit et al., 2015; Han et al., 2015), anti-bacterial, anti-fungal (Sharma and Singh, 2020; Shaik et al., 2023), anti-inflammatory and anti-viral (Nasr and Gineinah, 2002), as well as properties targeting conditions such as Alzheimer’s, anxiety, depression, and hyperglycemia (Gupta et al., 2023). Pyrazopyridines and pyrazolopyrimidines are recognized as medicinal families within this category (Safaei-Ghomi et al., 2016; Azizi et al., 2019). In synthesizing pyrazole intermediates, the *in situ* via Knorr condensation remains a prevalent method. Notably, enforcing various types of multicomponent reactions for pyrazoles, achieved by arranging the reactants in different one-pot protocols, adds further versatility to their synthesis (Bakherad et al., 2017). In general, the evolving landscape of nanomaterials, surface modifications, and multicomponent reactions presents a dynamic and promising frontier across various scientific disciplines. Considerable efforts have been documented in the pursuit of optimal synthesis strategies for pyrazolopyridines and pyrazolopyrimidines. To this end, diverse catalytic systems have been meticulously developed, including choline chloride/urea (Vanegas et al., 2019), CuFe_2_O_4_@HNTs (Maleki et al., 2019), nano-ovalbumin (Salehi et al., 2014), nano-CuCr_2_O_4_ (Shahbazi-Alavi et al., 2016), Thiamine hydrochloride (Salim et al., 2022), [Tb (W_5_O_18_)_2_]^9-^ (Lotfian et al., 2020), enzyme catalyst (Tamaddon and Arab, 2019), and Fe_3_O_4_/KCC-1/IL/HPW (Ali et al., 2021). Despite the reported efficiency of these catalysts, their utilization is often hampered by high costs, challenges in recovery, excessive precursor usage (such as ammonium acetate), or limitations in producing the desired derivatives. These issues underscore the ongoing efforts to identify the most favorable reaction conditions, aiming to obtain novel substituted analogs of pyrazoles no unwanted side product generation by harnessing nano-materials and natural structures with optimal cost-effectiveness. As far as we know, in the current study, we undertook the pioneering task of modifying magnetic chitosan with a melamine-based ionic compound as unprecedented research. The novel nanocomposite has wide active surface deriving it a powerful catalyst. We probed both Brønsted acid and base sites using NH_3_ and CO_2_-TPD analysis, utilizing them to facilitate six-component condensations for the production of tetrahydropyrazolopyridine. This involved the strategic reaction of a double ratio of ethyl 3-oxobutanoate and a hydrazine derivative, diverse aldehydes, and ammonium acetate. Additionally, these sites were leveraged for the generation of pyrazolopyrimidines through four-component reactions, combining equimolar amounts of ethyl 3-oxobutanoate, hydrazine, aldehydes, and (thio) barbituric acid (Scheme 1). Ultimately, the underlying goal of this investigation is twofold: first, to expand the realm of biocompatible composites, and second, to pursue the purposeful application of catalysts in the synthesis of pharmaceutical structures.

## Experimental

### Chemicals and methods

Medium-molecular-weight Chitosan, thin-layer chromatography (TLC), triethylamine, hydrochloric acid (37%), potassium iodide (KI), sodium hydroxide, zinc oxide, oleic acid, p-toluene sulphonic acid (*p*-TSA), piperidine, potassium hydroxide, acetonitrile, deionized water, dimethyl sulfoxide (DMSO anhydrous, 98%), ethyl 3-oxobutanoate, hydrazine hydrate, phenylhydrazine, benzaldehyde derivatives, (thio) barbituric acid, and (chloromethyl)oxirane were all supplied by Merk company (Germany). Melamine phosphate was prepared by Hefei Jinghui Company, China. Ethyl alcohol (96%), dichloromethane, glacial acetic acid, and acetone (96%) were quality products from drm-chem (Tehran, Iran). The elements in the material were determined by EDX (Kevex, Delta Class I). FE-SEM results were obtained by the LEO device (1455VP), TEM tests were carried out on a Philips EM 208 instrument, and The XRD patterns were conducted on the Philips-X’pert pro diffractometer by a CuKa source (λ = 1.5418 Å). Absorption bands related to the functional groups of the structures were obtained as KBr pellets on the FT-IR spectrometer (Nicolet Magna-550). TGA was measured with a DUPONT 2000 V5.1A device. The study of the magnetic features of the heterogeneous materials was performed by the VSM (Meghnatis Daghigh Kavir Co.; Kashan Kavir; Iran) at ambient temperature.). C%, H%, and N% were measured by the elemental combustion system (Costech 4010). Fe% was measured by ICP-MS analysis through PERKIN-ELMER (ELAN 6100 DRC-e) spectrum. TPD thermograms were registered using the outputs of the PERKIN-ELMER-Spectrum 65 instrument. Mass spectra were recorded on the Agilent Spectrometer, Model; Mass_Spectroscopy 5975C Instrument. ^1^H and ^13^C NMR spectroscopy were provided on a Bruker Advance instrument (400 MHz- Germany) in DMSO-d_6_ solvent. Reaction monitoring was carried out using TLC (silica gel 60 F254). To transfer the organic products from the reaction medium to the next step, were filtered with Whatman 41 filter paper. The melting point of the derivatives of organic products was specified using Electro-Thermal 9200. The melting point of the derivatives of organic products was specified using an Electro-Thermal 9200.

### Preparation of Fe_3_O_4_ nanoparticles

The generation of Fe_3_O_4_ NPs was processed using the typical co-precipitation technique according to the literature (Rahimi et al., 2023). The obtained black precipitate was purified and neutralized with deionized water. Subsequently, the nanoparticles were collected using an external magnet and were vacuum-dried at 50°C.

### *Ex-situ* preparation of magnetic chitosan (Fe3O4@Cs)

Magnetic chitosan was prepared with slight modifications based on a recorded report (Omidi and Mobinikhaledi, 2022). At first, 1 g of chitosan was dissolved in a 2.0 wt% acetic acid solution. Next, the dispersed suspension containing 2 g of Fe_3_O_4_ nanoparticles was added to the solution, and the reaction mixture was dispersed under ultrasonic waves for 1 h in an alkaline environment. Then, it was neutralized and purified with deionized water, and ethanol and subjected to 60°C after separation using an external magnet.

### Linker attachment to magnetic chitosan

The resulting magnetic chitosan (1 g) was suspended in ethanol (40 mL) and the pH was adjusted to 9 with triethylamine. Upon the addition of an epoxychloropropane alcoholic solution, the above mixture was dispersed and refluxed for 7 h at 70°C. Subsequently, it was collected and purified by thorough washing with ethanol. Finally, it was dried at 60°C.

### Preparation of Fe_3_O_4_@Cs-MP

To prepare Fe_3_O_4_@Cs-MP, (1.5 g) of MP was dissolved in DMSO and the medium was alkalized with NaOH (1 mol/L). Magnetic chitosan containing the chlorinated linker (0.5 g) and KI (0.01 g) were added to solution according to Wu^’^s agenda (Wu et al., 2015). The resulting mixture was subjected to ultrasonic waves at 60°C for 2 h. Eventually, the magnetic precipitate was isolated using an external magnet. The resulting solid was washed with DMSO, deionized water and then ethanol to remove unreacted MP and any residual salts. Finally, was dried at 80°C.

### Fe_3_O_4_@Cs-MP-catalyzed synthesis of tetrahydropyrazolopyridine derivatives

Ethyl 3-oxo butanoate (2 mmol), hydrazine (2 mmol), aldehyde derivative (1 mmol), and Fe_3_O_4_@Cs-MP (0.02 g) were taken in a lab tube containing solvents pair of the EtOH-H_2_O (1:1), and the slurry was stirred at 50°C to dissolve precursors. After adding ammonium acetate to the crude, the process development was monitored with the appearance of precipitate and using TLC in ethyl acetate: n-hexane (2:8 ratio) eluent, after the reaction was over. The catalyst was recovered, and the formed precipitate was filtrated after washing with ethanol and dichloromethane, the pure product was obtained and recrystallized in ethanol if necessary. In the end, the product was dried at 80°C.

### Fe_3_O_4_@Cs-MP-catalyzed synthesis of pyrazolopyranopyrimidine derivatives

In a lab tube, hydrazine hydrate (1 mmol), ethyl 3-oxobutanoate (1 mmol), aldehyde derivative (1 mmol), barbituric acid (1 mmol), and Fe_3_O_4_@Cs-MP (0.02 g) were added in ethanol-water solvent pair at 50°C. The reaction development was determined by the formation of the precipitate and by TLC (Eluent: ethyl acetate: n-hexane 2:8). After the process was completed, the magnetic catalyst was pulled out. Finally, the filtered organic products were leached with ethanol and dichloromethane and recrystallized in ethanol if necessary. In the end, product was dried at 80°C.

## Results and discussion

### Characterization of the Fe_3_O_4_@Cs-MP

The efficiency explanation of a catalyst is presented by a survey of its structural chemistry, containing sort of the supports, nature of the active sites, presence of nanomaterials, etc. Each component has a unique effect on the catalytic features of the system, particularly its activity. The discernment of these effects and approval of the desired structure requires the study of surface characteristics, morphology, texture, thermal stability, and other physical characteristics. For this purpose, after preparing the Fe_3_O_4_@Cs-MP catalyst, the structures of the different stages of this process have been checked and confirmed via FT-IR, XRD, EDX, MAP, CHN, ICP-MS, SEM, TEM, NH_3_, CO_2_-TPD, VSM, and TGA.

**FIGURE 1:**
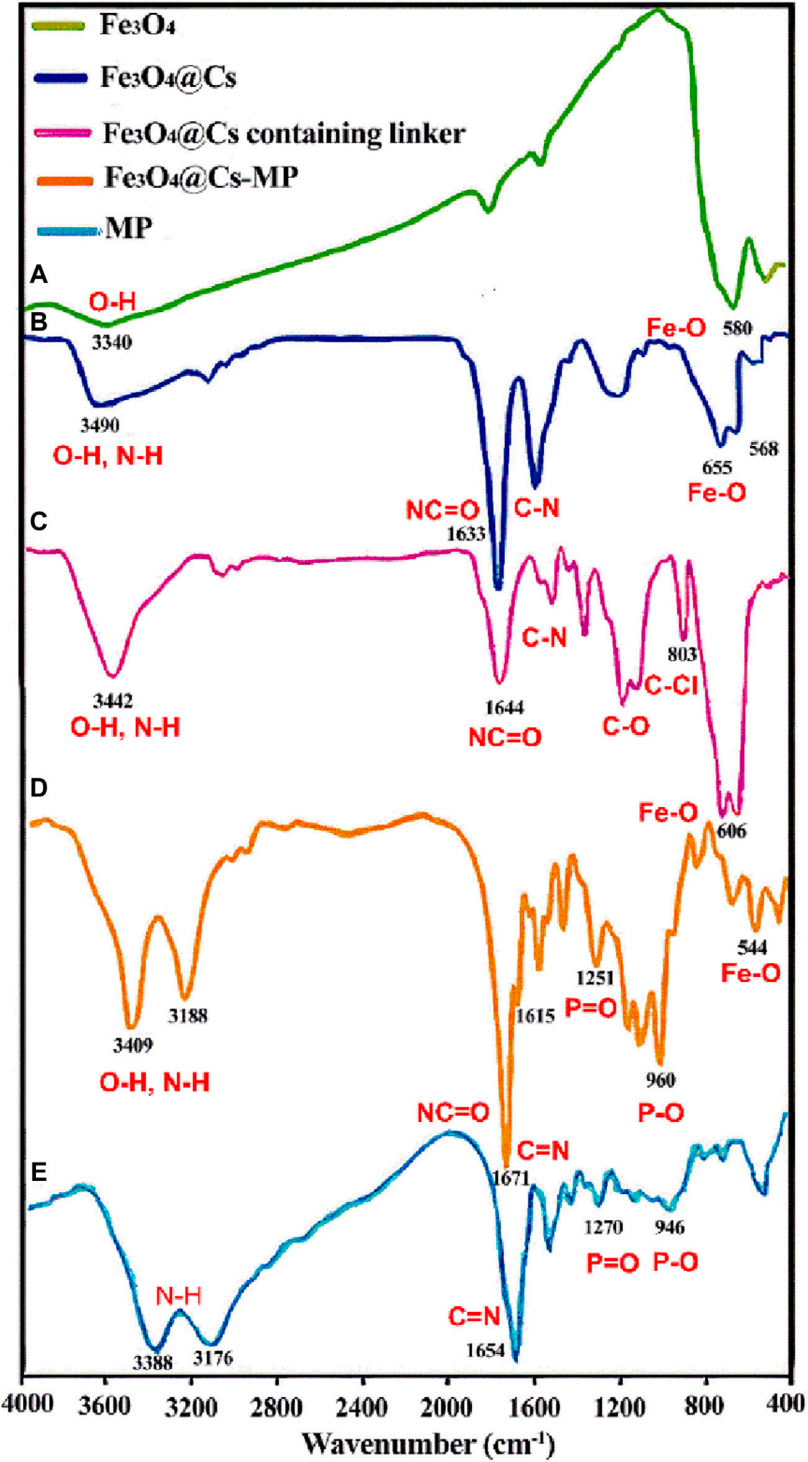
The FT-IR spectroscopy of Iron oxide nanoparticles (Fe_3_O_4_) **(A)**, magnetic chitosan (Fe_3_O_4_@Cs) **(B)**, magnetic chitosan containing linker **(C)**, and melamine phosphate grafted magnetic chitosan (Fe_3_O_4_@Cs-MP) **(D)**, and melamine phosphate (MP) **(E)**.

The confirmation of the final catalyst structure was achieved through a thorough analysis of the main peaks in each step of the FT-IR spectra. In Figure 1, Curve “a” exhibits the absorption bands of the O-Fe-O at 580 cm^−1^ and O–H bonds at 3,340 cm^−1^, aligning closely with existing literature (Sharma and Singh, 2020). Moving to Curve “b”, the presence of peaks for Fe-O in the range of 500–600 cm^−1^, C-H at 2,910 cm^−1^, and O-H at 3,442 cm^−1^ provide compelling evidence confirming the unique structure of magnetic chitosan. Notably, a discernible intensity declines and shift of the O-Fe-O peak compared to free Fe_3_O_4_ signify the interaction of the nanoparticles with active groups within the shell (Su et al., 2021). As we delve into Curve “c”, the emergence of an absorption band related to the C-Cl bond at 803 cm^−1^ solely within this spectrum, followed by its disappearance in the subsequent step, underscores critical changes. Moreover, in Curve “d”, the appearance of strong peaks corresponding to the stretching vibrations of O-H and N-H bonds at 3,409 cm^−1^ and 3,188 cm^−1^, respectively, signifies the augmentation of distinct hydroxyl and amine groups. Notably, the bifurcation of the peak above 3,000 cm^−1^ reflects an increase in different hydroxyl and amine groups. Furthermore, peaks observed in the range of 1,600–1,680 cm^−1^ are attributed to imine bonds within the aromatic ring of melamine and amide groups of chitosan. In addition, the peaks within the range of 1,400–1,600 cm^−1^ are a result of the stretching vibrations of the C-N bond and the bending vibrations of the N-H bond. The distinctive peaks associated with the P-O and P=O groups are localized at 1,251 cm^−1^ and 960 cm^−1^. Notably, these peaks have shifted to higher regions as compared to the peaks arising from intact melamine phosphate functional groups in Curve “e” (Zarandona et al., 2023). As a result, the Fe_3_O_4_@Cs-MP nanocomposite with several functional groups has the potential to activate the precursors within the reactions current.

**FIGURE 2:**
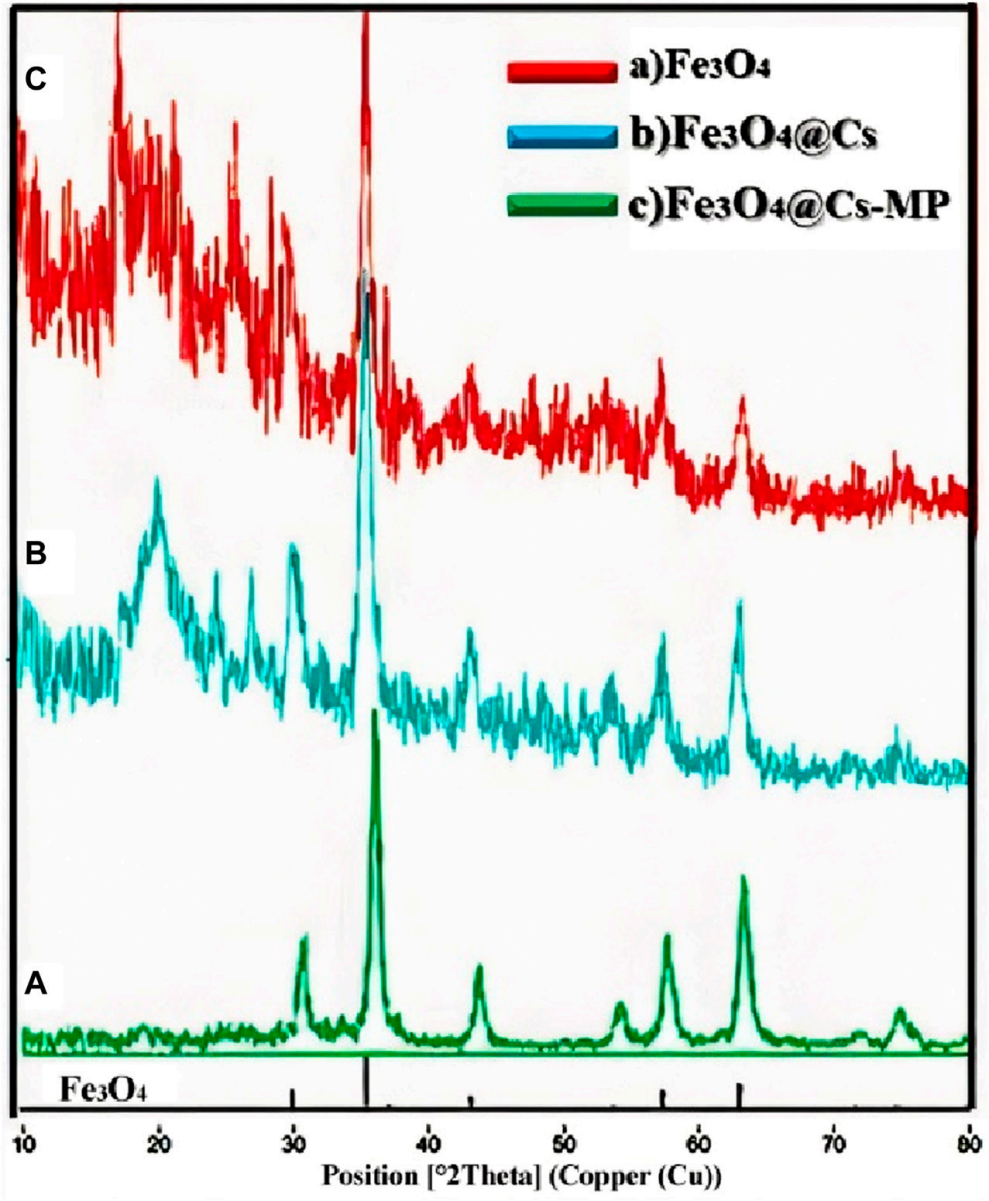
XRD pattern of Fe_3_O_4_ **(A)**, Fe_3_O_4_@Chitosan **(B)**, and Fe_3_O_4_@Chitosans-Melamine Phosphate **(C)**.

Upon conducting the XRD analysis, the clear alignment of the nanoparticles’ peaks with the provided peak list becomes readily apparent. As illustrated in Figure 2, the defining peaks of Fe_3_O_4_ prominently manifest across all stages of the pattern. Moreover, the distinct peak characteristic of chitosan emerges at 20°. Notably, upon the grafting with MP, the emergence of intense, new structure peaks within the range of 15°–35° becomes strikingly evident.

**FIGURE 3:**
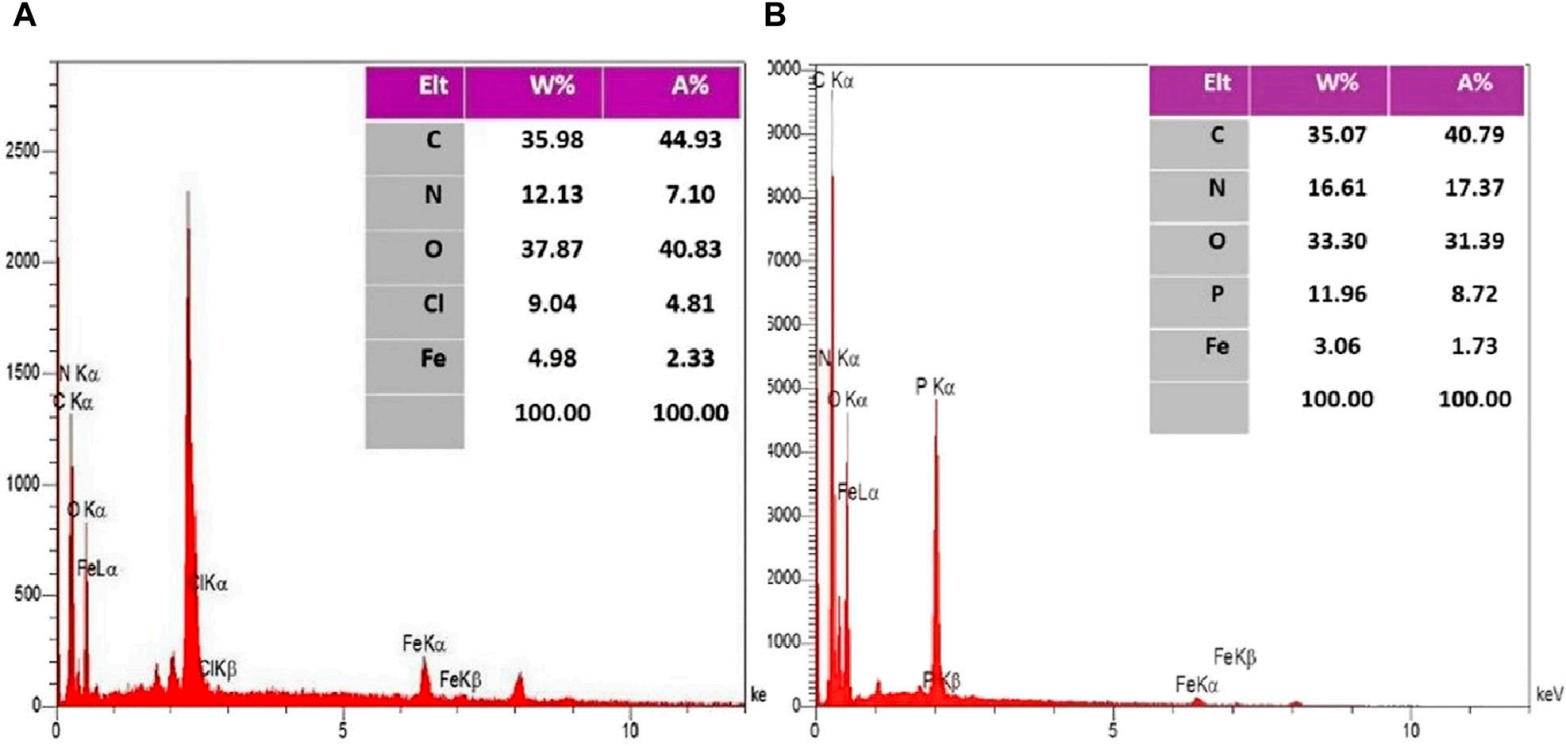
EDX analysis of Fe_3_O_4_@Cs-R-Cl **(A)** and Fe_3_O_4_@Cs-MP **(B)**.

**FIGURE 4:**
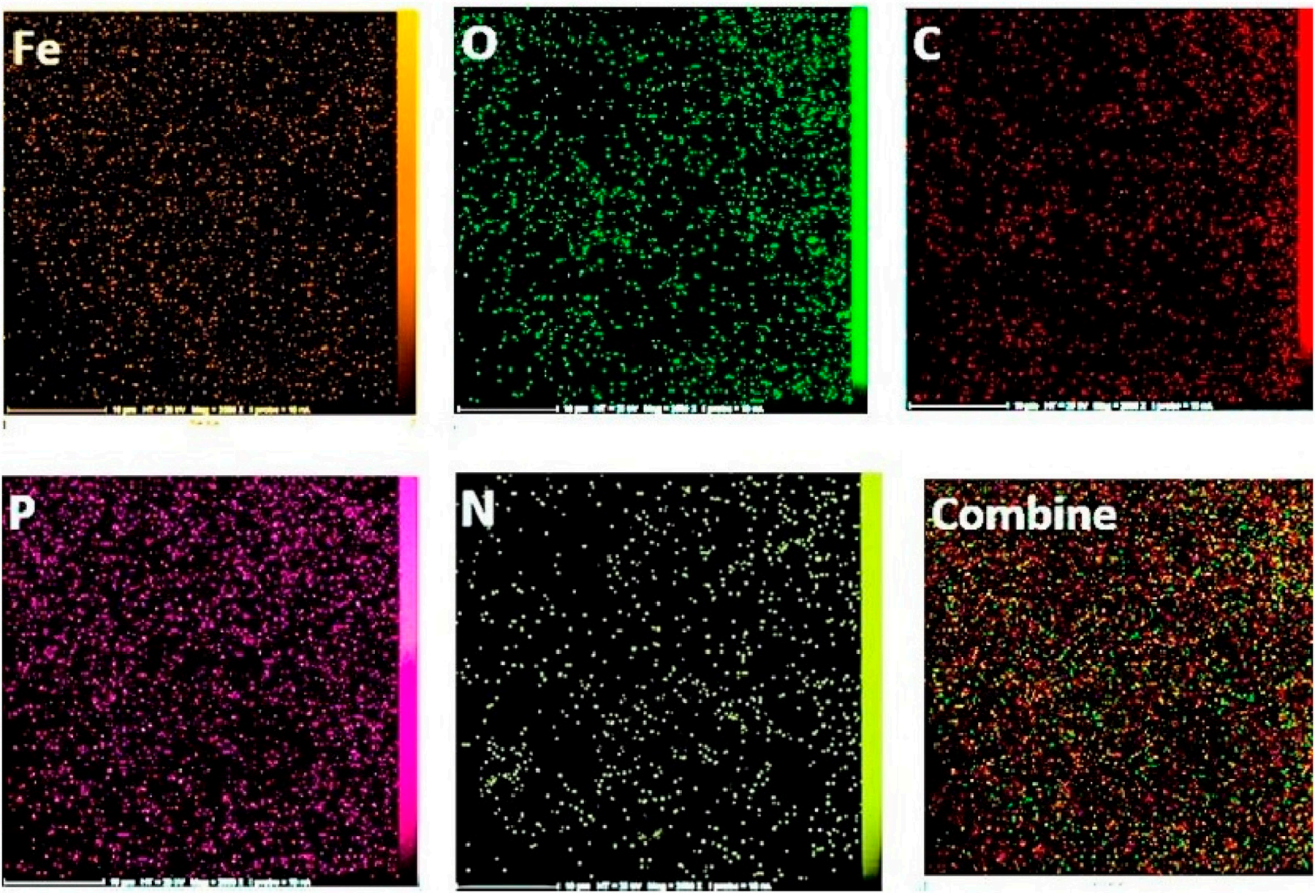
Mapping analysis of Fe_3_O_4_@Cs-MP.

The EDX spectroscopy associated with the final two stages illustrates the elemental composition percentages in each step, as depicted in Figure 3 and elemental mapping results in Figure 4 showed that all elements are satisfactory distributed in across the Fe_3_O_4_@Cs-MP slices. Notably, the appearance of chlorine peaks in the penultimate step serves to validate the linker connection. Furthermore, the observation of peaks representing N, C, Fe, O, and P elements, alongside the absence of undesired elements in the final stage, serves to unequivocally confirm the formation of the intended structure. Also, the results of EDX and mapping of Fe_3_O_4_ nanoparticles and Fe_3_O_4_@Cs are shown in Supplementary Material (Figures 1–3), confirming the expected structures. Furthermore, ICP MS analysis detected the presence of 10.8% iron in the Fe_3_O_4_@Cs-MP.

According to Table 1, the elemental analysis (CHNS) was applied to calculate the C%, H%, N%, and S% in sample. This analysis served as a method to assess the loading of ionic compounds onto the Fe_3_O_4_@Cs@linker surface. The results demonstrated a significant increase in the nitrogen weight percentage, confirming the grafting of the nitrogen-rich structure of melamine.

**TABLE 1:** CHNS analysis of the different stages of chitosan modification.

Sample	C%	H%	N%	S%
Fe_3_O_4_@Cs	22.51	3.96	5.21	0
Fe_3_O_4_@Cs-MP	31.96	4.29	10.97	0

**FIGURE 5:**
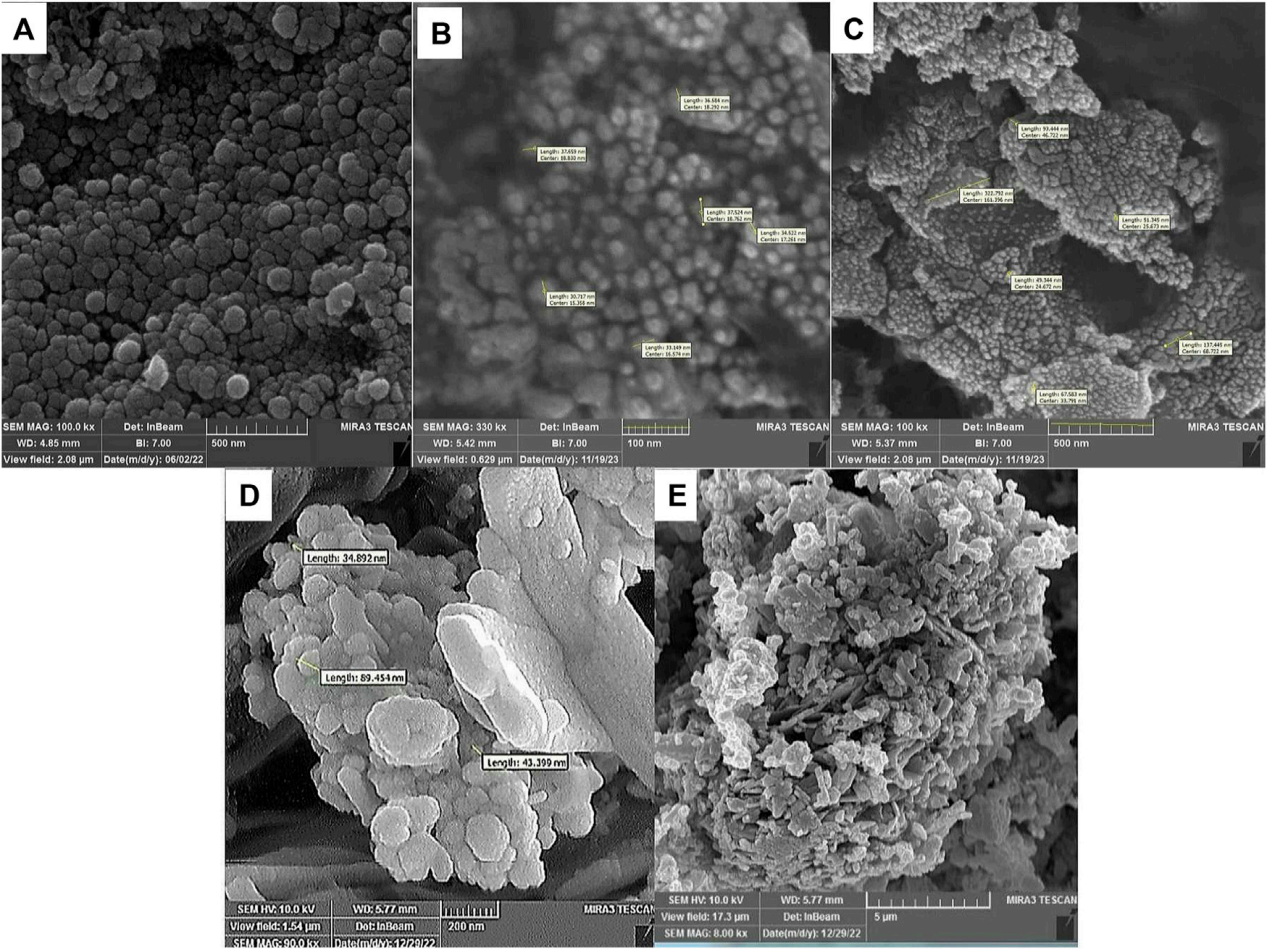
FE-SEM analysis of Fe_3_O_4_ **(A)**, Fe_3_O_4_@Cs **(B,C)**, Fe_3_O_4_@Cs-MP **(D,E)**.

SEM and TEM techniques are used to determine the characteristics of the surface, morphology, and texture of the composite (Daraie et al., 2021; Xue et al., 2021; Karimi-Nami et al., 2023). Figure 5 presents the FE-SEM results at each stage of catalyst fabrication. The SEM micrograph of the magnetic nanoparticles reveals a semi-spherical shape for bare Fe_3_O_4_. Upon examining the FE-SEM image of the Fe_3_O_4_@Cs nano-catalyst, it becomes evident that the nano-catalysts are uniformly affixed to the chitosan surface and has homomorphic texture. Notably, the uneven and porous surface observed in Fe_3_O_4_@Cs-MP, coupled with the discernible increase in nanoparticle size, serves to confirm the attachment of MP to the magnetic matrix. This active surface is one of the virtues of the prepared catalyst.

**FIGURE 6:**
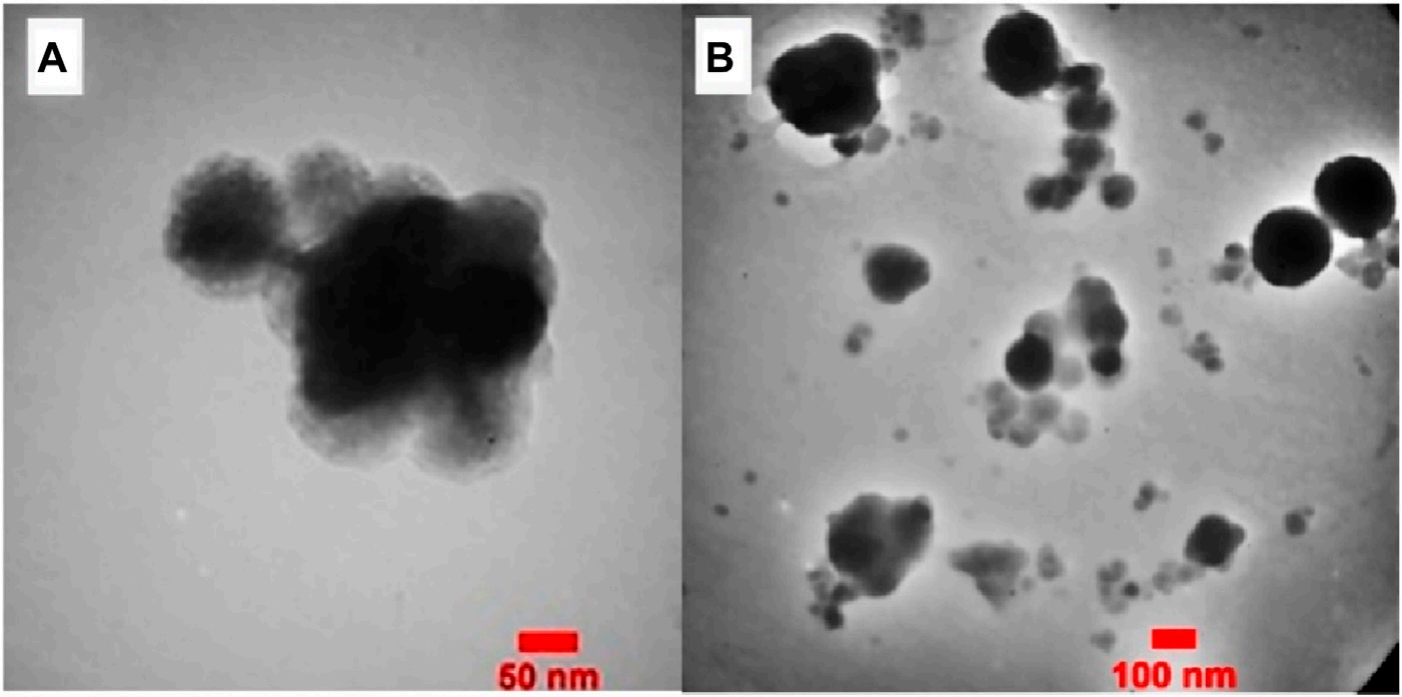
**(A,B)** TEM analysis of Fe_3_O_4_@Cs-MP in different magnifications.

In Figure 6. The TEM images of Fe_3_O_4_@Cs-MP showed that the morphology of the catalyst particles is spherical and has a core-shell structure. The dark micelles represent nonmagnetic particles entrapped in bright areas, indicating the modified chitosan network.

**FIGURE 7:**
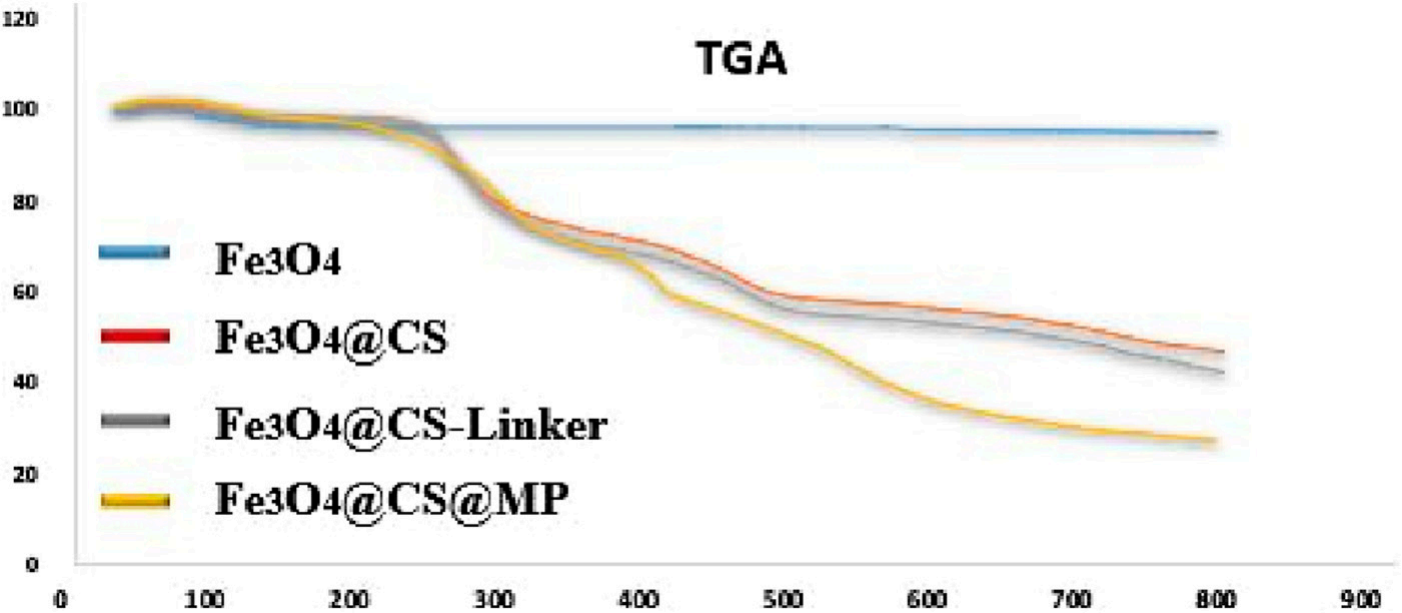
Thermal gravimetric analysis (TGA) Fe_3_O_4_@Cs-MP.

The thermal behavior throughout the preparation stages of the Fe_3_O_4_@Cs-MP catalyst up to 800°C was meticulously scrutinized through TGA analysis (Figure 7). In Curve “a”, following calcination, the combustion of magnetic nanoparticles between 250°C and 800°C resulted in a 4.99% weight loss within the sample. As we delve into the TGA curve for magnetic chitosan (Curve “b”), a substantial 53.18% weight loss unfolds, characterized by three distinct descending slopes representing moisture removal (3.7%), chitosan membrane dissociation (40.28%), and ultimately, nanoparticle degradation (9.2%). Moving to Curve “c”, a further 4.73% weight loss is observed. Notably, curve “d", representing Fe_3_O_4_@Cs-MP, unveils several breakpoints within the final structure, corresponding to the presence of MP grafted onto the magnetic chitosan. In total, compared to the previous stage, there has been a 15.8% decrease. The initial 2.3% weight loss evident before 200°C can be attributed to the volatilization of solvents and water. With the temperature rise, the decomposition of the organic shell commences from the outermost layer, and a noteworthy 27% of the remaining weight at 800°C is linked to Fe_3_O_4_. In addition to confirming the structure, these observations show acceptable thermal stability of the Fe_3_O_4_@Cs-MP catalyst.

**FIGURE 8:**
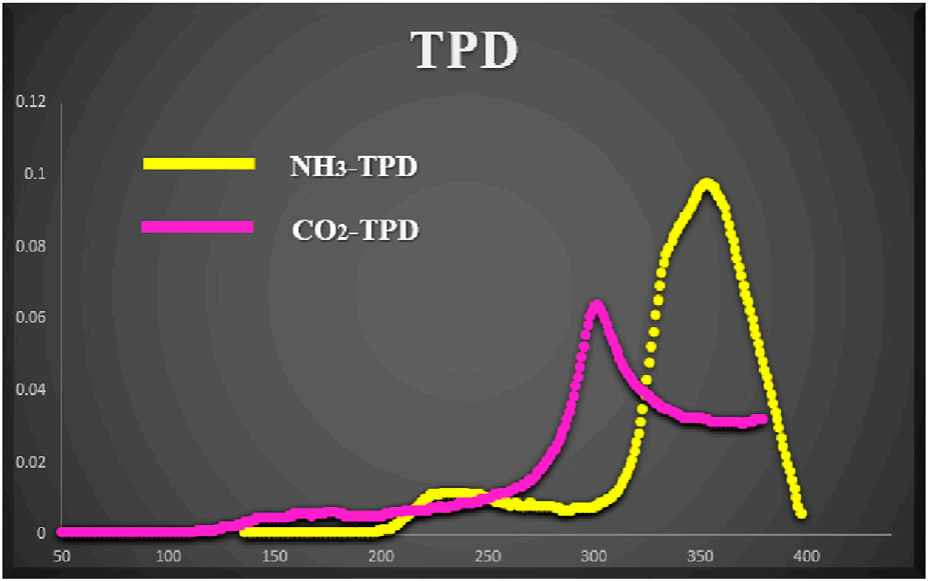
NH_3_, CO_2_-Temperature-programmed desorption profiles of Fe_3_O_4_@Cs-MP.

To ascertain both the acidic and basic roles of the catalyst after melamine phosphate binding, TPD profiles (as depicted in Figure 8) were examined in the 40°C–400°C range. The initial tests were conducted following the method outlined in the literature (Ali et al., 2021). An absorption peak was observed within the moderate range of acidity and basicity. Based on the absorption behavior, it was noted that the NH_3_ absorption ceased at approximately 400°C owning to the thermal decomposition of melamine phosphate and the loss of acidic sites.

**FIGURE 9:**
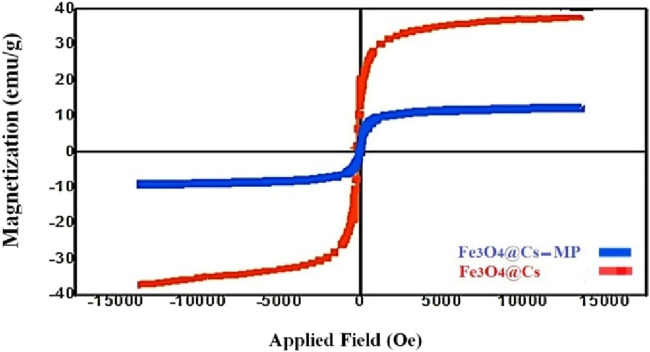
VSM results of Fe_3_O_4_@Cs-MP and Fe_3_O_4_@Cs.

Delving into the magnetic properties, the magnetic features of the Fe_3_O_4_@Cs-MP catalyst and Fe_3_O_4_@Cs were examined using VSM analysis at 25°C, as illustrated in Figure 9. This inquiry reveals a reduced magnetic property of the produced magnetic biocomposite compared to magnetic chitosan, attributable to the increased organic coating on the nanoparticles’ surface. Nonetheless, the VSM results affirm the paramagnetic behavior and acceptable magnetic separability.

### Investigating the catalytic behavior of Fe_3_O_4_@Cs-MP nanocomposite

At the outset, the focus was on obtaining the optimal reaction conditions. Six-component reactions, comprising a double ratio of hydrazine derivative and ethyl 3-oxobutanoate to 4-chlorobenzaldehyde and ammonium acetate, were chosen, as well as a four-component process including ethyl 3-oxobutanoate, hydrazine, barbituric acid, and 4-chlorobenzaldehyde, as model reactions. Various parameters such as temperature, catalysts, and solvents were investigated in both multicomponent reactions aimed at synthesizing pyrazole derivatives (refer to Tables 2, 3).

**TABLE 2:** Optimization of reaction condition to prepare derivative **5a**.

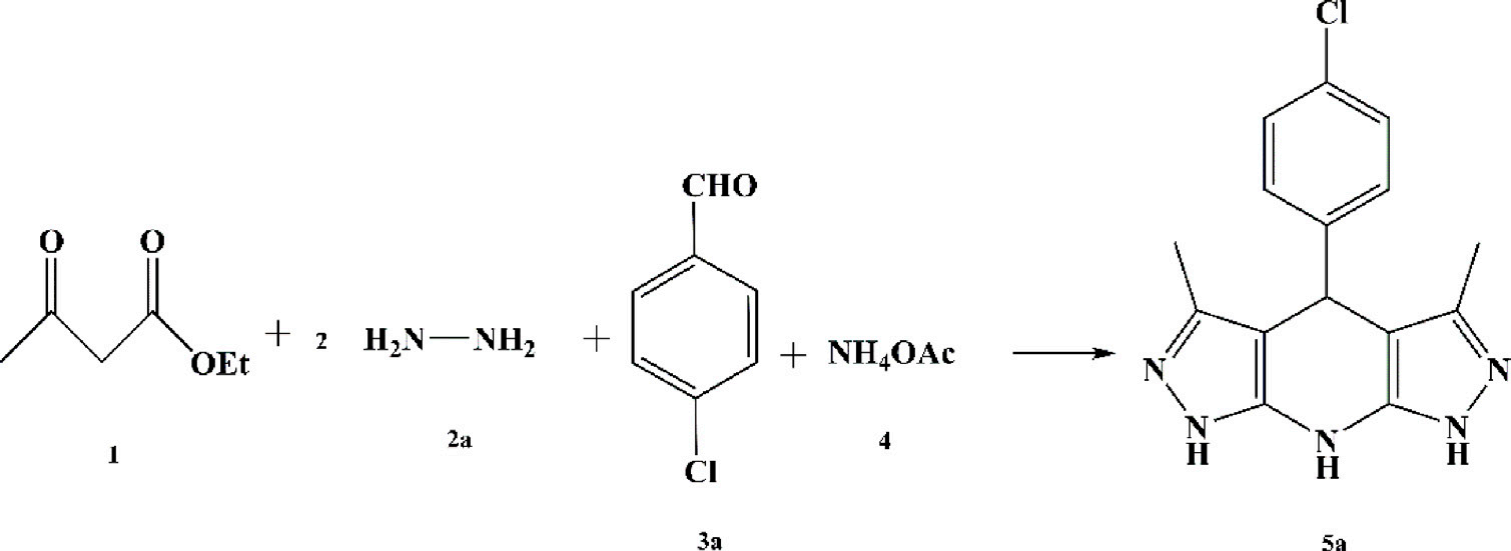				
Entry	Catalyst (g)	Condition	Time (min)	Yield (%)
1	-	EtOH, r.t.	360	-
2	-	H_2_O, r.t.	360	20
3	NEt_3_ (0.05)	EtOH, r.t.	360	-
4	ZnO (0.05)	EtOH, r.t.	360	42
5	p-TSA (0.05)	EtOH, 80°C	360	60
6	K_2_CO_3_ (0.05)	EtOH, 80°C	360	36
7	Piperidine (0.05)	EtOH, 80°C	360	68
8	Fe_3_O_4_ (0.05)	EtOH/H_2_O, 80°C	360	38
**9**	Fe_3_O_4_@Cs (0.05)	EtOH/H_2_O, 80°C	360	75
10	MP (0.05)	EtOH/H_2_O, 80°C	360	80
11	MP (0.05)	EtOH/H_2_O, 80°C	60	77
12	Fe_3_O_4_@Cs-MP (0.015)	H_2_O, r.t.	540	88
13	Fe_3_O_4_@Cs-MP (0.015)	EtOH, 50°C	360	75
14	Fe_3_O_4_@Cs-MP (0.02)	EtOH/H_2_O, 40°C	60	90
15	**Fe**_**3**_ **O**_**4**_ **@Cs-MP (0.02)**	**EtOH/H**_**2**_ **O, 50**°C	**30**	99
16	Fe_3_O_4_@Cs-MP (0.025)	EtOH/H_2_O, r.t.	30	45
17	Fe_3_O_4_@Cs-MP (0.025)	EtOH/H_2_O, 50°C	30	99

Bold values means that the most optimal conditions

**TABLE 3:** Optimization of reaction condition to prepare derivative **7a**.

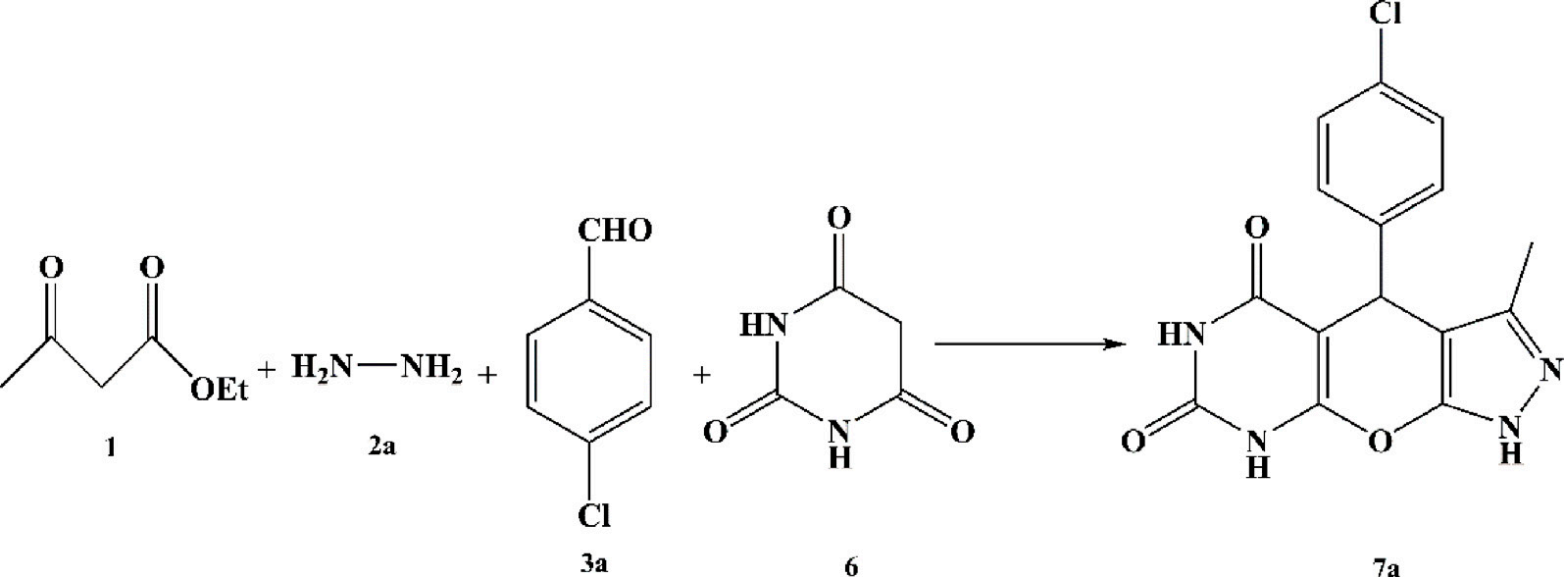				
Entry	Catalyst (g)	Condition	Time (min)	Yield (%)
1	-	EtOH, r.t.	360	10
2	-	H_2_O, r.t.	360	30
3	-	CH_3_CN, r.t.	360	15
4	-	EtOH, 80°C	360	-
5	-	DMSO, 80°C	360	22
6	Oleic acid (0.05)	EtOH, 80°C	360	52
7	ZnO (0.05)	EtOH, 80°C	360	80
8	Piperidine (0.05)	EtOH, 80°C	360	60
9	TiO_2_ (0.05)	EtOH/H_2_O, 80°C	360	91
10	Fe_3_O_4_ (0.05)	EtOH/H_2_O, 80°C	360	64
11	Fe_3_O_4_@Cs (0.05)	EtOH/H_2_O, 80°C	360	88
12	MP (0.05)	EtOH/H_2_O, 80°C	360	82
13	MP (0.05)	EtOH/H_2_O, 80°C	60	80
14	Fe_3_O_4_@Cs-MP (0.015)	H_2_O, r.t.	360	30
15	Fe_3_O_4_@Cs-MP (0.015)	EtOH, 50°C	300	82
16	Fe_3_O_4_@Cs-MP (0.02)	EtOH/H_2_O, 40°C	60	85
17	**Fe**_**3**_ **O**_**4**_ **@Cs-MP (0.02)**	**EtOH/H**_**2**_ **O, 50**°C	12	**99**
18	Fe_3_O_4_@Cs-MP (0.025)	EtOH/H_2_O, r.t.	12	65
19	Fe_3_O_4_@Cs-MP (0.025)	EtOH/H_2_O, 50°C	30	99

Bold values means that the most optimal conditions

During the choice of the best conditions, the reactions were first conducted in green solvents H_2_O and EtOH. The data presented in the Tables underscore the impact of catalysts on the reaction outcomes. It was observed that not using catalysts in different conditions resulted in low yields and prolonged reaction times. In contrast, the addition of common acidic, alkaline and nanoparticle catalysts, along with the temperature accretion, increased the yield and shortened the reaction time. Before dealing with the final structure, its motifs were evaluated separately as catalysts, but the process was imperfect. Use of MP as a distinct catalyst in both model reactions showed that MP had a noticeable effect on the reaction rate, but the products were not formed in excellent yields. Unintentional loss of products during catalyst recovery, and on the other hand, the absence of the porous surface of chitosan are possible factors of these results. Subsequently, varying amounts of Fe_3_O_4_@Cs-MP were utilized in the model reactions. The observations revealed that using 0.020 g of the catalyst in the solvents pair of the EtOH-H_2_O provided the most optimal conditions. According to existing literature in Supplementary Table S1, several catalysts, including nanocomposites with the porous surface, acidic or alkaline sites and ionic liquids have been reported as privileged catalysts in promoting target reactions. Albeit heterogeneous acidic catalysts are pioneers. Therefore, we have embedded all these active motifs into the design of the desired structure to exert the most significant effect on the activation of precursors and the progress of the processes. Since Fe_3_O_4_@Cs-MP nanocomposite is a magnetic catalyst, it is easy to recover, so it has a high yield and economic efficiency. Due to the presence of the chitosan matrix, it has a wide active surface to carry out the reaction and the presence of both acidic and alkaline sites has a significant effect on directing the process. We evaluated the catalytic role of the produced Fe_3_O_4_@Cs-MP nanocomposite. The results collated in Supplementary Table S1 highlight the superior efficacy of the Fe_3_O_4_@Cs-MP catalyst compared to previously employed catalysts in promoting the multicomponent synthesis of tetrahydropyrazolopyridine and pyrazolopyranopyrimidine compounds. Numerous new derivatives were synthesized using this catalyst. The synthesized derivatives, along with the yield and melting points, are detailed in Table 4, with the notable aspects being the disaffiliation of the yield and reaction time with the position and electronic nature of substitutions on the scaffolds of the products and the high yield of all derivatives, particularly the novel structures, owing to the use of the desired catalyst. Theoretically, however, structures with electron-withdrawing substituents are formed with relatively higher yields because they are more suitable for nucleophilic attack on the aldehyde during the reaction due to the lack of electrons.

**TABLE 4:** Scope of various tetrahydrodipyrazolopyridine (5a–p) and pyrazolopyranopyrimidine derivatives (7a–n) catalyzed by Fe_3_O_4_@Cs-MP.

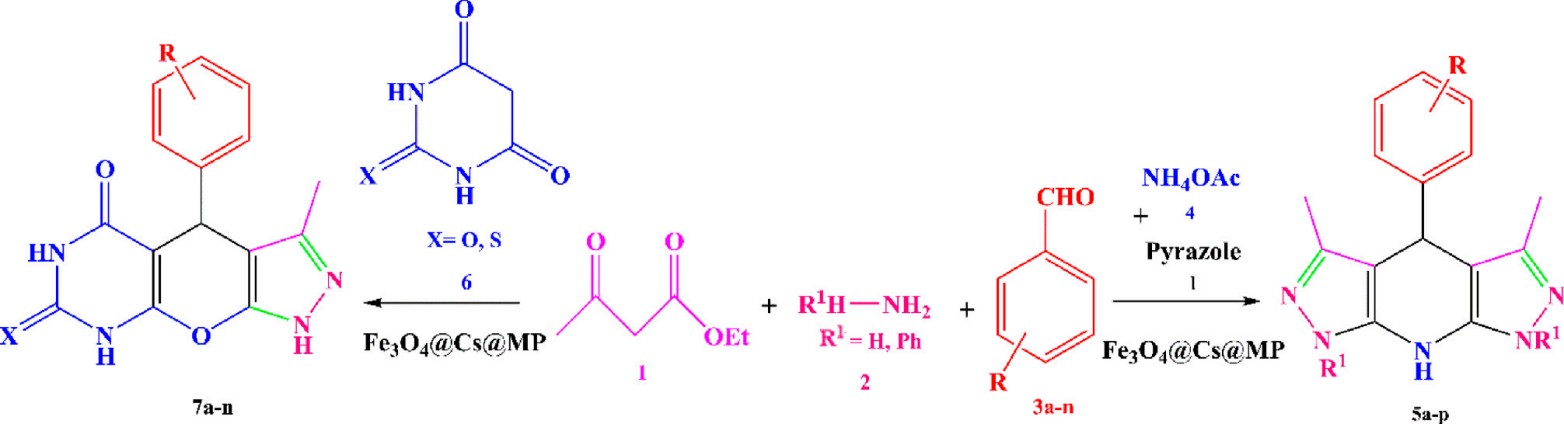		
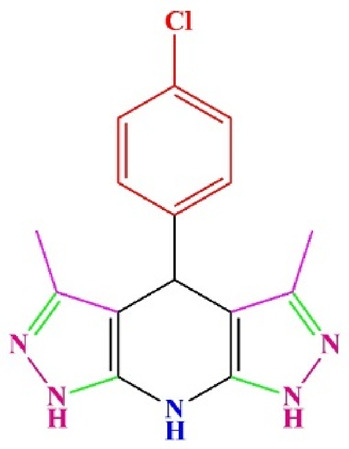 **5a** White powder Yield: 99%, Time: 30 min m.p.: 251°C–253°C ^[63]^ 251°C–253°C	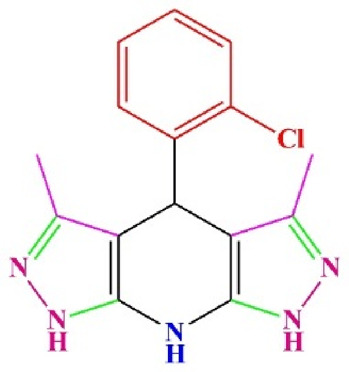 **5b** White powder Yield: 98%, Time: 35 min m.p.: 219°C–221°C ^[63]^ 218°C–221°C	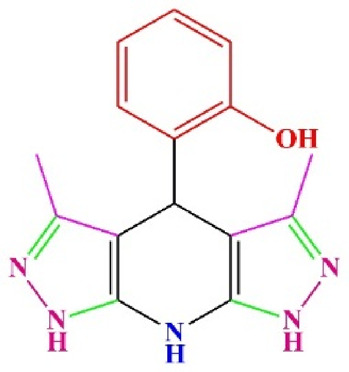 **5c** Yellow powder Yield: 92%, Time: 30 min m.p.: 208°C–210°C ^[70]^ 196°C–198°C
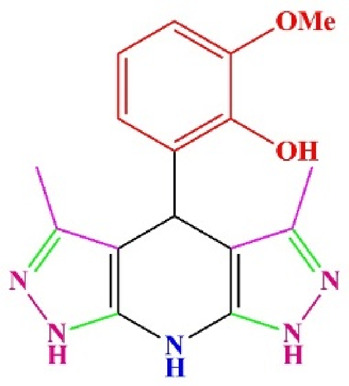 **5d** White powder Yield: 91%, Time: 30 min m.p.: 240°C–242°C ^[57]^ 240°C–242°C	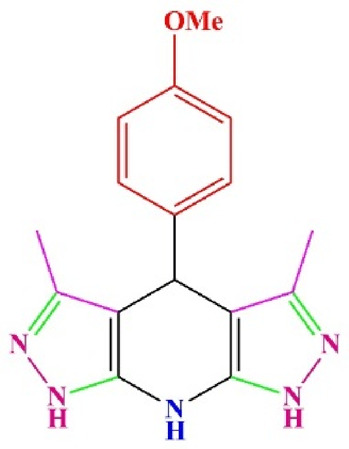 **5e** Yellow powder Yield: 88%, Time: 45 min m.p.: 190°C–192°C ^[71]^ 188°C–190°C	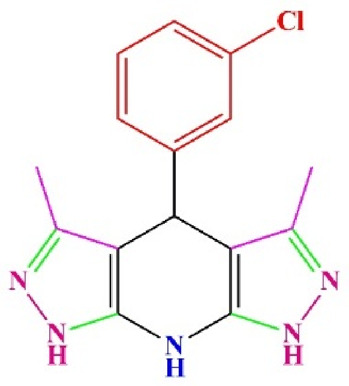 **5f** Yellow powder Yield: 89%, Time: 45 min m.p.: 220°C–222°C ^[71]^ 220°C–222°C
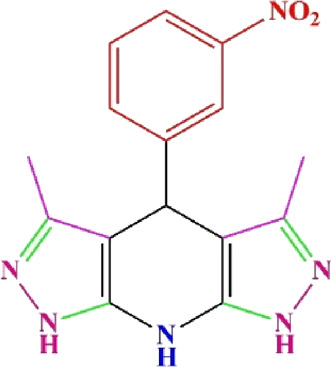 **5g** White powder Yield: 98%, Time: 35 min m.p.: 282°C–284°C^[71]^ 282–284°C	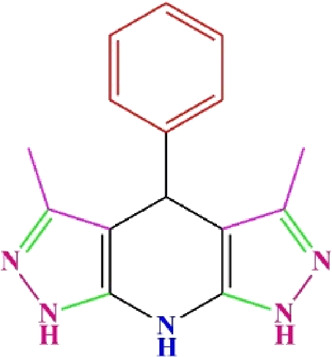 **5h** Yellow powder Yield: 99%, Time: 35 min m.p.: 240°C–242°C ^[54]^ 240°C–242°C	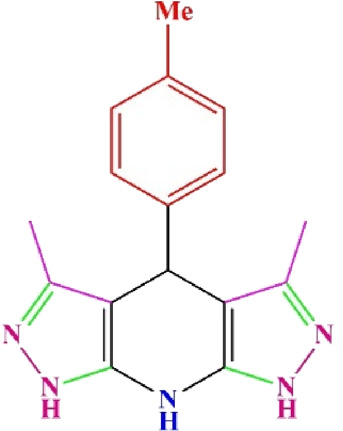 **5i** Yellow powder Yield: 97%, Time: 30 min m.p.: 240°C–243°C^[71]^243°C–245°C
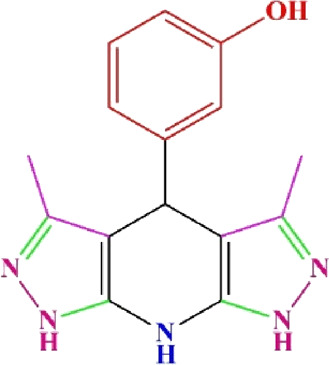 **5j** Yellow powder Yield: 80%, Time: 35 min m.p.: 255°C–257°C ^[72]^ 249°C–252°C	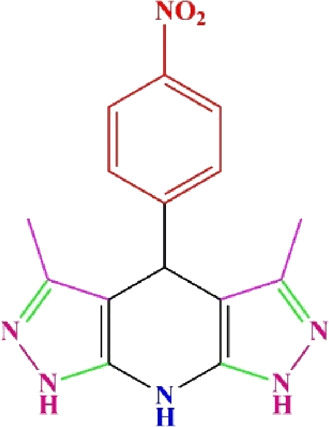 **5k** White powder Yield: 93%, Time: 31 min m.p.: 288°C–290°C^[57]^ 280°C–282°C	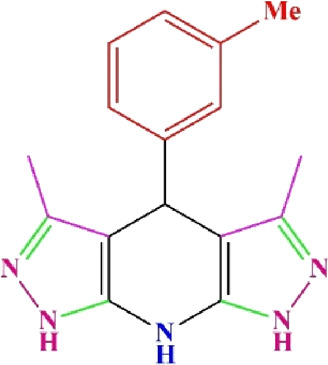 **5l** Yellow powder Yield: 93%, Time: 30 min m.p.: 279°C–280°C ^[72]^ 288°C–290°C
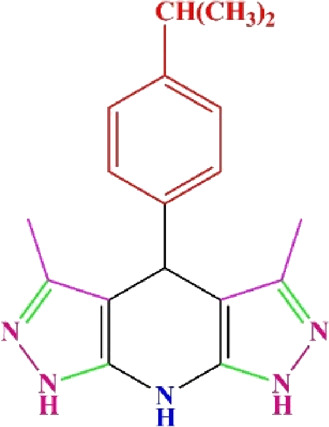 **5m** Yellow powder Yield: 93%, Time: 30 min m.p.: 248°C–250°C ^[72]^ 239°C–241°C	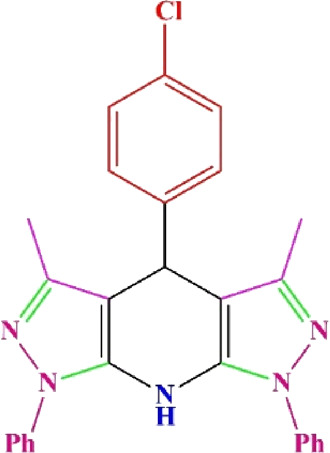 **5n** Yellow powder Yield: 97%, Time: 35 min m.p.: 231°C–233°C^[72]^ 228°C–231°C	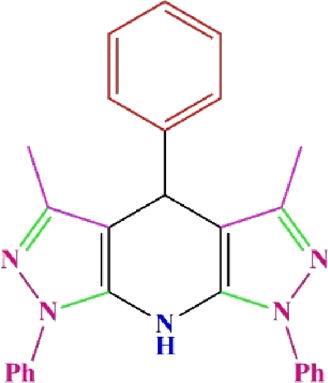 **5o** Yellow powder Yield: 84%, Time: 35 min m.p.: 187°C–189°C^[72]^ 186°C–188°C
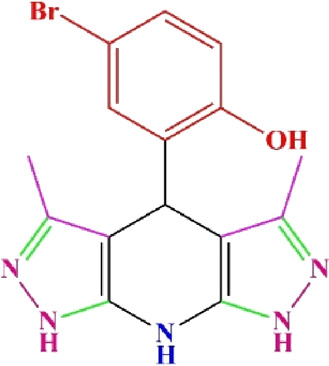 **5p** (New) Pale orange powder Yield: 95%, Time: 30 min m.p: 175°C–180°C	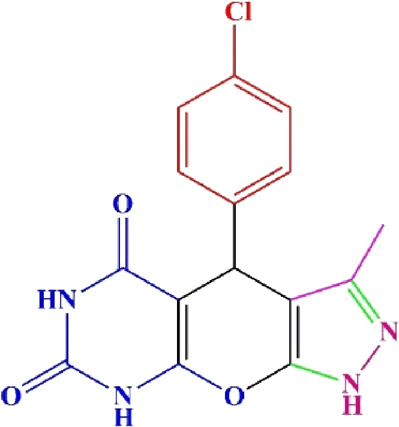 **7a** White powder Yield: 97%, Time: 12 min m.p.: 231°C–233°C ^[72]^ 230°C–232°C	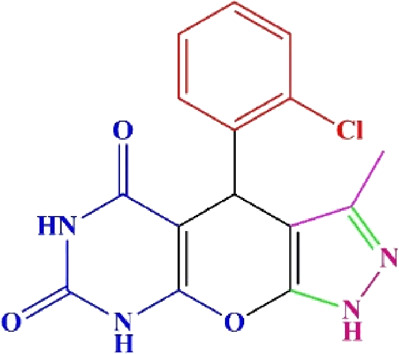 **7b** White powder Yield: 99%, Time: 12 min m.p.: 223°C–235°C ^[69]^ 229°C–231°C
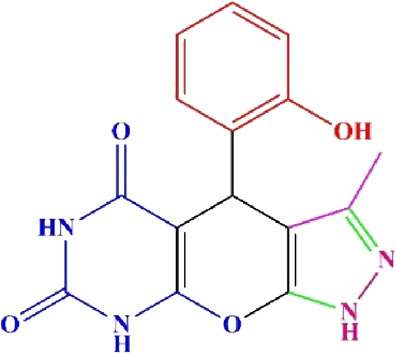 **7c** White powder Yield: 95%, Time: 15 min m.p.: 231°C–233°C ^[72]^ 229°C–231°C	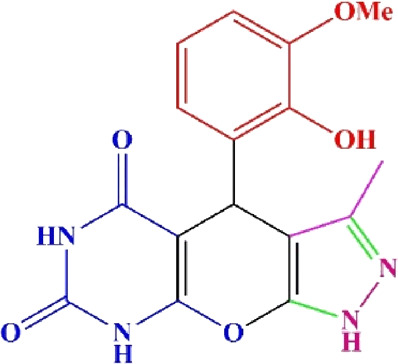 **7d** (New) White powder Yield: 99%, Time: 12 min m.p.: 176°C–180°C	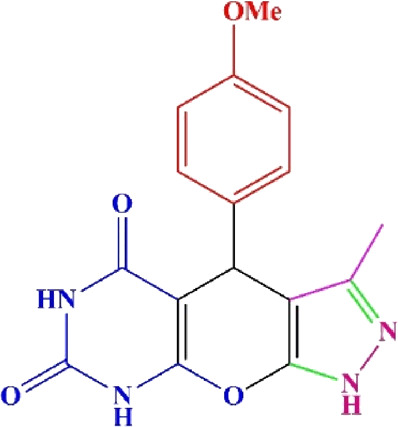 **7e** Yellow powder Yield: 94%, Time: 15 min m.p.: 228°C–230°C ^[72]^ 229°C–231°C
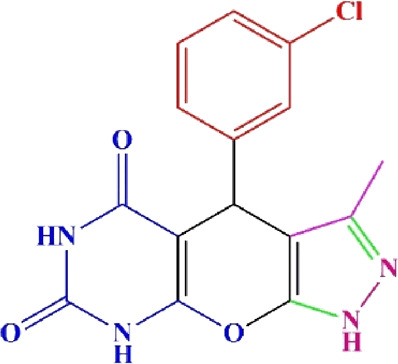 **7f** Pale Orange powder Yield: 93%, Time: 15 min m.p.: 301°C–303°C ^[73]^ 299°C–301°C	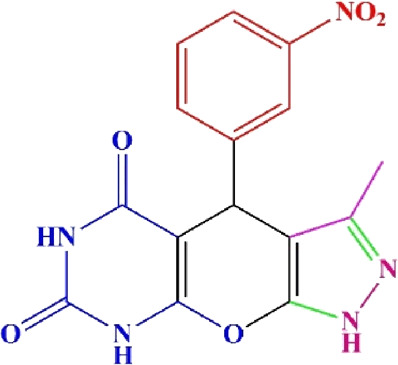 **7g** White powder Yield: 99%, Time: 12 min m.p.: 265°C–266°C^[71]^ 265°C–267°C	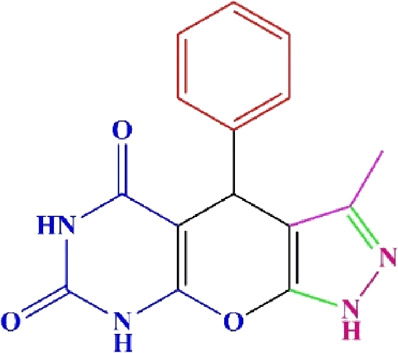 **7h** White powder Yield: 98%, Time: 12 min m.p.: 204°C–206°C ^[71]^ 203°C–206°C
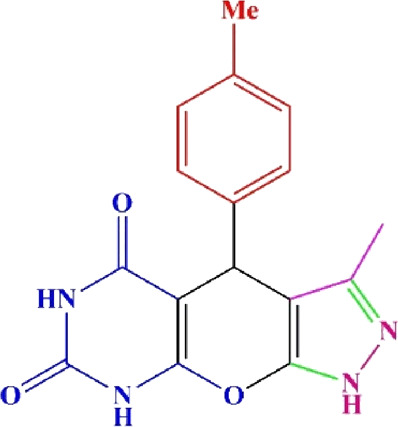 **7i** Yellow powder Yield: 98%, Time: 15 min m.p.: 204°C–206°C ^[71]^ 200°C–203°C	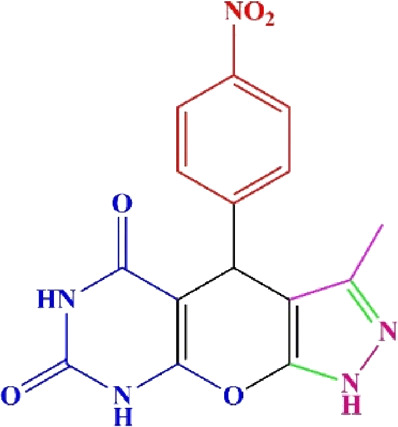 **7j** White powder Yield: 90%, Time: 12 min m.p.: 226°C–227°C ^[71]^ 225°C–228°C	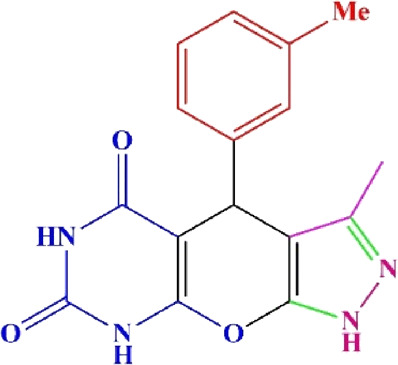 **7k (New)** Yellow powder Yield: 92%, Time: 13 min m.p.: 242°C–244°C
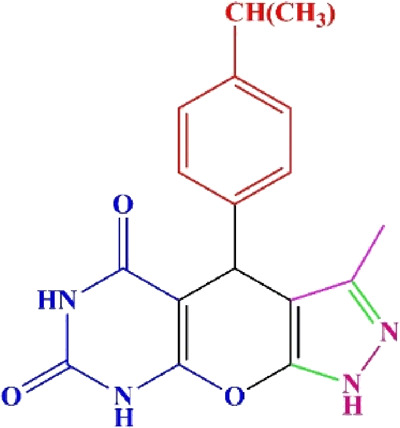 **7L** White powder Yield: 99%, Time: 13 min m.p.: 218°C–220 ^[73]^ 218°C–220°C	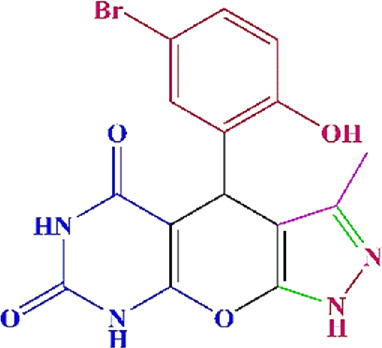 **7m (New)** Yellow powder Yield: 98%, Time: 15 min m.p.: 260°C–263°C	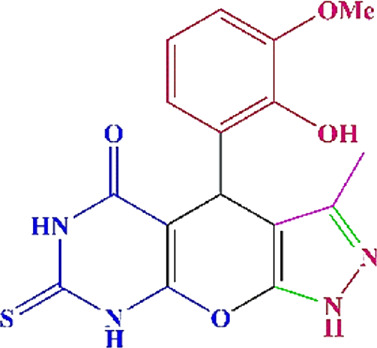 **7n (New)** Light yellow powder Yield: 96%, Time: 10 min m.p.: 252°C–254°C

**SCHEME 2:**
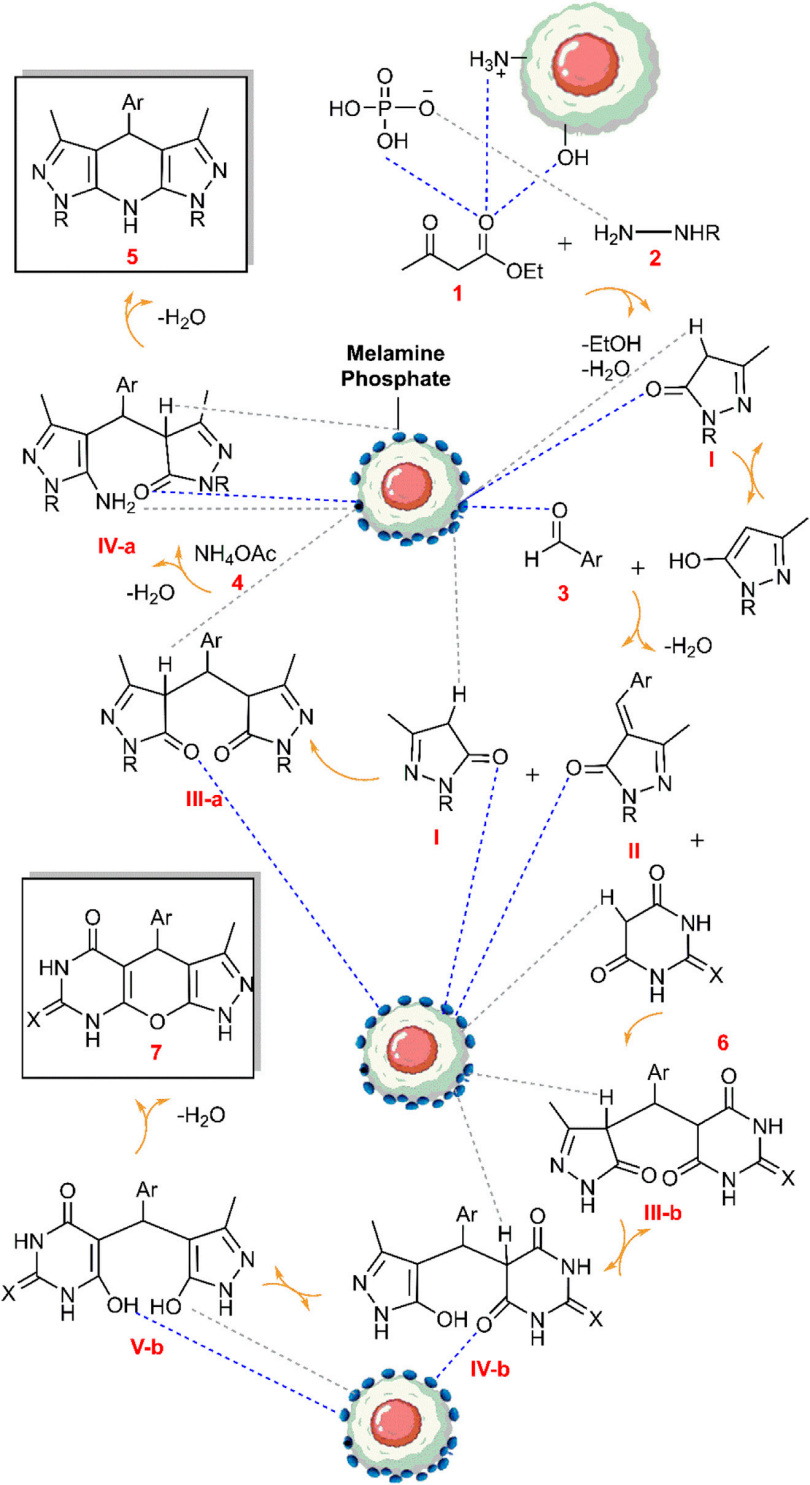
The probable mechanism aimed at the multicomponent synthesis of the various tetrahydrodipyrazolopyridine and the pyrazolopyranopyrimidine scaffolds catalyzed via the Fe3O4@Cs-MP composite.

The probable mechanism of the target multicomponent processes in the presence of the Fe_3_O_4_@Cs-MP catalyst is presented in Scheme 2. Initially, the pyrazole ring **(I)** is formed according to Knorr synthesis. The process followed by Knoevenagel condensation of the pyrazole tautomer with aldehyde led to the establishment of intermediate **(II)** involved in the Michael addition of another pyrazole molecule to form structure **(III-a).** Eventually by adding ammonium acetate **(4)** the pyridine ring **(5)** is formed after cyclization and remove water. Meanwhile, in another process, the entry of barbituric acid **(6)** in Michael addition instead of the pyrazole ring yields the intermediate **(III-b)** followed by tautomerization. In the last run, intramolecular cyclization and thereupon water exit lead to the synthesis of the heterocycle **(7)**. In this mechanism, as reflected in Scheme 2, it is assumed that the bifunctional Fe_3_O_4_@Cs-MP catalyst has bolstered the power of nucleophiles by its alkaline sites, whereby, the reaction is flowed. Also, the catalyst has activated the carbonyls and accelerated hydration via acidic positions. Furthermore, the proven capabilities of magnetic chitosan, especially a wide active surface have been influential in the current process. In these reactions, due to the use of an efficient catalyst, no use of excess amounts of precursors, the short reaction time and observing the adding sequence of the adducts, the only by-products are water and ethanol.

### Recycling of Fe_3_O_4_@Cs-MP

**FIGURE 10:**
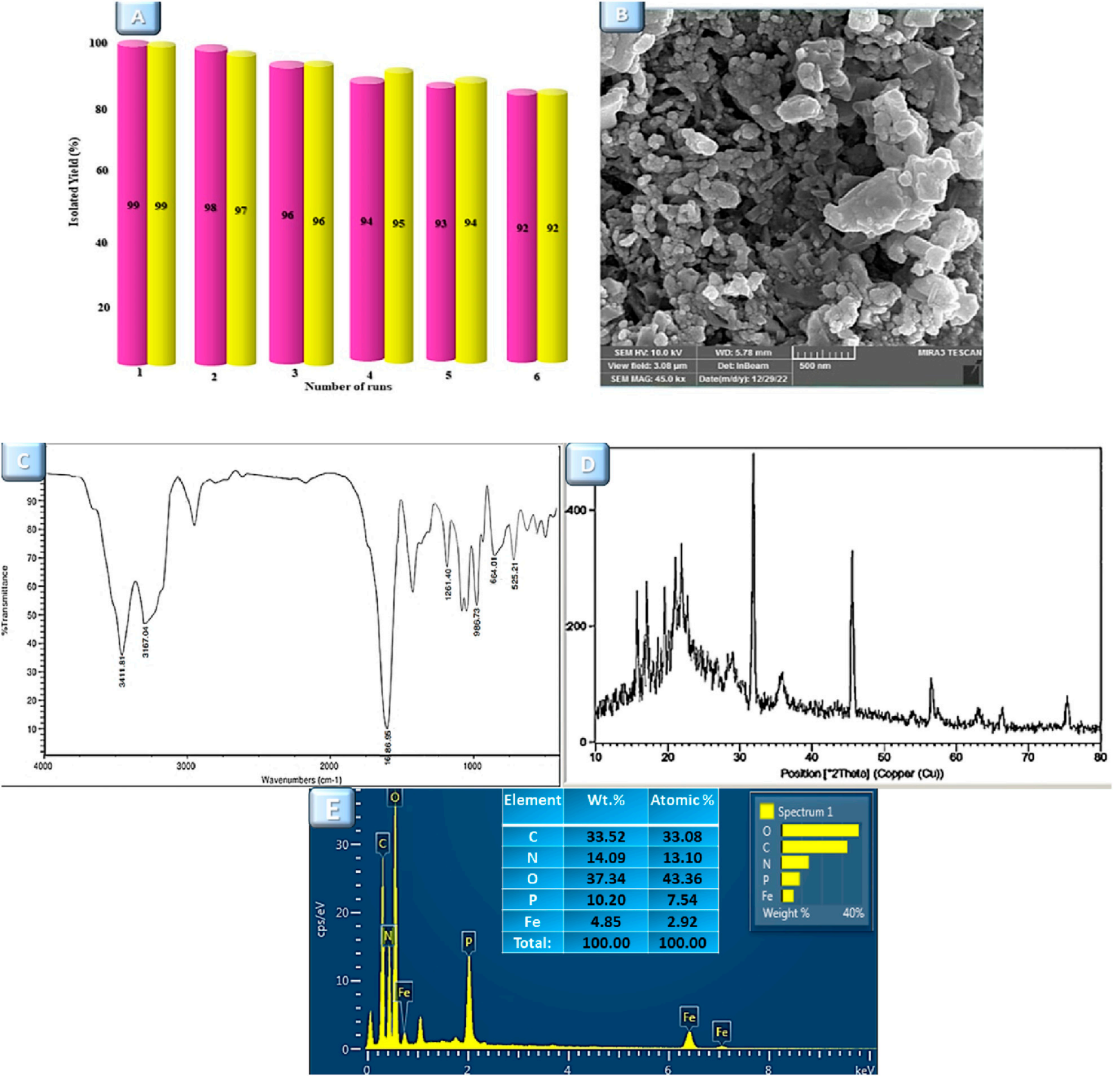
**(A)** Reusability of the Fe_3_O_4_@Cs-MP catalyst for the synthesis product 5a (purple charts) and 7a (yellow charts). **(B)** FE-SEM image, **(C)** IR spectrum, **(D)** XRD pattern, and **(E)** EDX analysis of recovered Fe_3_O_4_@Cs-MP catalyst.

One of the merits of each research is the capacity to expand and use it beyond the laboratory scope, requiring economic efficiency. The recyclability of the Fe_3_O_4_@Cs-MP catalyst was evaluated in both of the aforementioned multicomponent reactions. The magnetic property of this catalyst opened up an easy avenue to recover. In this manner, it was first recovered by an external magnet, and washed with ethanol then centrifuged and dried overnight. After retrieval, the catalyst was reused up to five times in the multicomponent reactions, demonstrating the catalyst’s reusability without significantly reducing its effectiveness. Then, it was characterized by several analyses. The outputs of these are shown in Figure 10. The characteristic peaks of the Fe_3_O_4_@Cs-MP catalyst is evident in the IR spectrum. Also, no significant difference was observed in the XRD pattern, SEM image, and EDX data of the recycled catalyst compared to the fresh sample. Moreover, the recovered catalyst from both MCRs was evaluated by ICP-MS. The outcomes of these tests displayed remarkably similar amounts of iron metal ere and after being used six times (Table 5); as a result, the iron nanoparticle is not significantly leached from the surface in exploitation processes.

**TABLE 5:** The Fe content in the intact Fe_3_O_4_@Cs-MP catalyst and recovered catalyst in both MCRs by ICP-MS.

Sample	ICP-MS %wt.
Intact catalyst	10.8% Fe
Recovered catalyst (for the synthesis product **5a**)	10.3% Fe
Recovered catalyst (for the synthesis product **7a**)	10.5% Fe

## Conclusion

The current research aims to underscore the significance of surface modification in biological structures. Along these lines, magnetic chitosan was modified with melamine phosphate ionic compound through covalent binding, resulting in a novel biocompatible catalyst. This modified catalyst was then assessed for its ability to facilitate multicomponent reactions, leading to the high-yield production of new and diverse derivatives of tetrahydropyrazolopyridine and pyrazolopyrimidine. Due to its recyclable structure and active functional groups, this biocompatible composite is anticipated to exhibit broader utility in various organic reactions for the synthesis of bioactive compounds. The properties of this modified catalyst, including its biocompatibility, recyclability, high thermal stability and the potential for high-yield production of diverse derivatives, not only signify a significant advancement in catalyst design but also hold promise for the development of efficient and sustainable processes for generating valuable bioactive compounds.

## Data Availability

The original contributions presented in the study are included in the article/Supplementary Material, further inquiries can be directed to the corresponding author.
