# Supplementary material for: Melamine phosphate-modified magnetic chitosan: a novel biocompatible catalyst for the synthesis of biological tetrahydrodipyrazolopyridine and pyrazolopyranopyrimidine derivatives
Source: Front Chem. 2024 May 15;12:1395008. doi: 10.3389/fchem.2024.1395008 (PMC11134575; doi:10.3389/fchem.2024.1395008)
Supplement: Supplementary file 1 [file DataSheet1.docx]

**Melamine phosphate-modified magnetic chitosan: A novel biocompatible catalyst for the synthesis of biological tetrahydrodipyrazolopyridine and pyrazolopyranopyrimidine derivatives**

**Maryam Mousavi-Ebadi^1^, Javad Safaei-Ghomi^1^***

*^1^Department of Organic Chemistry, Faculty of Chemistry, University of Kashan, Kashan, 51167, I. R. Iran*

**Corresponding author, Tel: +98 31 55912385; E-mail: safaei@kashanu.ac.ir*

**Section a) Supplementary information on catalyst preparation**


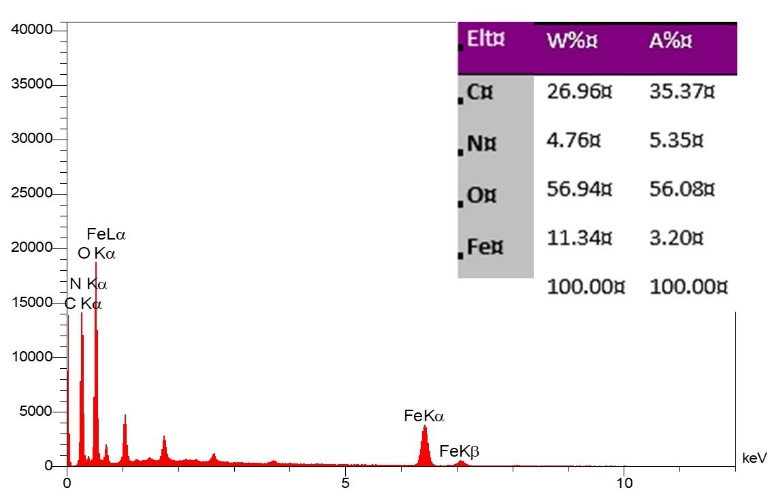

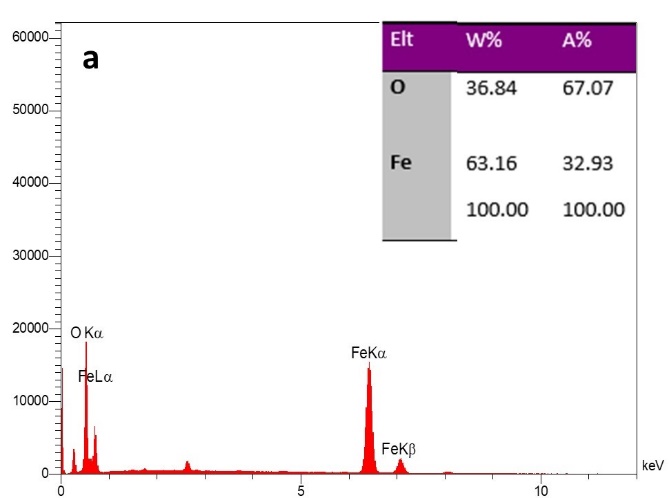


**b**

**FIGURE S1.** EDX analysis of Fe_3_O_4_ (a) and Fe_3_O_4_@Cs (b)


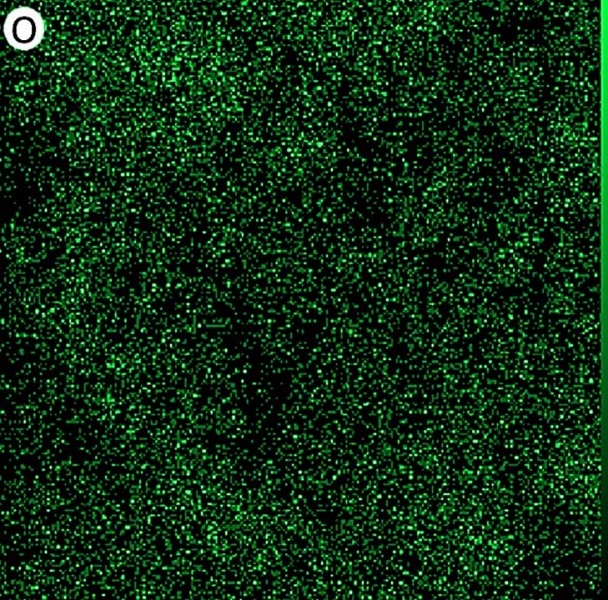

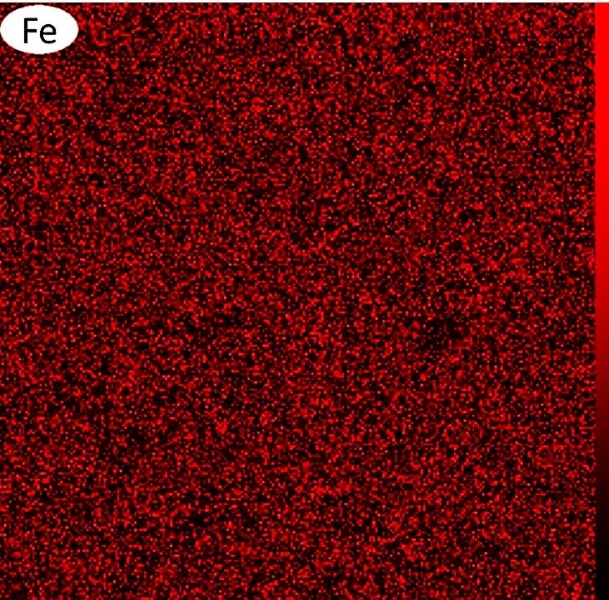


**FIGURE S2.** Elemental mapping of the Fe_3_O_4_


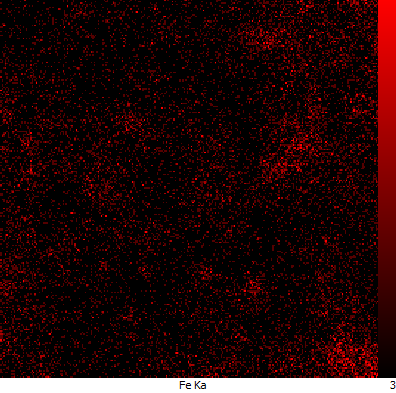

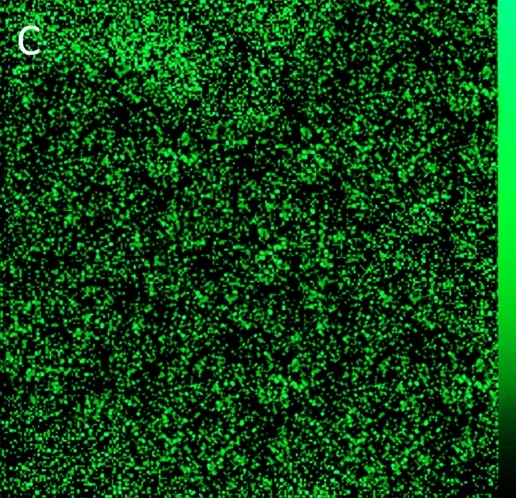

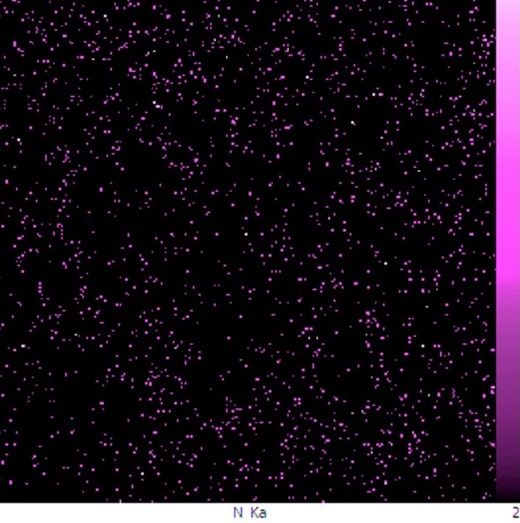


Fe

N

Na


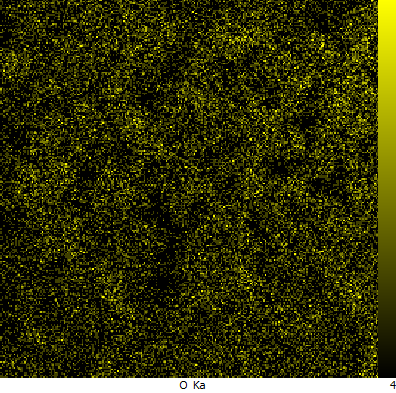

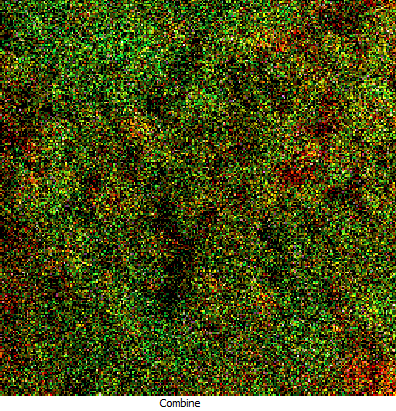


O

Combine

O

N

Fev

O

**FIGURE S3.** Elemental mapping of the Fe_3_O_4_@Cs

**TABLE S1.** Review and compare the effectiveness of catalyst Fe_3_O_4_@Cs-MP with reported catalysts used for the synthesis of products 5a and 7a, mentioning the reaction conditions.

| Ref. | Yield  (%) ^b^ | Time  (min) | N source  (mol) | Condition | Catalyst (g) | Product | Entry |
| --- | --- | --- | --- | --- | --- | --- | --- |
| (Mohammadi Ziarani. 2022) | 81 | 8 | NH_4_OAC (0.003) | Neat, 120^ᵒ^C | Fe_3_O_4_SiO_2_(BuSO_3_H) (0.02) | 5a | 1 |
| (Omidi, 2022) | 90 | 10 | NH_4_OAC (0.001) | EtOH, r.t. | (MnCoFe_2_O_4-_Niacin SO_3_H)^+^Cl^-^(0.15) | 5a | 2 |
| (Shahbazi-Alavi, 2016) | 92 | 45 | NH_4_OAC (0.004) | EtOH, r.t. | CuCr_2_O_4_ (0.1) | 5a | 3 |
| (Salehi, 2018) | 93 | 45 | NH_4_OAC (0.003) | H_2_O, 55 ᵒC | Nano-ovalbumin (0.05) | 5a | 4 |
| (Safaei-Ghomi, 2016) | 94 | 40 | NH_4_OAC (0.003) | EtOH, 80 ᵒC | nano-CdZr_4_(PO_4_)_6_ (0.06) | 5a | 5 |
| (Nagasundaram, 2023) | 95 | 30 | NH_4_OAC (0.003) | EtOH, LED, r.t. | eosin Y (0.02) | 5a | 6 |
| (Vanegas, 2019) | 96 | 30 | NH_4_OAc (0.002) | Neat, 110 ᵒC | ChCl: Urea (0.8) | 5a | 7 |
| (Tamaddon, 2019) | 96 | 70 | CO(NH_2_)_2_  (0.006) | H_2_O, 70 ᵒ | CNCD@Urea (0.01) | 5a | 8 |
| (Azizi, 2019) | 98 | 30 | NH_4_OAc (0.004) | EtOH, 80 ᵒC | KCC-1-NH_2_-DPA (0.1) | 5a | 9 |
| **This work** | **99** | **30** | NH_4_OAc (0.001) | **EtOH/H_2_O, 50** ᵒC | **Fe_3_O_4_@Cs-MP (0.02)** | **5a** | **10** |
| (Maleki, 2017) | 89 | 30 | - | H_2_O, r.t. | Fe_3_O_4_/cellulose (0.005) | 7a | 11 |
| (Lotfian, 2020) | 92 | 18 | - | H_2_O, 80 ^ᵒ^C, US | [Ga(W_5_O_18_)^2^] ^9-^(0.1) | 7a | 12 |
| (Rana, 2015) | 92 | 50 | - | EtOH, 80 ^ᵒ^C | Diamino@GO (0.04) | 7a | 13 |
| (Ziarani, 2018) | 92 | 10 | - | H_2_O, 100 | SBA-Pr-SO_3_H (0.02) | 7a | 14 |
| (Yekke-Ghasemi, 2018) | 95 | 75 | - | H_2_O, 80 ^ᵒ^C | (TBA)_6_BZrW_11_ (0.02) | 7a | 15 |
| (Karrabi, 2023) | 95 | 35 | - | H_2_O, 80 ^ᵒ^C | CuO-Mont/SiO_2_-EPiMl-SO_3_H (0.03) | 7a | 16 |
| (Sadjadi, 2017) | 96 | 35 | - | H_2_O, 100 | H_3_PW_12_O_40_@fs (0.03) | 7a | 17 |
| (Tipale, 2018) | 96 | 30 | - | EtOH, 80 ^ᵒ^C | ChCl: Urea (0.8) | 7a | 18 |
| (Amini, 2021) | 96 | 15 | - | H_2_O, 100 ^ᵒ^C | ZnFe_2_O_4_/GA (0.01) | 7a | 19 |
| **This work** | **99** | **12** |  | **EtOH/H_2_O,** 50 ^ᵒ^C | **Fe_3_O_4_@Cs-MP (0.02)** | **7a** | **20** |

^a^ Reaction condition for the synthesis of derivatives 5: a mixture of Ethyl 3-oxo butanoate (2 mmol), Hydrazine (2 mmol), aldehyde derivative (1 mmol), solvent (4 ml) and catalyst were reacted.

Reaction condition for the synthesis of derivatives 7: a mixture of ethyl 3-oxobutanoate (1 mmol), hydrazine hydrate (1 mmol), aldehyde derivative (1 mmol), (thio) barbituric acid (1 mmol), solvent (4 ml) and catalyst were reacted.

^b^ Isolated yields.

**Section b) Specteral and Physical data of organic derivatives**

**4-(4-chlorophenyl)-3, 5-dimethyl-1,4,7,8-tetrahydrodipyrazolo[3,4-b:4',3'-e]pyridine (*5a*):** White solid; m.p.: 251-253 °C; FT-IR (KBr) (ν_max_/cm^-1^): 3265, 3099, 1604, 1526, 8011; ^1^H NMR (400 MHz, DMSO-*d*_6_) δ (ppm): 11.37 (s, 3H, NH), 7.28 (d, J = 8 Hz, 2H, Ar), 7.14 (d, J = 8 Hz, 2H, Ar), 4.83 (s, 1H), 2.08 (s, 6H). ^13^C NMR (400 MHz, DMSO) δ 161.8, 134.4, 127.3, 124.9, 120.9, 120.0, 111.3, 37.0, 14.5. Anal. Cal. for C_16_H_18_ClN_5_: C: 60.85 %, H: 5.75 %, N: 11.23, Found: C: 69.47 %, H: 5.04%, N: 10.99%.

**4-(2-chlorophenyl)-3,5-dimethyl-1,4,7,8-tetrahydrodipyrazolo[3,4-b:4',3'-e]pyridine (*5b*):** White solid; m.p.: 218-221°C; FT-IR (KBr) (ν_max_/cm^-1^): 3245, 3219, 1649, 1567, 8219; ^1^H NMR (400 MHz, DMSO-*d*_6_) δ (ppm): 11.07 (s, 3H, NH), 7.15-7.75 (m, 4H, Ar), 5.08 (s, 1H), 1.95 (s, 6H). ^13^C NMR (400 MHz, DMSO) δ 157.6, 146.4, 139.5, 130.1, 129.7, 129.4, 128.2, 120.0, 103.4, 33.9, 10.4. Anal. Cal. for C_16_H_18_ClN_5_: C: 75.98 %, H: 5.37 %, N: 18.56 %, Found: C: 75.87 %, H: 5.04%, N: 17.99%.

**2-(3,5-dimethyl-1,4,7,8-tetrahydrodipyrazolo[3,4-b:4',3'-e]pyridin-4-yl)phenol (*5c*):** Yellow solid; m.p.: 196-198°C; FT-IR (KBr) (ν_max_/cm^-1^): 3479, 3333, 2947, 2869, 2128, 1703, 1637, 14387; 1229, ^1^H NMR (400 MHz, DMSO-*d*_6_) δ (ppm): 10.68 (s, 3H, NH), 9.02 (s, 1H, OH), 6.94-7.41 (m, 3H, Ar), 5.39 (s, 1H), 2.97 (s,6H). ^13^C NMR (400 MHz, DMSO) δ 162.2, 154.1, 128.7, 125.8, 125.4, 124.2, 104.8, 111.6, 34.0, 17.8. Anal. Cal. for C_16_H_19_N_5_O: C: 64.63 %, H: 6.44 %, N: 23.55, Found: C: 64.42 %, H: 5.94%, N: 23.18%.

**2-(3,5-dimethyl-1,4,7,8-tetrahydrodipyrazolo[3,4-b:4',3'-e]pyridin-4-yl)-6-methoxyphenol (*5d*):** White solid; m.p.: 240-242°C; FT-IR (KBr) (ν_max_/cm^-1^): 3472, 33578, 2935, 1685, 1436; ^1^H NMR (400 MHz, DMSO-*d*_6_) δ (ppm): 11.54 (s, 3H, NH), 10.22 (s, 1H, OH), 7.54-7.95 (m, 3H, Ar), 4.97 (s,1H), 4.75 (s,3H), 2.08 (s, 6H). ^13^C NMR (400 MHz, DMSO) δ 161.5, 154.8, 146.4, 139.8, 129.6, 124.7, 120.2, 119.7, 112.5, 103.8, 67.3, 36.1, 15.3. Anal. Cal. for C_17_H_21_N_5_O_2_: C: 62.37 %, H: 6.27 %, N: 21.39, Found: C: 61.97 %, H: 5.95%, N: 21.16%.

**4-(4-methoxyphenyl)-3,5-dimethyl-1,4,7,8-tetrahydrodipyrazolo[3,4-b:4',3'-e]pyridine (*5e*):** Yellow solid; m.p.: 188-190 °C; FT-IR (KBr) (ν_max_/cm^-1^): 3536, 3259, 1635, 1417; 1330, 1200. ^1^H NMR (400 MHz, DMSO-*d*_6_) δ (ppm): 13.11 (s, 3H, NH), 7.66 (d, *J*=8 Hz, 2H, Ar), 7.91 (d, *J*= 8Hz, 2H, Ar), 5.80 (s, 1H), 4.61 (s, 3H, OMe), 2.11 (s, 6H). ^13^C NMR (400 MHz, DMSO) δ 163.6, 152.2, 148.9, 137.8, 130.5, 129.1, 128.0, 125.7, 106.5, 63.9, 29.2, 19.3. Anal. Cal. for C_16_H_17_N_5_O: C: 65.07%, H: 5.8 %, N: 23.71, Found: C: 64.9 %, H: 5.85%, N: 23.77%.

**4-(3-chlorophenyl)-3,5-dimethyl-1,4,7,8-tetrahydrodipyrazolo[3,4-b:4',3'-e]pyridine (*5f*):** Yellow solid; m.p.: 220-222 °C; FT-IR (KBr) (ν_max_/cm^-1^): 3259, 1635, 1417; 1330, 1200, 824. ^1^H NMR (400 MHz, DMSO-*d*_6_) δ (ppm): 13.11 (s, 3H, NH), 7.66 (d, *J*=8 Hz, 2H, Ar), 7.91 (d, *J*= 8Hz, 2H, Ar), 5.80 (s, 1H), 4.61 (s, 3H, OMe), 2.11 (s, 6H). ^13^C NMR (400 MHz, DMSO) δ 156.1, 140.1, 137.8, 134.2, 130.1, 127.1, 124.0, 122.6, 112.3, 52.5, 11.6. Anal. Cal. for C_15_H_14_ClN_5_: C: 60.1%, H: 4.71 %, N: 23.36, Found: C: 60.82 %, H: 4.8%, N: 23.27%.

**3,5-dimethyl-4-(3-nitrophenyl)-1,4,7,8-tetrahydrodipyrazolo[3,4-b:4',3'-e]pyridine (*5g*):** Yellow solid; m.p.: 282-284 °C; FT-IR (KBr) (ν_max_/cm^-1^): 3299, 1610, 1588, 1420, 1223. ^1^H NMR (400 MHz, DMSO-*d*_6_) δ (ppm): 13.91 (s, 3H, NH), 7.70 (s, 1H, Ar), 7.25-7.69 (m, 3H, Ar), 4.97 (s, 1H), 2.09 (s, 6H.). ^13^C NMR (400 MHz, DMSO) δ 167.6, 149.8, 147.4, 145.3, 144.0, 136.2, 130.5, 123.5, 48.2, 19.7. Anal. Cal. for C_15_H_14_N_6_O_2_: C: 58.06%, H: 4.55 %, N: 27.08, Found: C: 58.02 %, H: 4.46%, N: 27.1%.

**3,5-dimethyl-4-phenyl-1,4,7,8-tetrahydrodipyrazolo[3,4-b:4',3'-e]pyridine (*5h*):** Pale Yellow solid; m.p.: 240-242°C; FT-IR (KBr) (ν_max_/cm^-1^): 3268, 1668, 14184; ^1^H NMR (400 MHz, DMSO-*d*_6_) δ (ppm): 11.36 (s, 3H, NH), 7.12-7.73 (m, 5H, Ar), 4.82 (s, 1H), 2.08 (s, 6H.). ^13^C NMR (400 MHz, DMSO) δ 156.2, 148.7, 128.3, 125.1, 114.1, 104.7, 33.9, 10.3. Anal. Cal. for C_15_H_15_N_5_: C: 67.9%, H: 5.7 %, N: 26.4, Found: C: 67.83 %, H: 5.86%, N: 26.31%.

**3,5-dimethyl-4-(p-tolyl)-1,4,7,8-tetrahydrodipyrazolo[3,4-b:4',3'-e]pyridine (5i):** Yellow solid; m.p.: 240-242°C; FT-IR (KBr) (ν_max_/cm^-1^): 3191, 3129, 2949, 2214, 1647, 1491; ^1^H NMR (400 MHz, DMSO-*d*_6_) δ (ppm): 13.91 (s, 3H, NH), 8.03 (d, *J*=8 Hz, 2H, Ar), 7.55 (d, *J*=8 Hz, 2H, Ar), 4.98 (s, 1H), 3.30 (s, 3H), 2.11 (s*,* 6H). ^13^C NMR (400 MHz, DMSO) δ 159.6, 127.2, 126.1, 122.5, 120.1, 105.8, 35.3, 21.3, 17.6. Anal. Cal. for C_17_H_21_N_5_: C: 69.12%, H: 7.17 %, N: 23.71, Found: C: 69.21 %, H: 6.99%, N: 23.80%.

**3-(3,5-dimethyl-1,4,7,8-tetrahydrodipyrazolo[3,4-b:4',3'-e]pyridin-4-yl)phenol (*5j*):** Yellow solid; m.p.: 249-252 °C; FT-IR (KBr) (ν_max_/cm^-1^): 3419, 3222, 1675, 1452; 1246, 1111. ^1^H NMR (400 MHz, DMSO-*d*_6_) δ (ppm): 13.91 (s, 3H, NH), 9.98 (s, OH), 7.70 (s, 1H, Ar), 7.25-7.47 (m, 3H, Ar), 5.05 (s, 1H), 2.05 (s, 6H). ^13^C NMR (400 MHz, DMSO) δ 167.8, 149.0, 146.1, 138.6, 128.1, 126.7, 110.7, 39.8, 15.7. Anal. Cal. for C_16_H_19_N_5_O: C: 64.63%, H: 6.44 %, N: 23.55, Found: C: 64.86 %, H: 6.85%, N: 23.57%.

**3,5-dimethyl-4-(4-nitrophenyl)-1,4,7,8-tetrahydrodipyrazolo[3,4-b:4',3'-e]pyridine (*5k*):** White solid; m.p.: 280-282°C; FT-IR (KBr) (ν_max_/cm^-1^): 3359, 2221, 1626, 1467, 845, 810; ^1^H NMR (400 MHz, DMSO-*d*_6_) δ (ppm): 11.25 (s, 3H, NH), 8.09 (d, *J*= 8Hz, 2H, Ar), 7.35 (d, *J*= 8Hz, 2H, Ar), 4.95 (s, 1H), 2.07 (s, 6H). ^13^C NMR (400 MHz, DMSO) δ 148.7, 132.2, 131.9, 130.3, 129.8, 129.5, 113.3, 51.0, 28.7. Anal. Cal. for C_16_H_18_N_6_O_2_: C: 58.88%, H: 5.56 %, N: 25.75, Found: C: 58.82 %, H: 5.62%, N: 25.55%.

**3,5-dimethyl-4-(m-tolyl)-1,4,7,8-tetrahydrodipyrazolo[3,4-b:4',3'-e]pyridine (*5l*):** Yellow solid; m.p.: 288-290°C; FT-IR (KBr) (ν_max_/cm^-1^): 3221, 2834, 1684, 1463; 1298, 1123. ^1^H NMR (400 MHz, DMSO-*d*_6_) δ (ppm): 11.07 (s, 3H, NH), 7.03-7.34 (m, 3H, Ar), 6.94 (s, 1H, Ar), 4.80 (s, 1H), 2.16 (s, 6H); 2.13 (s, 3H). ^13^C NMR (400 MHz, DMSO) δ 163.1, 131.1, 130.9, 129.7, 127.3, 125.8, 123.0, 111.5, 110.4, 36.2, 18.4, 9.6. Anal. Cal. for C_17_H_21_N_5_: C: 69.12%, H: 7.17 %, N: 23.71, Found: C: 69.23 %, H: 7.22%, N: 23.55%.

**4-(4-isopropylphenyl)-3,5-dimethyl-1,4,7,8-tetrahydrodipyrazolo[3,4-b:4',3'-e]pyridine (*5m*):** Yellow solid; m.p.: 239-241°C; FT-IR (KBr) (ν_max_/cm^-1^): 3315, 3001, 2945, 2344, 1663, 1432; 1212. ^1^H NMR (400 MHz, DMSO-*d*_6_) δ (ppm): 12.84 (s, 3H, NH), 7.06-7.08 (d, *J*= 8 Hz, 2H, Ar), 7.12-7.14 (d, *J*=8 Hz, 2H, Ar), 5.00 (s, 1H), 2.76-2.86 (m, 1H), 2.09 (s, 6H), ^13^C NMR (400 MHz, DMSO) δ 159.4, 125.5, 125.4, 124.7, 105.4, 91.3, 35.2, 15.9, 14.5, 9.4. 1.15-1.23 (d, *J*=16 Hz, 6H). Anal. Cal. for C_19_H_25_N_5_: C: 70.56%, H: 7.79 %, N: 21.65%, Found: C: 70.09 %, H: 7.29%, N: 22.62%.

**4-(4-chlorophenyl)-3,5-dimethyl-1,4,7,8-tetrahydrodipyrazolo[3,4-b:4',3'-e]pyridine (*5n*):** Pale yellow solid; m.p.: 228-231°C; FT-IR (KBr) (ν_max_/cm^-1^): 3369, 3041, 2009, 1635, 1411; 824. ^1^H NMR (400 MHz, DMSO-*d*_6_) δ (ppm): 13.91 (s, 3H, NH), 7.72-7.25 (m, 14H, Ar), 4.97 (s, 1H), 2.32 (s, 6H). ^13^C NMR (400 MHz, DMSO) δ 157.9, 152.2, 147.4, 146.4, 145.4, 139.6, 104.6, 101.0, 33.8, 12.5. Anal. Cal. for C_28_H_26_ClN_5_: C: 71.86%, H: 5.57 %, N: 14.96%, Found: C: 72.03 %, H: 5.69%, N: 22.68%.

**3,5-dimethyl-1,4,7-triphenyl-1,4,7,8-tetrahydrodipyrazolo[3,4-b:4',3'-e]pyridine (*5o*):** Pale yellow solid; m.p.: 186-188°C; FT-IR (KBr) (ν_max_/cm^-1^): 3360, 3107, 2948, 1620, 1417. ^1^H NMR (400 MHz, DMSO-*d*_6_) δ (ppm): 13.91 (s, 1H, NH), 7.46-7.80 (m, 15H, Ar), 4.73 (s, 1H), 2.15 (s, 6H). ^13^C NMR (400 MHz, DMSO) δ 160.5, 150.5, 144.7, 141.1, 132.8, 130.4, 129.3, 128.7, 128.0, 127.5, 126.0, 125.5, 124.1, 104.7, 80.0, 30.9, 10.7. Anal. Cal. for C_27_H_23_N_5_: C: 77.67%, H: 5.55 %, N: 16.77%, Found: C: 78.01 %, H: 5.64%, N: 16.35%.

**4-bromo-2-(3,5-dimethyl-1,4,7,8-tetrahydrodipyrazolo[3,4-b:4',3'-e]pyridin-4-yl)phenol (*5p*):** Pale orange solid; m.p.: 175-180°C; FT-IR (KBr) (ν_max_/cm^-1^): 3443, 2921, 2864, 1624, 1477, 1287; 1182, 460. ^1^H NMR (400 MHz, DMSO-*d*_6_) δ (ppm): 11.17 (s, 3H, NH), 10.35 (s, OH), 7.57 (s, 1H, Ar), 7.22-7.43 (m, 2H, Ar), 7.12-7.14 (d, *J*=8 Hz, 2H, Ar), 4.99 (s, 1H), 2.02 (s, 6H). ^13^C NMR (400 MHz, DMSO) δ:164.9, 161.2, 158.1, 140.0, 139.9, 139.2, 135.9, 132.0, 121.0, 119.3, 111.0, 101.2, 38.9, 10.9, 10.4. Anal. Cal. for C_15_H_14_BrN_5_O. Found: C: 50.22%, H: 3.97 %, N: 19.41%. MS (m/z): calcd: 359.0, Obs. MS m/z: 359.3 (M+).

**4-(4-chlorophenyl)-3-methyl-6,8-dihydropyrazolo[4',3':5,6]pyrano[2,3-d]pyrimidine-5,7(1H,4H)-dione (*7a*):** White solid; m.p.: 230-232°C; FT-IR (KBr) (ν_max_/cm^-1^): 3428, 2923, 1623, 1589; 860. ^1^H NMR (400 MHz, DMSO-*d*_6_) δ (ppm): 13.09 (s, 1H, NH), 10.20 (s, 2H, NH), 7.26-7.28 (d, *J*= 8 Hz, 2H, Ar), 7.09-7.07 (d, *J*=8 Hz, 2H, Ar), 5.41 (s, 1H), 2.23 (s, 3H). ^13^C NMR (400 MHz, DMSO) δ 165.4, 163.6, 155.8, 139.4, 135.1, 124.9, 109.1, 101.4, 87.8, 34.2, 16.2. Anal. Cal. for C_15_H_11_ClN_4_O_3_. C: 54.48%, H: 3.35 %, N: 16.94%. Found: C: 54.32%, H: 3.46 %, N: 16.99%.

**4-(2-chlorophenyl)-3-methyl-6,8-dihydropyrazolo[4',3':5,6]pyrano[2,3-d]pyrimidine-5,7(1H,4H)-dione (*7b*):** White solid; m.p.: 229-231°C; FT-IR (KBr) (ν_max_/cm^-1^): 3476, 2924, 1706, 1636, 1596; 807. ^1^H NMR (400 MHz, DMSO-*d*_6_) δ (ppm): 12.95 (s, 1H, NH), 10.22 (s, 2H, NH), 7.08-7.39 (m, 4H, Ar), 5.52 (s, 1H), 2.18 (s, 3H). ^13^C NMR (400 MHz, DMSO) δ 158.3, 157.1, 156.9, 155.6, 145.8, 132.6, 124.6, 123.6, 111.1, 86.5, 35.8, 10.6. Anal. Cal. for C_15_H_11_ClN_4_O_3_. C: 54.48%, H: 3.35 %, N: 16.94%. Found: C: 54.49%, H: 3.56 %, N: 16.91%.

**4-(2-hydroxyphenyl)-3-methyl-6,8-dihydropyrazolo[4',3':5,6]pyrano[2,3-d]pyrimidine-5,7(1H,4H)-dione (*7c*):** White solid; m.p.: 229-231°C; FT-IR (KBr) (ν_max_/cm^-1^): 3435, 3121, 2922, 1642, 1437; 1216. ^1^H NMR (400 MHz, DMSO-*d*_6_) δ (ppm): 12.79 (s, 1H, NH), 11.98 (s, 1H, NH), 11.78 (s, 1H, NH), 10.15 (s, 1H, OH), 7.27-7.14 (m, 4H, Ar), 4.72 (s, 1H), 2.13 (s, 3H). ^13^C NMR (400 MHz, DMSO) δ 166.7, 160.8, 160.2, 134.7, 134.6, 134.2, 127.6, 124.7, 124.2, 119.1, 106.7, 104.7, 98.9, 109.1, 101.4, 87.8, 34.2, 16.2. Anal. Cal. for C_15_H_12_ClN_4_O_4_. C: 57.69%, H: 3.87 %, N: 17.94. Found: C: 57.49%, H: 3.89 %, N: 18.23%.

**4-(2-hydroxy-3-methoxyphenyl)-3-methyl-6,8-dihydropyrazolo[4',3':5,6]pyrano[2,3-d]pyrimidine-5,7(1H,4H)-dione (*7d*):** White solid; m.p.: 176-180°C; FT-IR (KBr) (ν_max_/cm^-1^): 3315, 3001, 2945, 2344, 1663, 1432; 1212. ^1^H NMR (400 MHz, DMSO-*d*_6_) δ (ppm): 12.00 (s, 1H, NH), 11.17 (s, 1H, NH), 11.30 (s, 1H, NH), 10.99 (s, 1H, OH), 7.04-7.16 (m, 3H), 4.70 (s, 1H), 3.84 (s, 3H, OMe), 1.91 (s, 3H). ^13^C NMR (400 MHz, DMSO) δ 179.0, 169.4, 155.8, 151.0, 148.9, 147.7, 139.02, 139. 01, 125.8, 122.0, 119.2, 112.3, 85.5, 56.1, 34.2, 18.9. Anal. Cal. for C_16_H_14_N_4_O_5_. C: 56.14%, H: 4.14 %, N: 16.37. Found: C: 56.23%, H: 4.82%, N: 16.24%. MS (m/z): calcd: 342.1, Obs. Mass: 342.1.

**4-(4-methoxyphenyl)-3-methyl-6,8-dihydropyrazolo[4',3':5,6]pyrano[2,3-d]pyrimidine-5,7(1H,4H)-dione (*7e*):** Yellow solid; m.p.: 229-231°C; FT-IR (KBr) (ν_max_/cm^-1^): 3373, 3121, 2344, 1687, 1454; 1223. ^1^H NMR (400 MHz, DMSO-*d*_6_) δ (ppm): 11.32 (s, 1H, NH), 11.19 (s, 1H, NH), 7.02-7.05 (d, *J*=12 Hz, 2H), 7.05-7.08 (d, *J*=12 Hz, 2H), 5.02 (s, 1H), 3.82 (s, 3H, OMe), 2.19 (s, 3H). ^13^C NMR (400 MHz, DMSO) δ 157.7, 150.1, 148.2, 138.2, 136.1, 133.3, 130.8, 129.7, 128.6, 126.7, 88.1, 66.3, 36.2, 15.3. Anal. Cal. for C_16_H_14_N_4_O_4_. C: 58.89%, H: 4.32 %, N: 17.17. Found: C: 58.23%, H: 4.85%, N: 16.97%.

**4-(3-chlorophenyl)-3-methyl-6,8-dihydropyrazolo[4',3':5,6]pyrano[2,3-d]pyrimidine-5,7(1H,4H)-dione (*7f*):** Pale Orange solid; m.p.: 299-301°C; FT-IR (KBr) (ν_max_/cm^-1^): 3438, 2923, 1624, 1595, 860. ^1^H NMR (400 MHz, DMSO-*d*_6_) δ (ppm): 12.82 (s, 1H, NH), 10.85 (s, 2H, NH), 7.23 (s, 1H), 6.98-7.14 (m, 3H), 5.82 (s, 1H), 2.14 (s, 3H). ^13^C NMR (400 MHz, DMSO) δ 165.2, 160.8, 150.0, 133.2, 132.1, 131.3, 131.2, 127.9, 126.5, 124.3, 88.8, 109.1, 29.3, 10.8. Anal. Cal. For C_15_H_11_ClN_4_O_3_. C: 54.48%, H: 3.35 %, N: 16.94. Found: C: 54.23%, H: 3.87%, N: 16.99%.

**3-methyl-4-(3-nitrophenyl)-6,8-dihydropyrazolo[4',3':5,6]pyrano[2,3-d]pyrimidine-5,7(1H,4H)-dione (*7g*):** White solid; m.p.: 265-267°C; FT-IR (KBr) (ν_max_/cm^-1^): 3597, 3468, 3030, 2920, 1700, 1586; 1478, 1356, 1300, 859, 810. ^1^H NMR (400 MHz, DMSO-*d*_6_) δ (ppm): 13.44 (s, 1H, NH), 10.27 (s, 2H, NH), 8.02-8.05 (m, 1H), 7.86 (s, 1H), 7.48-7.58 (m, 2H), 5.54 (s, 1H), 2.27 (s, 3H). ^13^C NMR (400 MHz, DMSO) δ 174.4, 159.4, 154.9, 149.3, 147.7, 144.5, 144.1, 137.5, 134.1, 130.5, 121.9, 110.4, 31.4, 10.8. Anal. Cal. For C_15_H_11_N_5_O_5_. C: 52.79%, H: 3.25 %, N: 20.52. Found: C: 52.71%, H: 3.35%, N: 19.99%.

**3-methyl-4-phenyl-6,8-dihydropyrazolo[4',3':5,6]pyrano[2,3-d]pyrimidine-5,7(1H,4H)-dione (*7h*):** White solid; m.p.: 203-206°C; FT-IR (KBr) (ν_max_/cm^-1^): 3421, 2889, 2762, 1678, 1587, 1469; 1309, 780. ^1^H NMR (400 MHz, DMSO-*d*_6_) δ (ppm): 13.15 (s, 1H, NH), 10.20 (s, 2H, NH), 7.00-7.23 (m, 5H, Ar), 5.44 (s, 1H), 2.23 (s, 3H). ^13^C NMR (400 MHz, DMSO) δ 164.9, 160.9, 150.0, 143.2, 142.7, 127.0, 126.0, 125.8, 105.0, 92.6, 90.9, 30.2, 9.3. Anal. Cal. For C_15_H_12_N_4_O_3._ C: 60.81%, H: 4.08 %, N: 18.91. Found: C: 69.62%, H: 4.35%, N: 18.98%.

**3-methyl-4-(p-tolyl)-6,8-dihydropyrazolo[4',3':5,6]pyrano[2,3-d]pyrimidine-5,7(1H,4H)-dione (*7i*):** Pale yellow solid; m.p.: 200-203°C; FT-IR (KBr) (ν_max_/cm^-1^): 3421, 3121, 3042, 1687; 1577, 1569, 1271. ^1^H NMR (400 MHz, DMSO-*d*_6_) δ (ppm): 13.14 (s, 1H, NH), 10.16 (s, 2H, NH), 7.09-7.11 (d, *J*= 8 Hz, 2H, Ar), 6.91-6.93 (d, *J*=8 Hz, 2H, Ar), 5.40 (s, 1H), 2.51 (s, 3H), 2.22 (s, 3H). ^13^C NMR (400 MHz, DMSO) δ 156.3, 151.6, 141.6, 136.4, 131.1, 127.5, 127.5, 126.5, 124.1, 117.8, 106.3, 31.2, 21.6, 10.6. Anal. Cal. For C_16_H_14_N_4_O_3._ C: 61.93%, H: 4.55%, N: 18.06. Found: C: 62.02%, H: 4.85%, N: 15.05%.

**3-methyl-4-(4-nitrophenyl)-6,8-dihydropyrazolo[4',3':5,6]pyrano[2,3-d]pyrimidine-5,7(1H,4H)-dione (*7j*):** White solid; m.p.: 225-228°C; FT-IR (KBr) (ν_max_/cm^-1^): 3147, 3056, 2900, 1693; 1590, 1517, 1469, 1351, 1273, 832. ^1^H NMR (400 MHz, DMSO-*d*_6_) δ (ppm): 11.48 (s, 1H, NH), 9.90 (s, 2H, NH), 7.59-7.61 (d, *J*= 8 Hz, 2H, Ar), 7.12-7.14 (d, *J*=8 Hz, 2H, Ar), 5.09 (s, 1H), 1.59 (s, 3H). ^13^C NMR (400 MHz, DMSO) δ 158.6, 151.1, 150.1, 131.2, 136.2, 133.4, 91.2, 31.6, 9.7. Anal. Cal. For C_15_H_11_N_5_O_5._ C: 52.7%, H: 3.25%, N: 20.52. Found: C: 52.02%, H: 3.43%, N: 20.98%.

**3-methyl-4-(m-tolyl)-6,8-dihydropyrazolo[4',3':5,6]pyrano[2,3-d]pyrimidine-5,7(1H,4H)-dione (*7k*):** White solid; m.p.: 242-244°C; FT-IR (KBr) (ν_max_/cm^-1^): 3467, 3187, 2915, 1702; 1692, 1474, 1321, 1167, 564, 545. ^1^H NMR (400 MHz, DMSO-*d*_6_) δ (ppm): 12.58 (s, 1H, NH), 10.20 (s, 2H, NH), 6.83-6.98 (m, 3H, Ar), 5.40 (s, 1H), 2.51 (s, 3H), 2.22 (s, 3H). ^13^C NMR (400 MHz, DMSO) δ 168.2, 160.8, 151.1, 142.2, 137.1, 128.2, 127.2, 126.5, 125.6, 124.3, 105.2, 91.9, 79.6, 30.8, 21.7, 10.6. Anal. Cal. For C_16_H_14_N_4_O_5._ Found: C: 56.03%, H: 4.19%, N: 16.66%. MS (m/z): calcd: 310.1, Obs. MS m/z: 310.2 (M+).

**4-(4-isopropylphenyl)-3-methyl-6,8-dihydropyrazolo[4',3':5,6]pyrano[2,3-d]pyrimidine-5,7(1H,4H)-dione (*7l*):** White solid; m.p.: 218-220°C; FT-IR (KBr) (ν_max_/cm^-1^): 3455, 3221, 2987, 2024, 1693, 1411; 1299. ^1^H NMR (400 MHz, DMSO-*d*_6_) δ (ppm): 12.87 (s, 1H, NH), 10.20 (s, 2H, NH), 6.96-6.98 (d, *J*= 8 Hz, 2H, Ar), 7.07-7.09 (d, *J*=8 Hz, 2H, Ar), 5.35 (s, 1H), 2.76-2.86 (hept, 1H), 2.30 (s, 3H), 2.30 (s, 3H), 1.15-1.19 (d, *J*= 16 Hz, 6H). ^13^C NMR (400 MHz, DMSO) δ 165.6, 155.2, 146.4, 142.4, 142.2, 134.9, 130.9, 130.2, 129.6, 109.2, 93.9, 33.3, 23.9, 23.4, 9.9. vAnal. Cal. For C_18_H_18_N_4_O_3._ C: 63.89%, H: 5.36%, N: 16.56. Found: C: 63.92%, H: 5.43%, N: 16.89%.

**4-(5-bromo-2-hydroxyphenyl)-3-methyl-4,8-dihydropyrazolo[4',3':5,6]pyrano[2,3 d]pyrimidine-5,7(1H,6H)-dione (*7m*):** Yellow solid; m.p.: 260-263°C; FT-IR (KBr) (ν_max_/cm^-1^): 3165, 3003, 2890, 1593, 1556, 1129, 873, 799, 496. ^1^H NMR (400 MHz, DMSO-*d*_6_) δ (ppm): 13.07 (s, 1H, OH), 10.35 (s, NH), 10.22 (s, 2H, NH), 7.39-7.92 (m, 2H, Ar), 7.13 (s, 1H, Ar), 5.56 (s, 1H), 1.07 (s, 3H). ^13^C NMR (400 MHz, DMSO) δ 163.6, 158.7, 142.2, 152.6, 149.0, 139.6, 129.6, 127.5, 115.2, 107.7, 103.8, 87.5, 54.3, 24.7, 16.6. Anal. Cal. For C_15_H_11_BrN_4_O_4._ Found: C: 46.11%, H: 3.03%, N: 20.57%. MS (m/z): calcd: 390.0, Obs. MS m/z: 390.9 (M+).

**4-(2-hydroxy-4-methoxyphenyl)-3-methyl-7-thioxo4,6,7,8tetrahydropyrazolo[4',3':5,6] pyrano[2,3-d]pyrimidin-5(1H)-one (*7n*):** Light yellow powder; m.p.: 252-254°C; ; FT-IR (KBr) (ν_max_/cm^-1^): 3376, 2927, 1741, 1607, 1400; 1296, 1032. ^1^H NMR (400 MHz, DMSO-*d*_6_) δ (ppm): 13.30 (s, 1H, NH), 12.31 (s, 1H, OH), 11.46 (s, 2H, NH), 6.06-6.95 (m, 3H), 5.62 (s, 1H), 3.77 (s, 3H, OMe), 2.09 (s, 3H). ^13^C NMR (400 MHz, DMSO) δ 179.0, 169.4, 155.8, 151.0, 148.9, 147.7, 139.02, 139. 01, 125.8, 122.0, 119.2, 112.3, 85.5, 56.1, 34.2, 18.9. ^13^C NMR (400 MHz, DMSO) δ 174.7, 167.6, 149.6, 133.0, 129.9, 127.1, 125.0, 124.9, 123.2, 110.8, 109.6, 69.3, 68.6, 34.3, 17.7. Anal. Cal. For C_16_H_14_N_4_O_4_S_._ Found: C: 53.55%, H: 3.53%, N: 15.59%. MS (m/z): calcd: 358.1, Obs. MS m/z: 358.1. (M+).


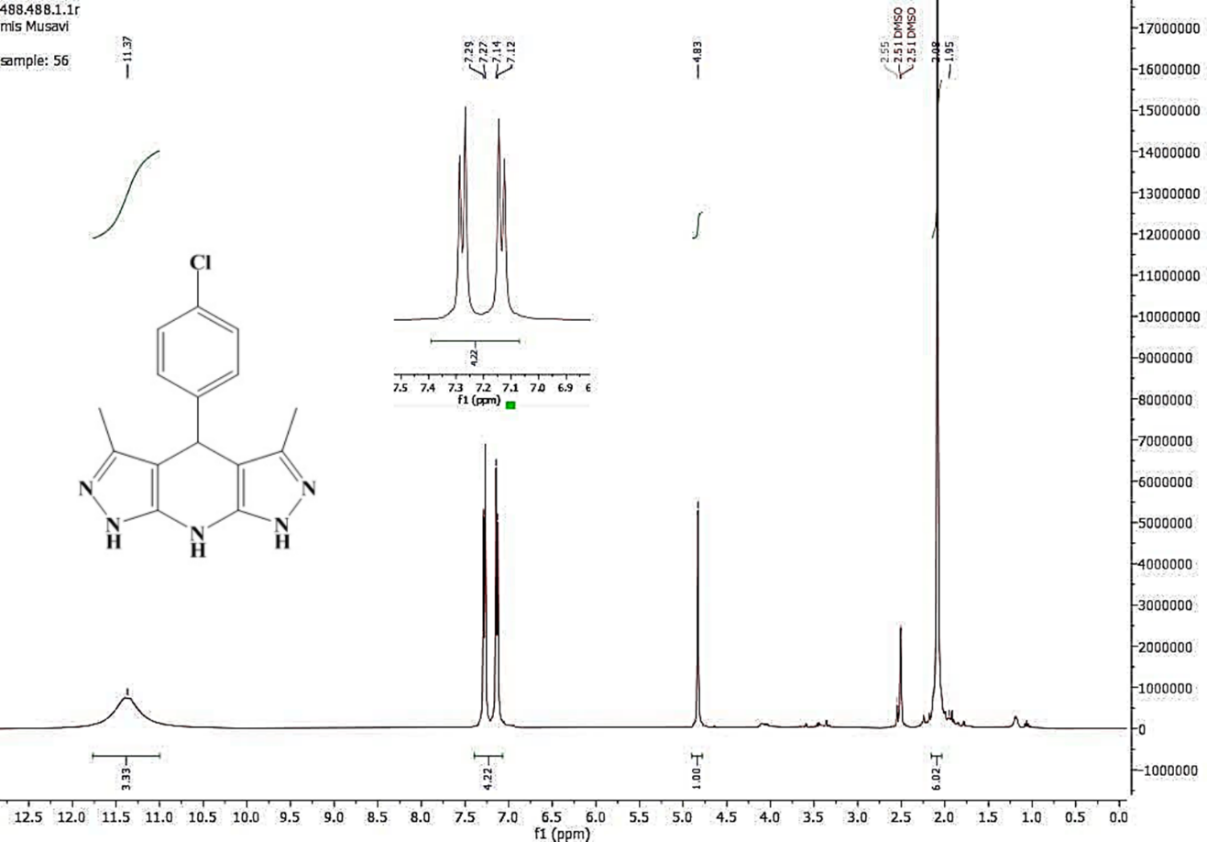


**FIGURE S4.** ^1^H NMR spectrum of Compound **5a.**


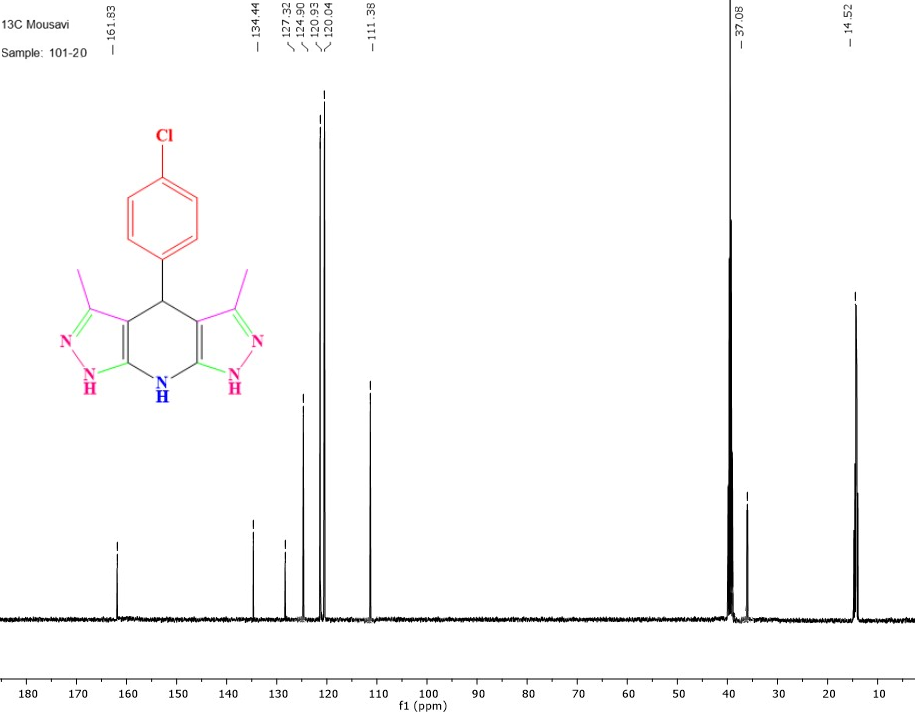


**FIGURE S5.** ^13^C NMR spectrum of Compound **5a.**


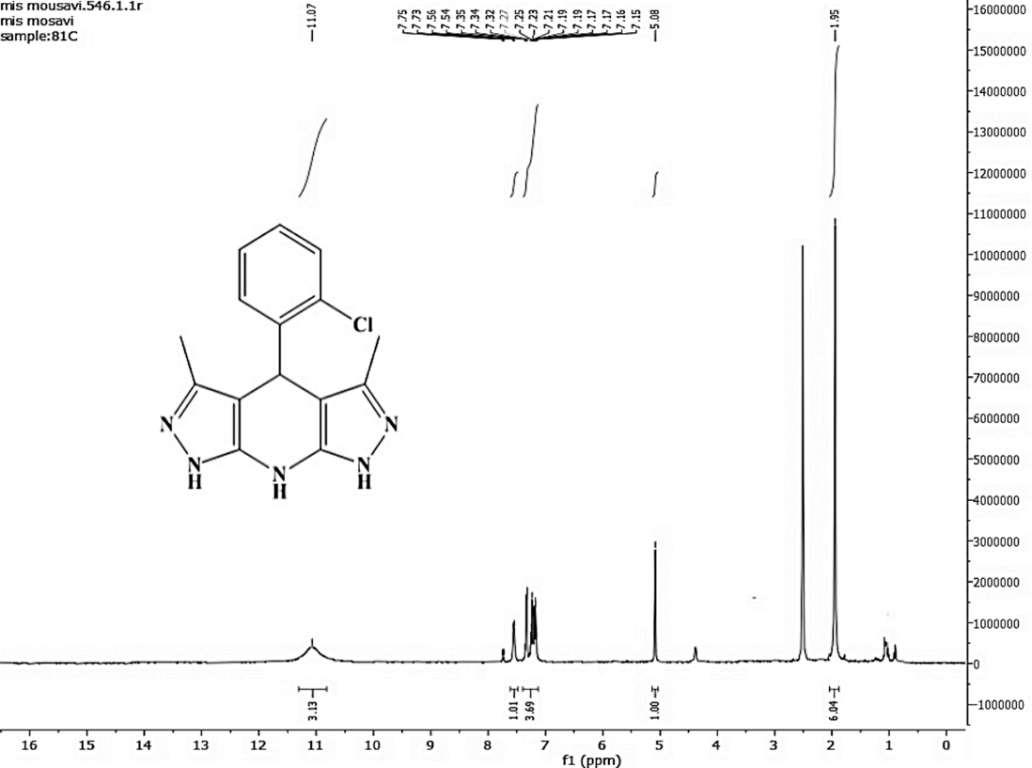


**FIGURE S6.** ^1^H NMR spectrum of Compound **5b.**


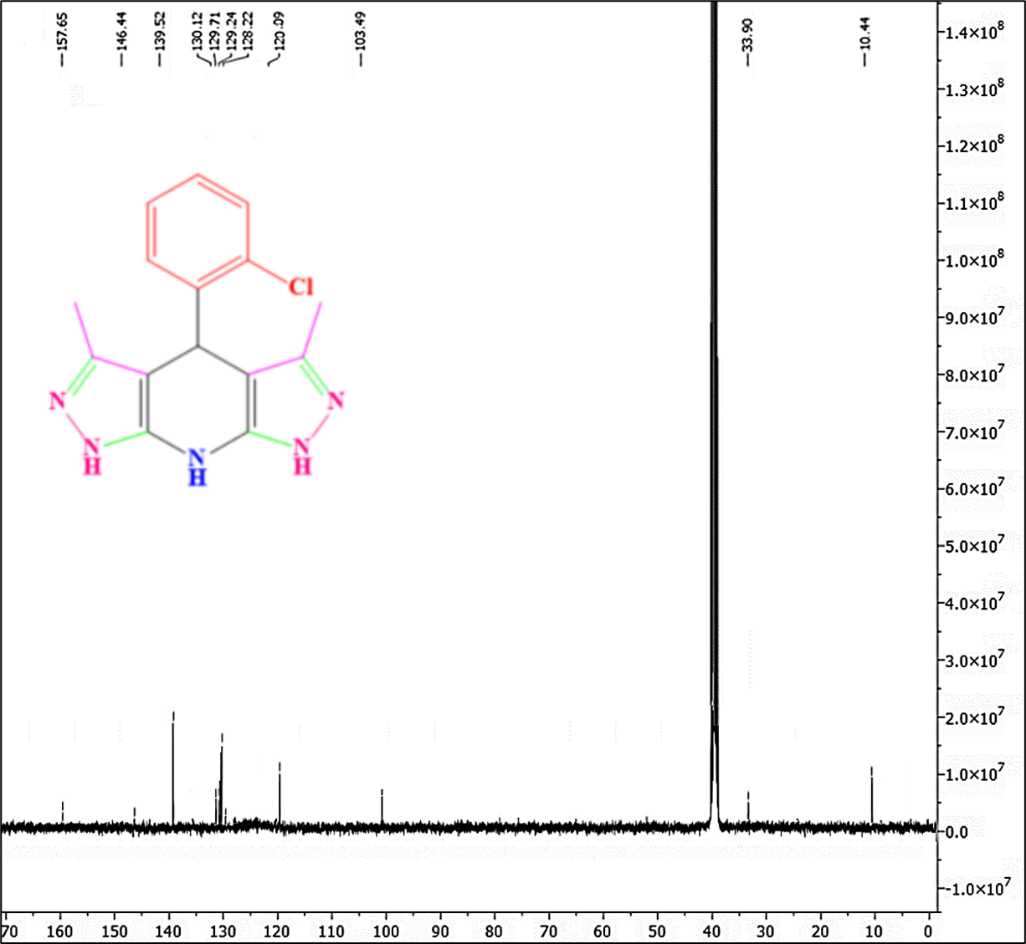


**FIGURE S7.** ^13^C NMR spectrum of Compound **5b.**


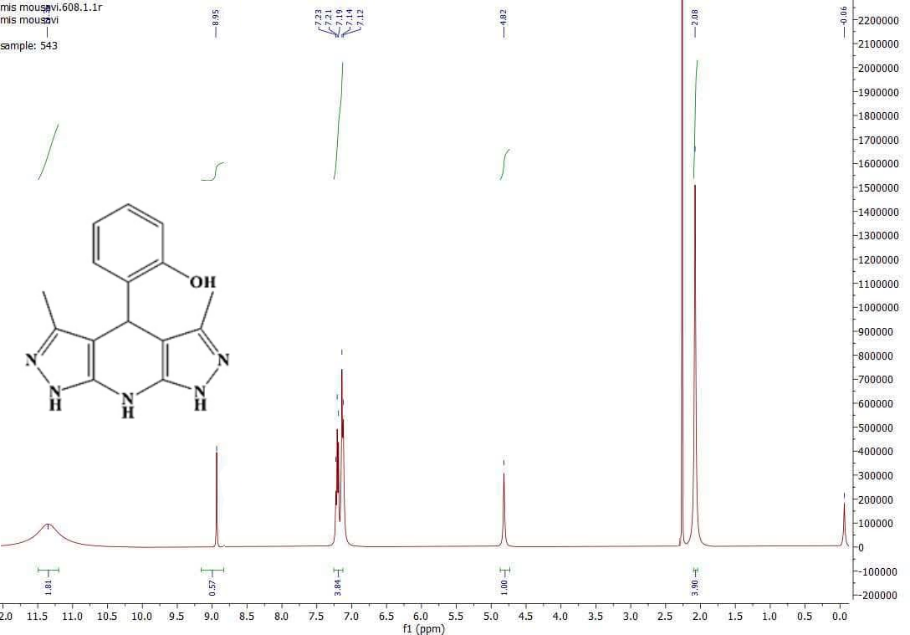


**FIGURE S8.** ^1^H NMR spectrum of Compound **5c.**

**
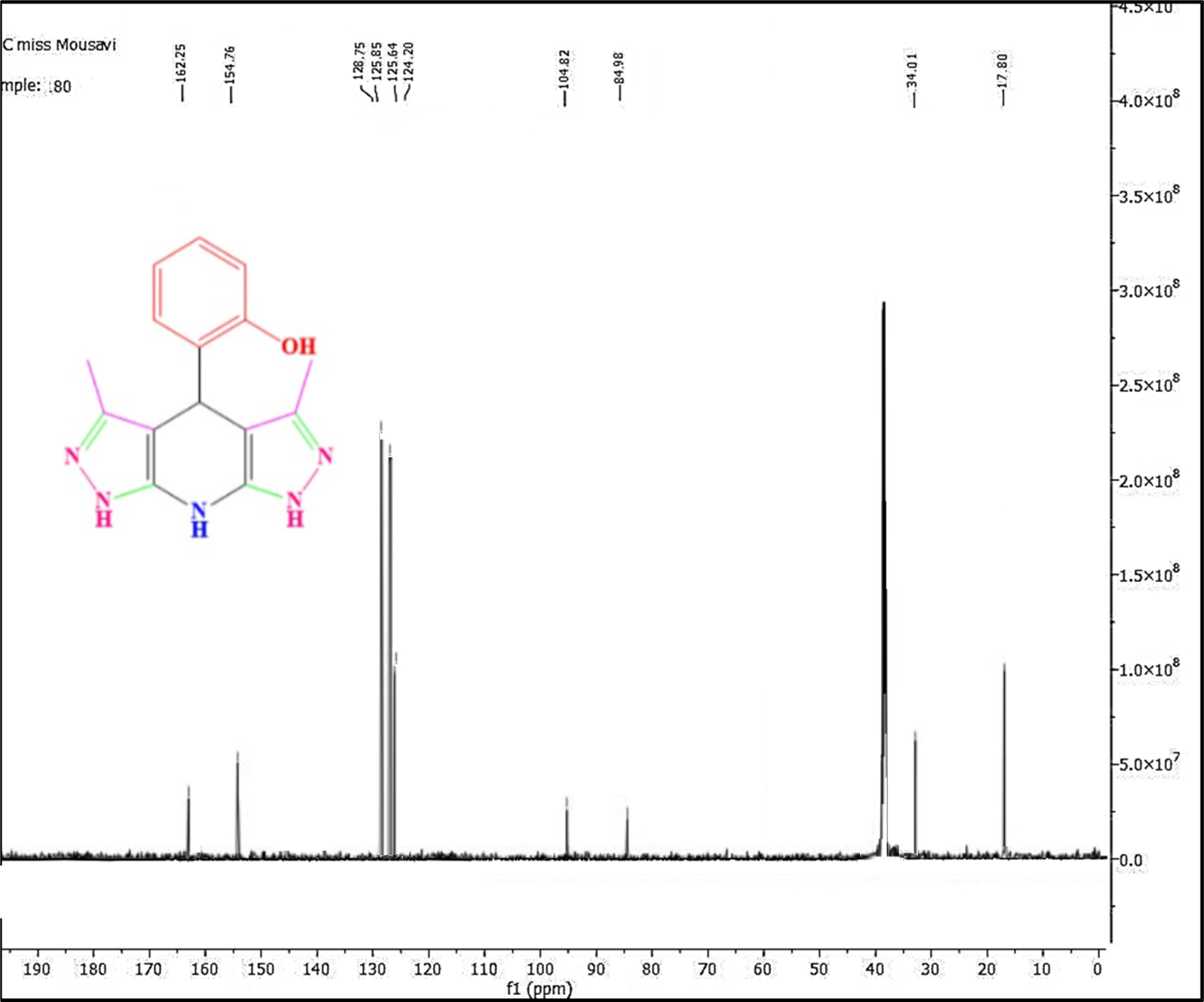
**

**FIGURE S9.** ^13^C NMR spectrum of Compound **5c.**

*s*
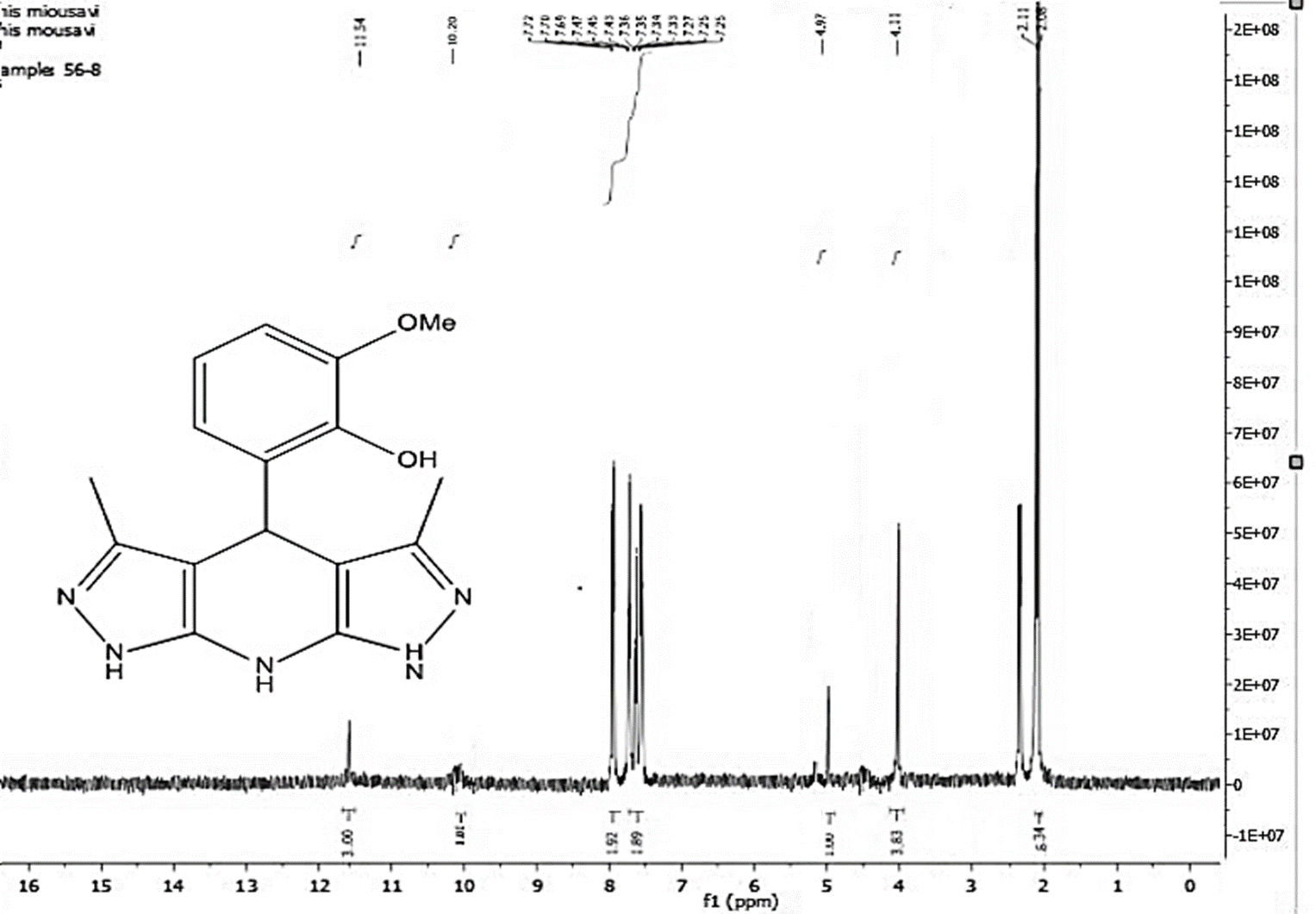


**FIGURE S10.** ^1^H NMR spectrum of Compound **5d.**


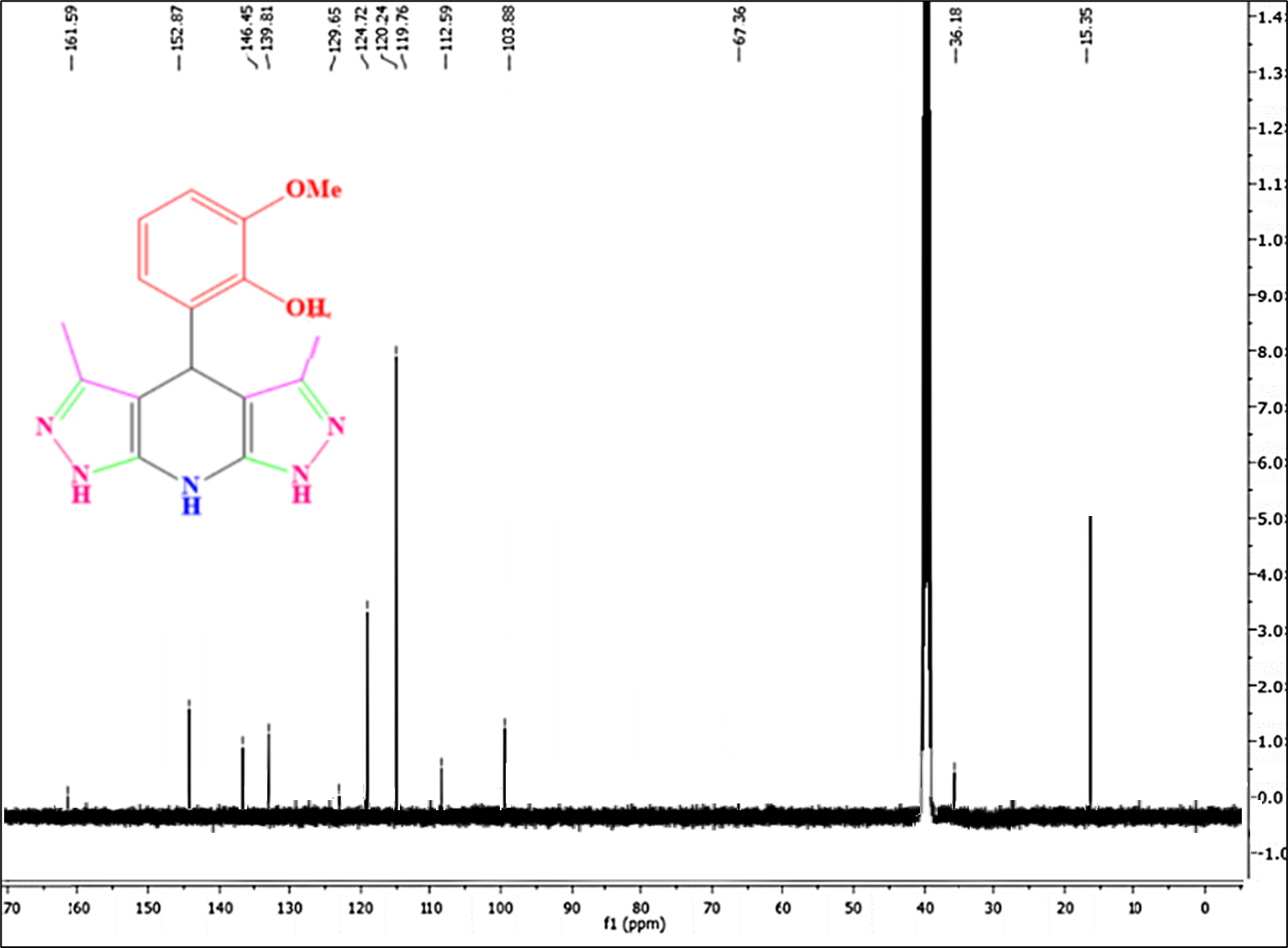


**FIGURE S11.** ^13^C NMR spectrum of Compound **5d.**


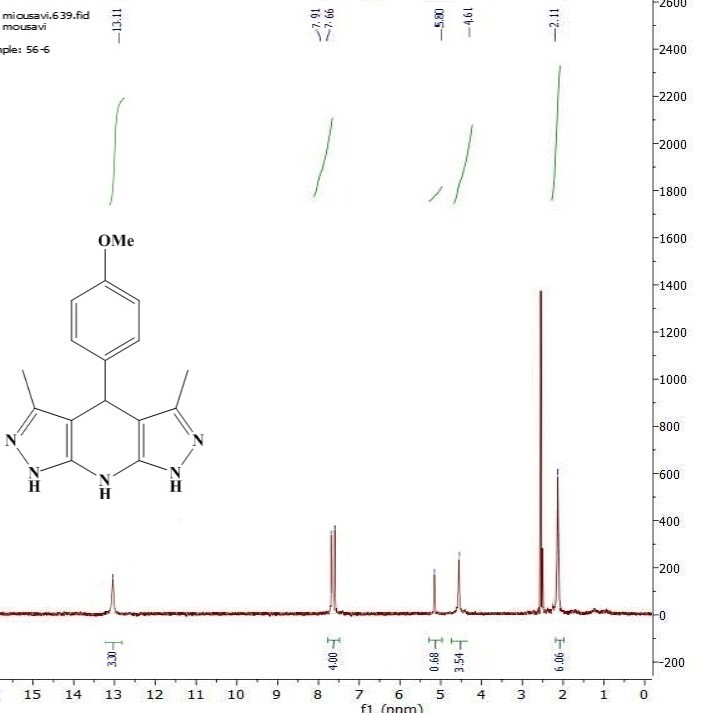


**FIGURE S12.** ^1^H NMR spectrum of Compound **5e.**


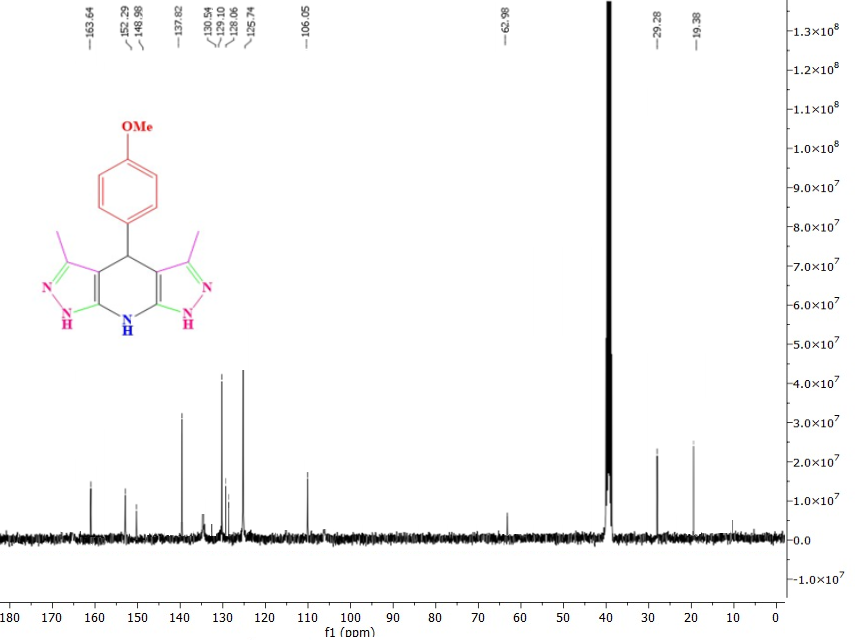


**FIGURE S13.** ^13^C NMR spectrum of Compound **5e.**


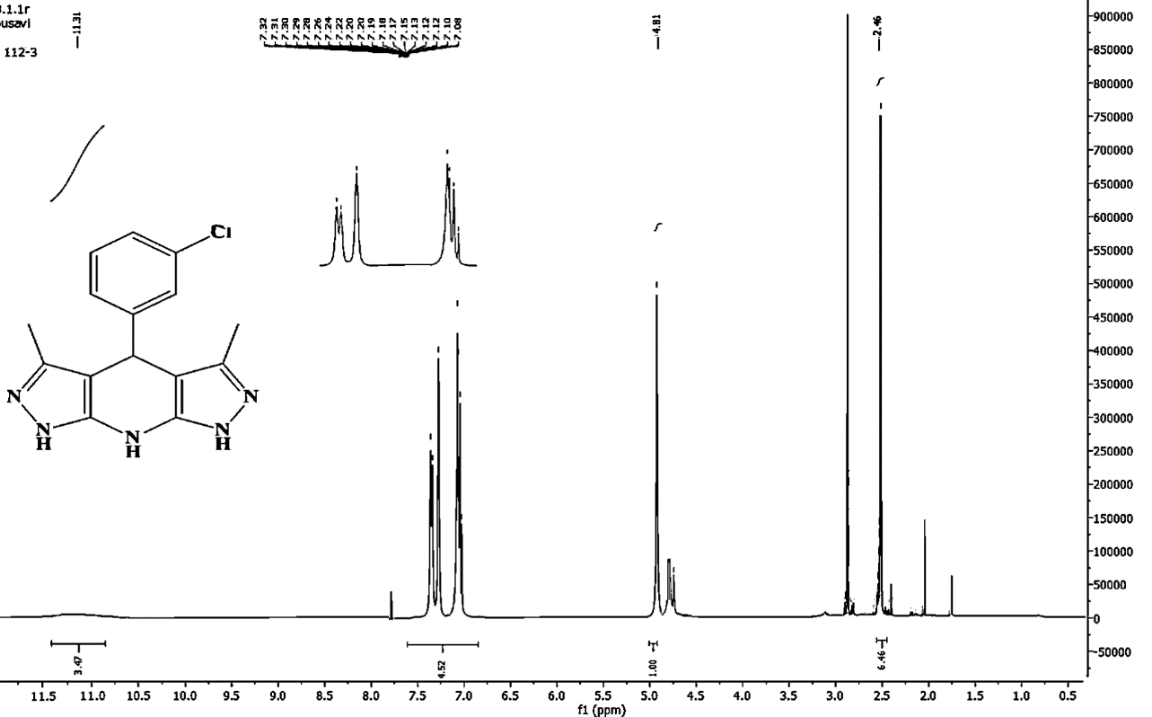


**FIGURE S14.** ^1^H NMR spectrum of Compound **5f.**

**
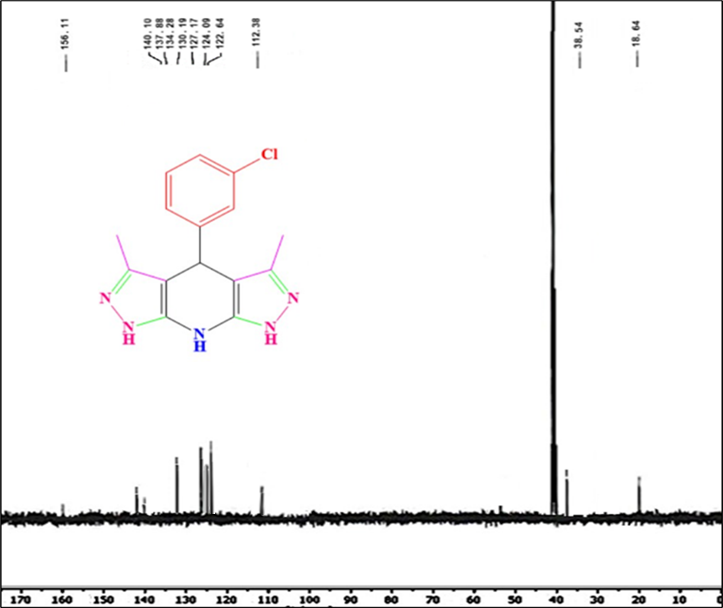
**

**FIGURE S15.** ^13^C NMR spectrum of Compound **5f.**

*
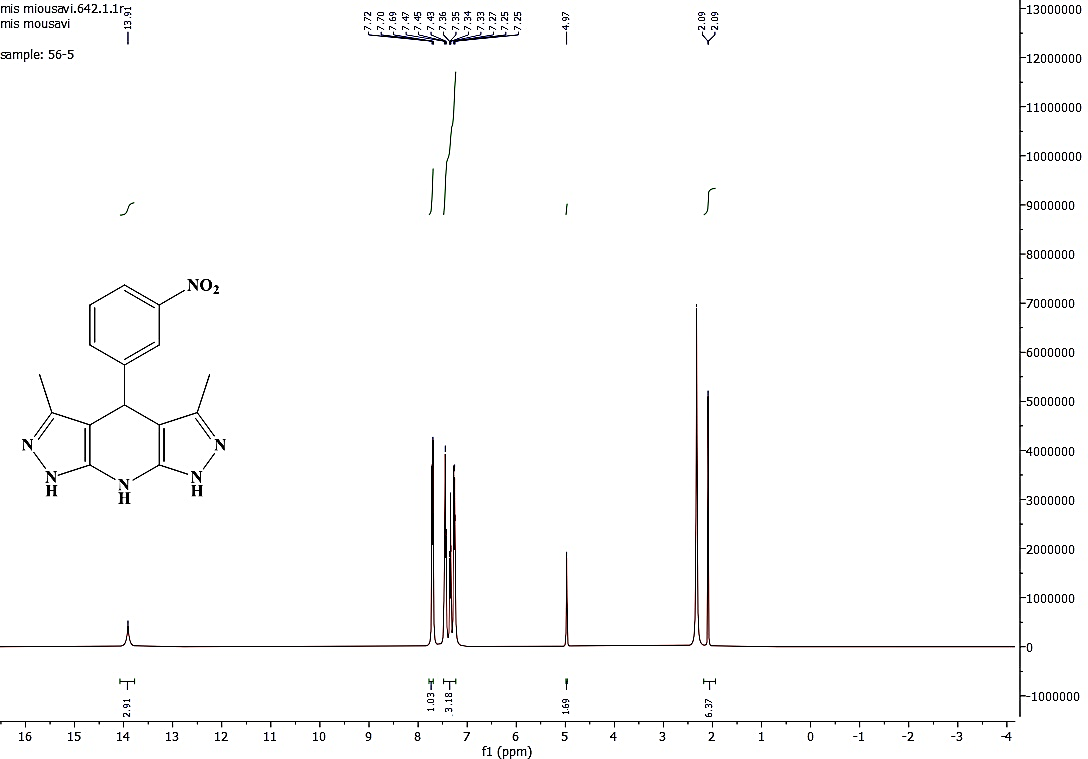
*

**FIGURE S16.** ^1^H NMR spectrum of Compound **5g.**


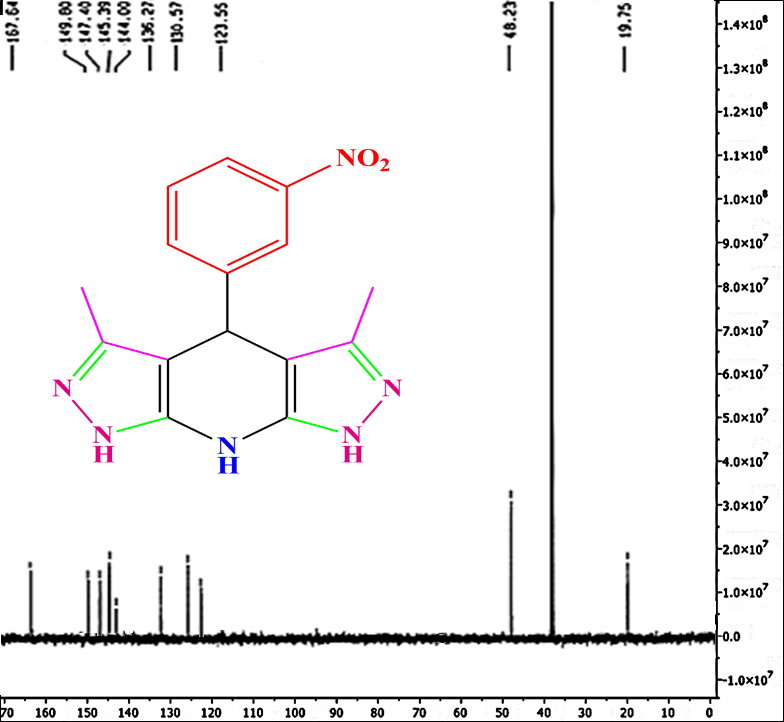


**FIGURE S17.** ^13^C NMR spectrum of Compound **5g.**


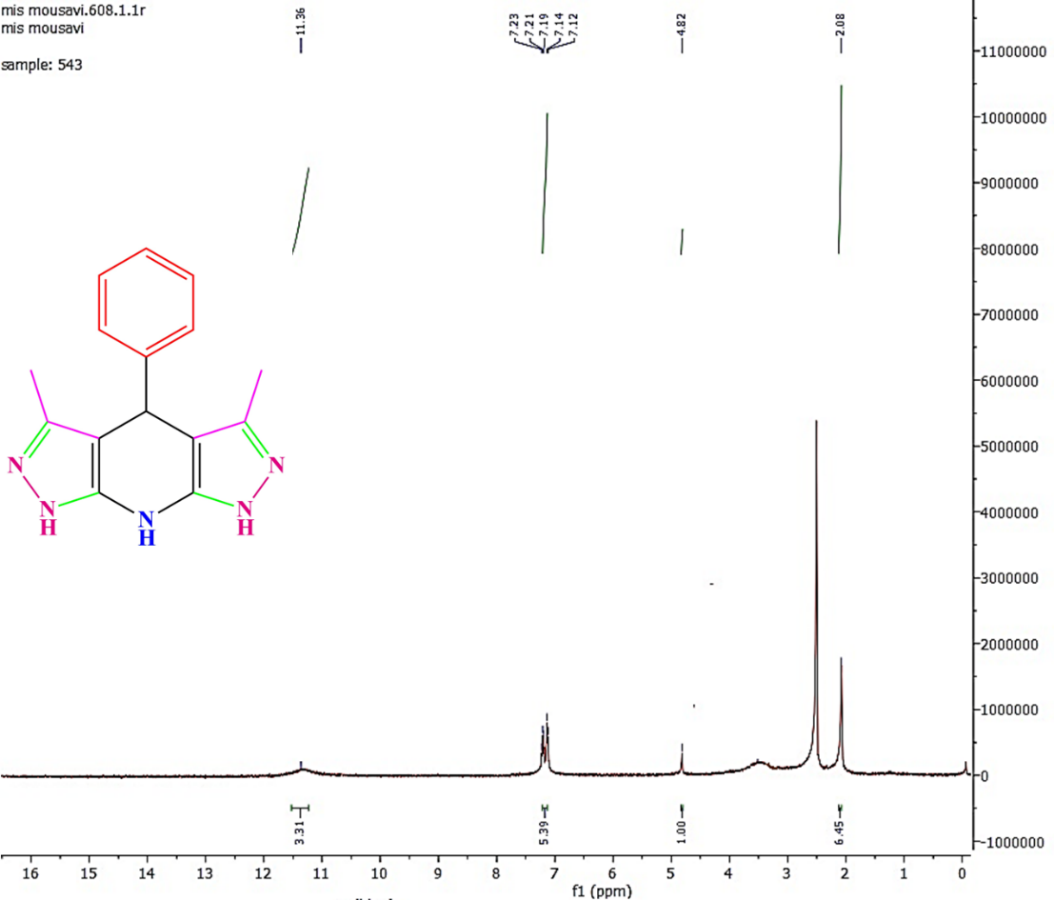


**FIGURE S18.** ^1^H NMR spectrum of Compound **5h.**


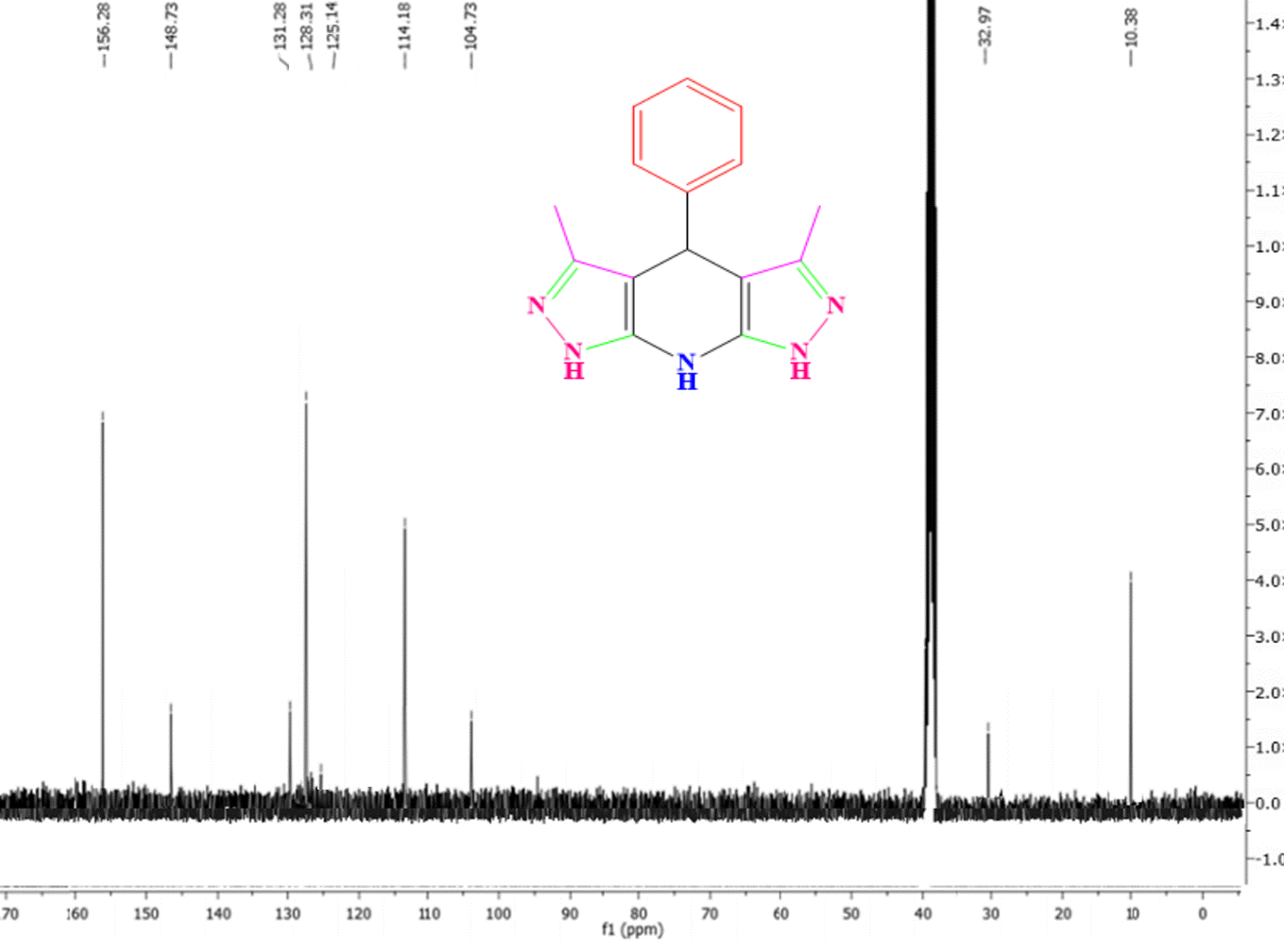


**FIGURE S17.** ^13^C NMR spectrum of Compound **5h.**

*
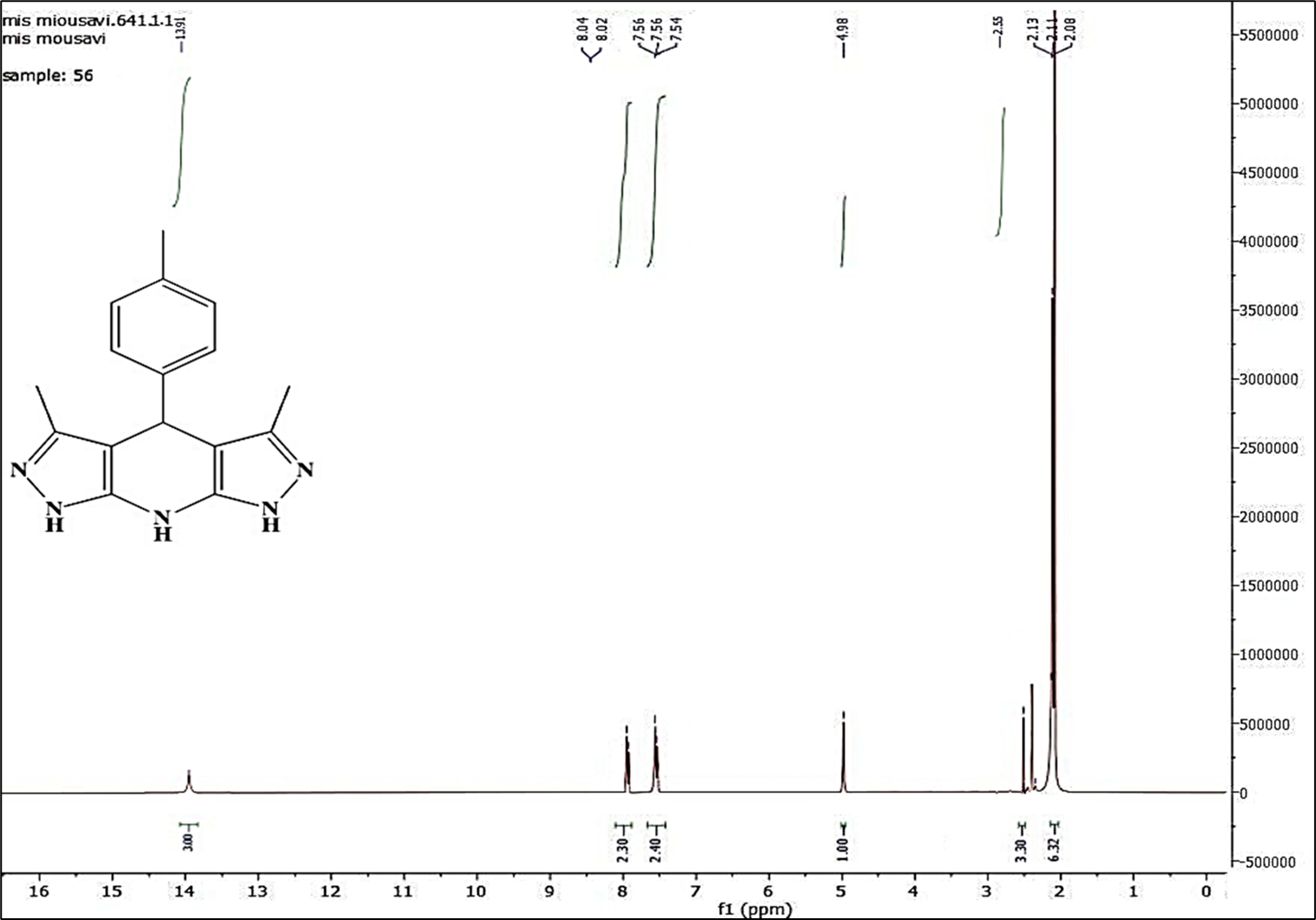
*

**FIGURE S20.** ^1^H NMR spectrum of Compound **5i.**


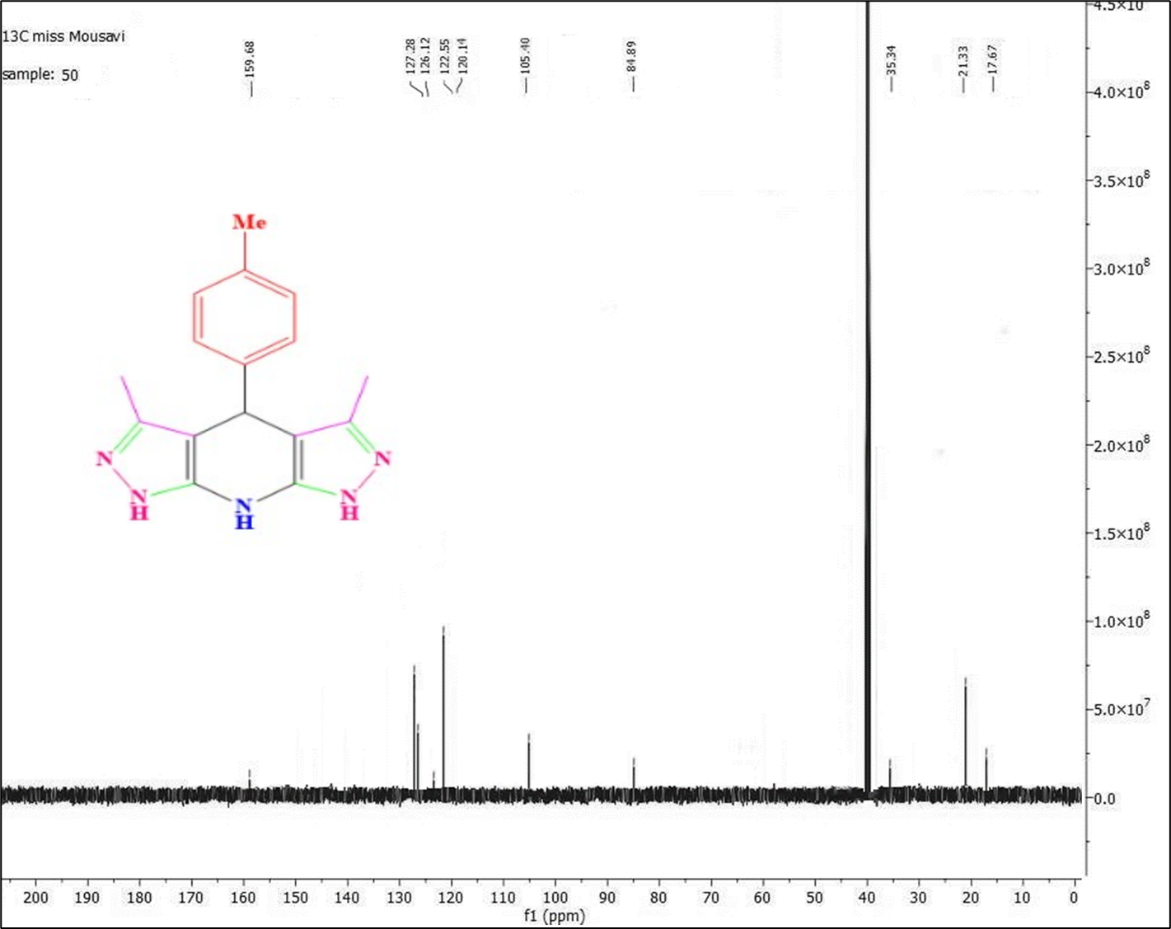


**FIGURE S21.** ^13^C NMR spectrum of Compound **5i.**


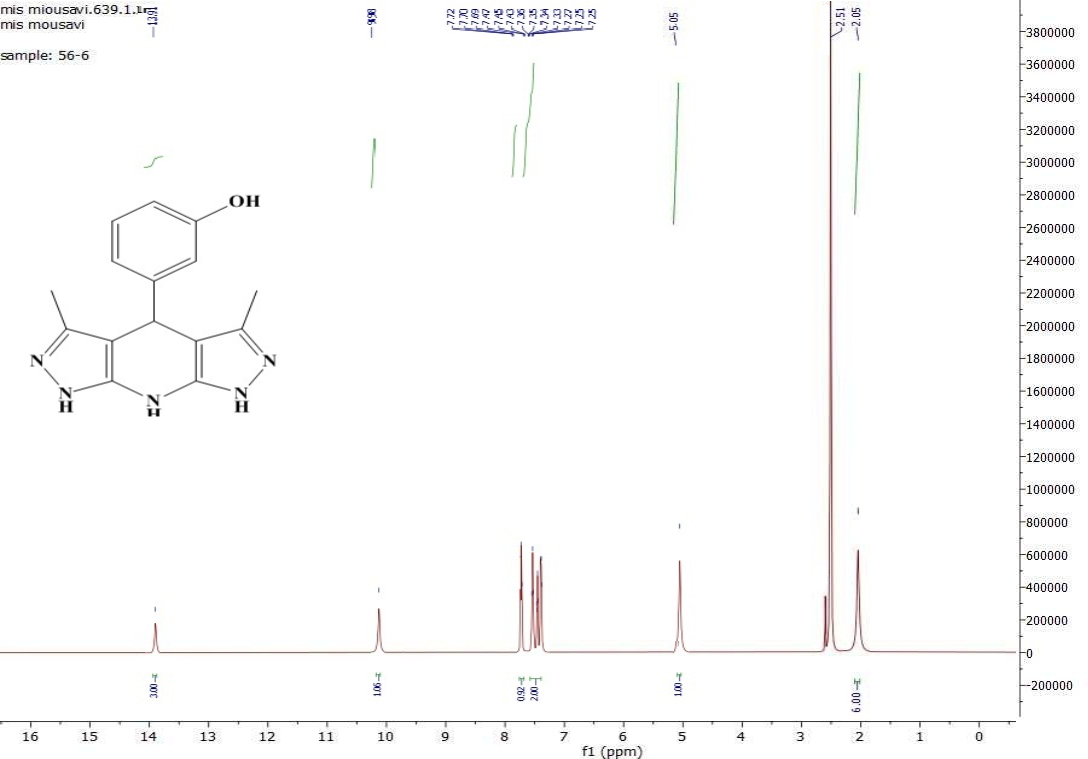


**FIGURE S22.** ^1^H NMR spectrum of Compound **5j.**

**
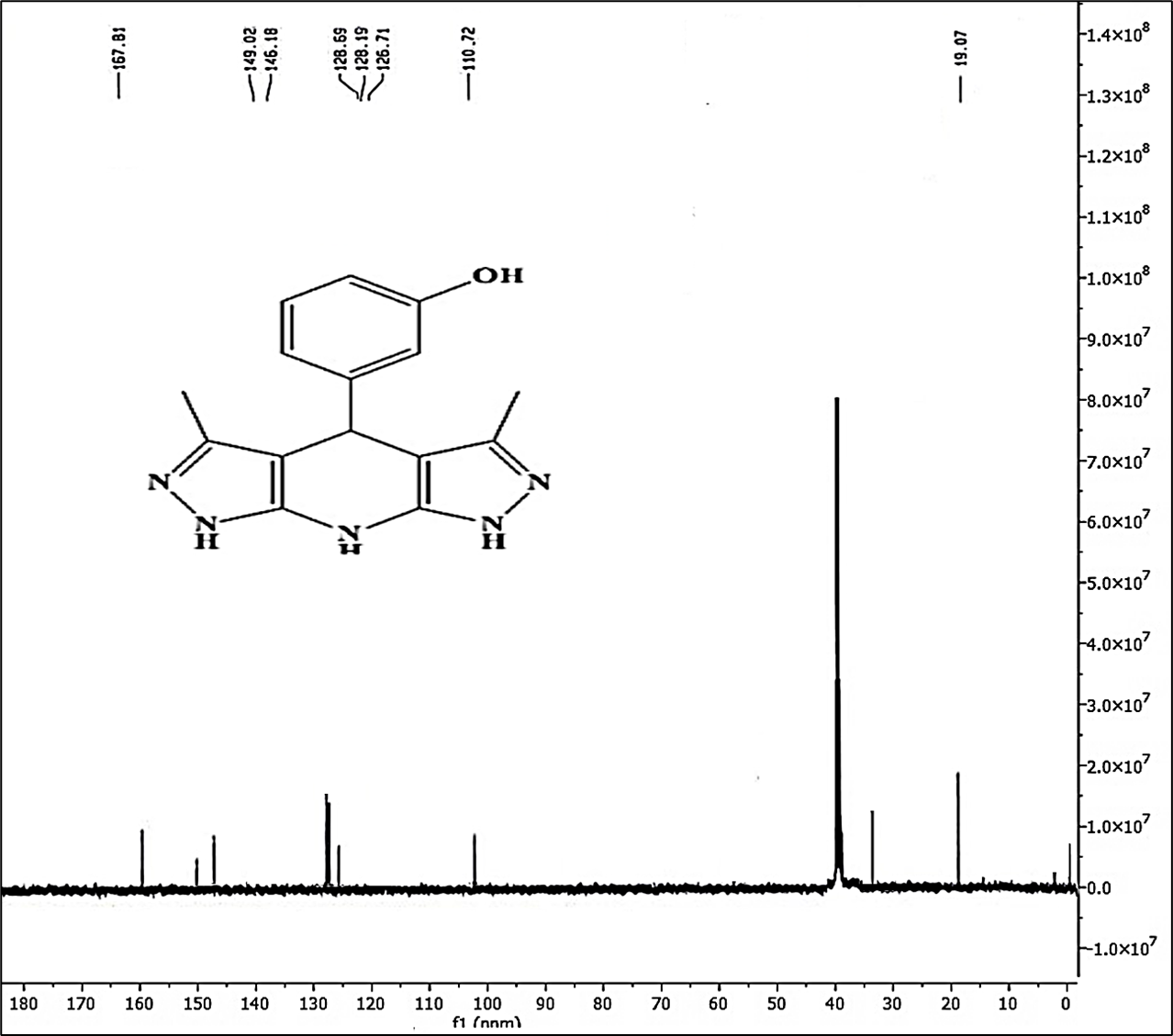
**

**FIGURE S23.** ^13^C NMR spectrum of Compound **5j.**


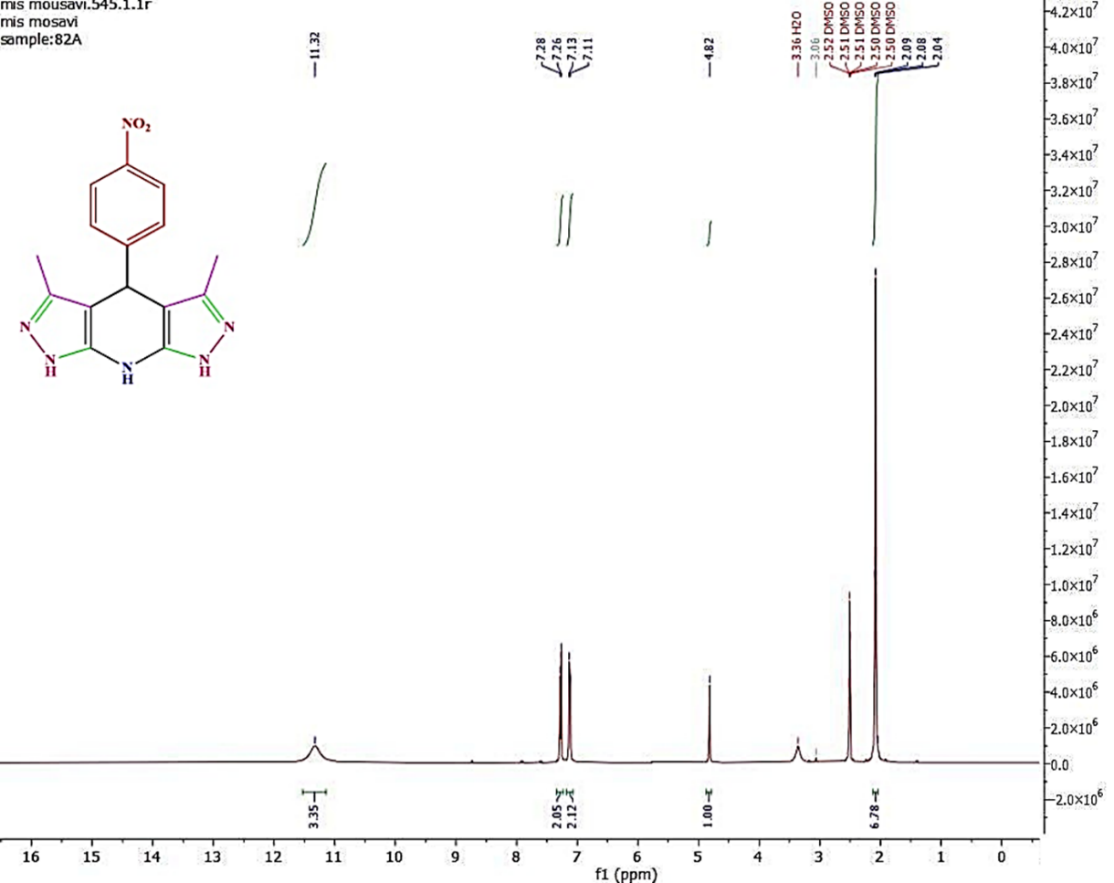


**FIGURE S24.** ^1^H NMR spectrum of Compound **5k.**


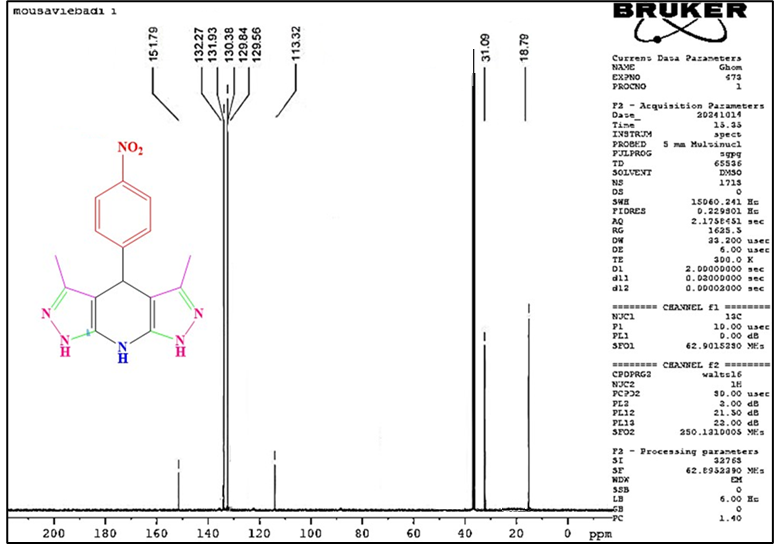


**FIGURE S25.** ^13^C NMR spectrum of Compound **5k.**

**
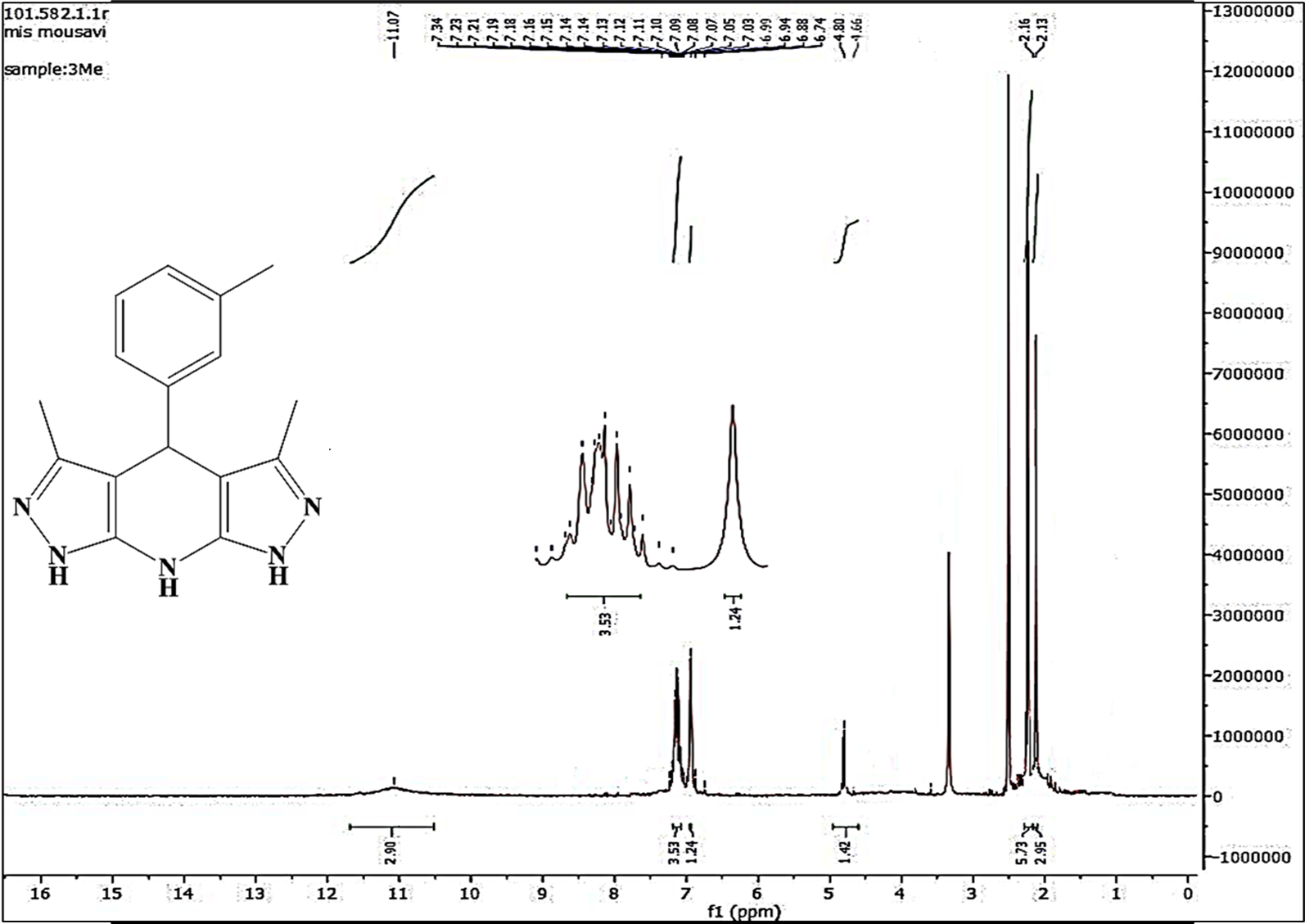
**

**FIGURE S26.** ^1^H NMR spectrum of Compound **5l.**

**
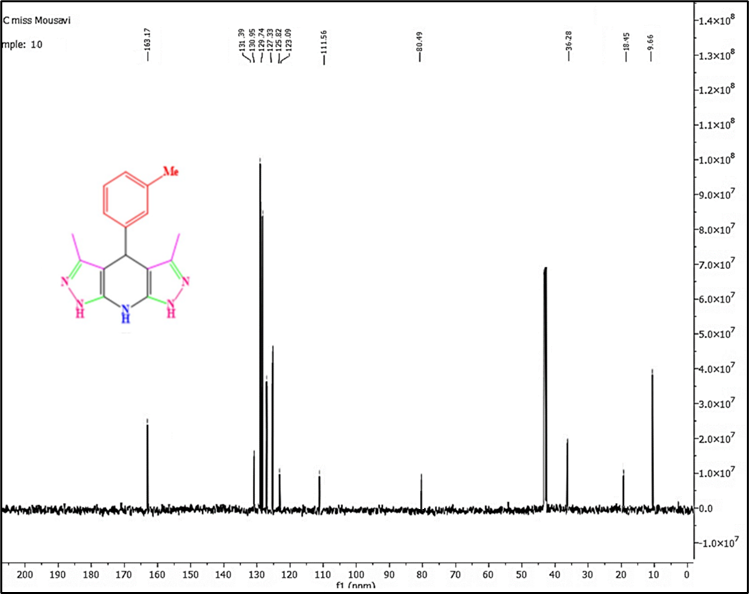
**

**FIGURE S27.** ^13^C NMR spectrum of Compound **5l.**

*
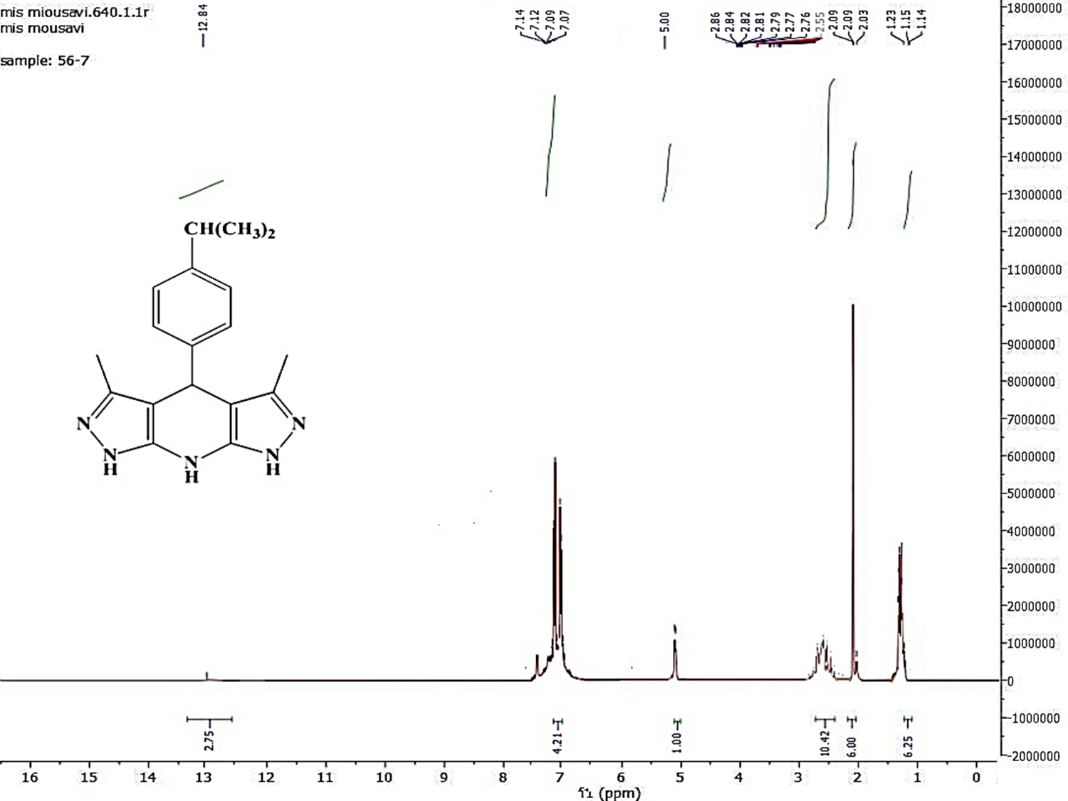
*

**FIGURE S28.** ^1^H NMR spectrum of Compound **5m.**

**
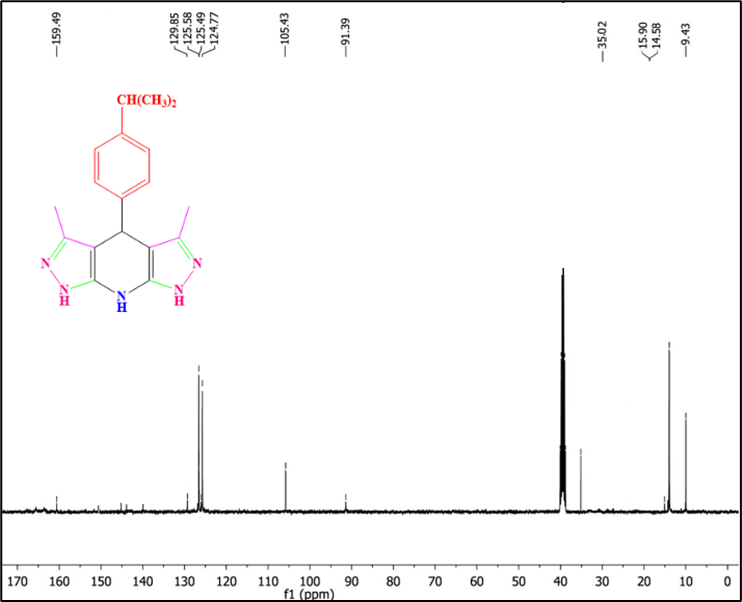
**

**FIGURE S29.** ^13^C NMR spectrum of Compound **5m**

**
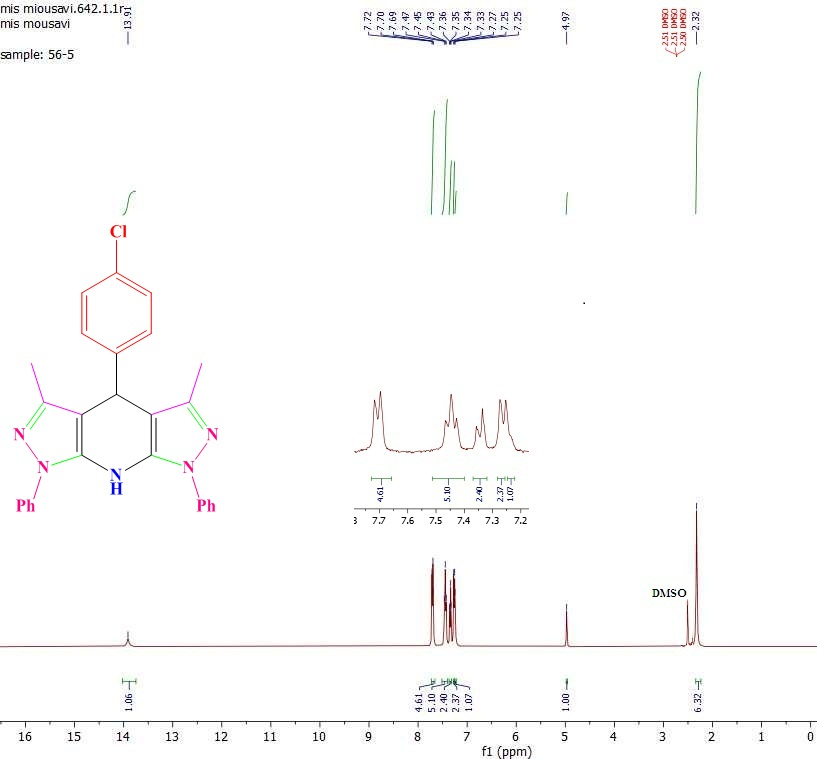
**

**FIGURE S30.** ^1^H NMR spectrum of Compound **5n.**

**
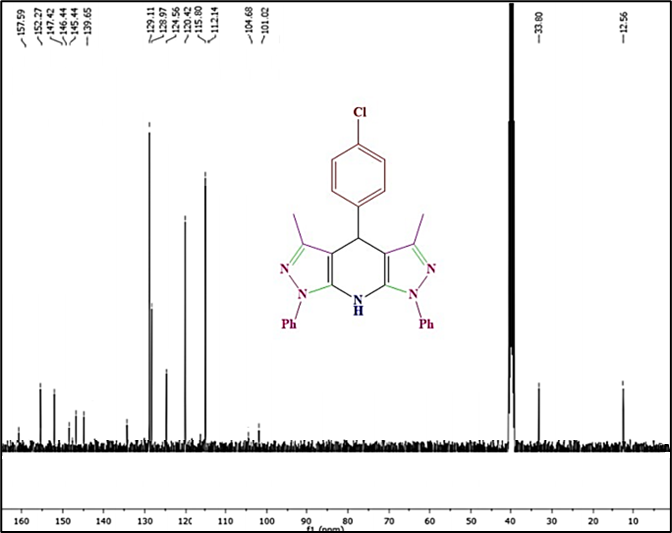
**

**FIGURE S31.** ^13^C NMR spectrum of Compound **5n.**

*
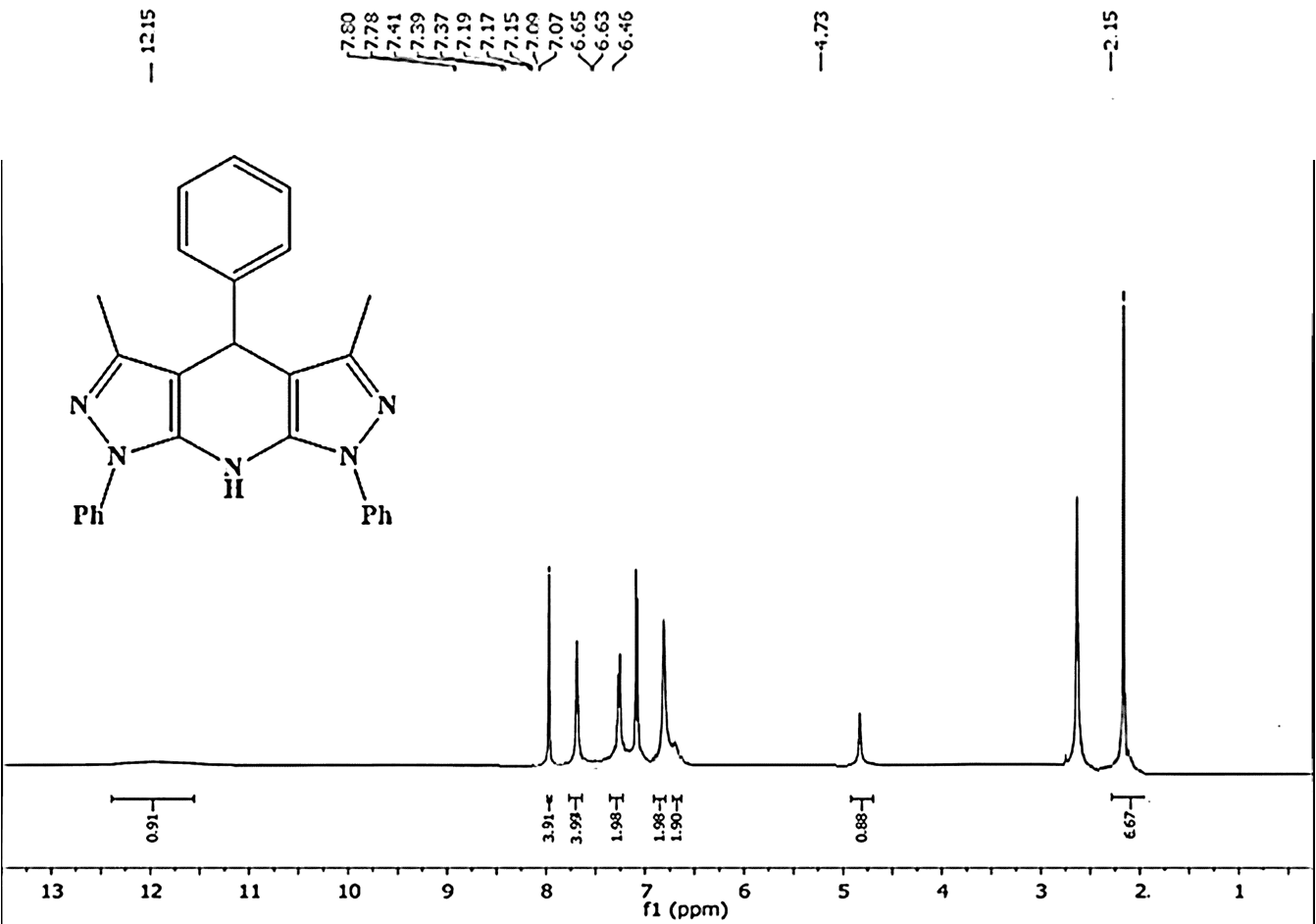
*

**FIGURE S32.** ^1^H NMR spectrum of Compound **5o.**

**
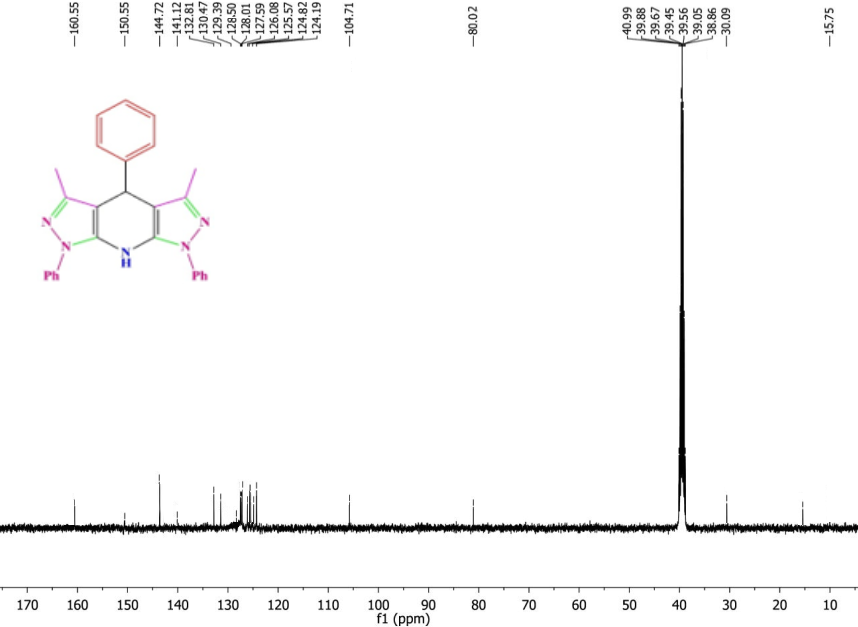
**

**FIGURE S33.** ^13^C NMR spectrum of Compound **5o.**

*
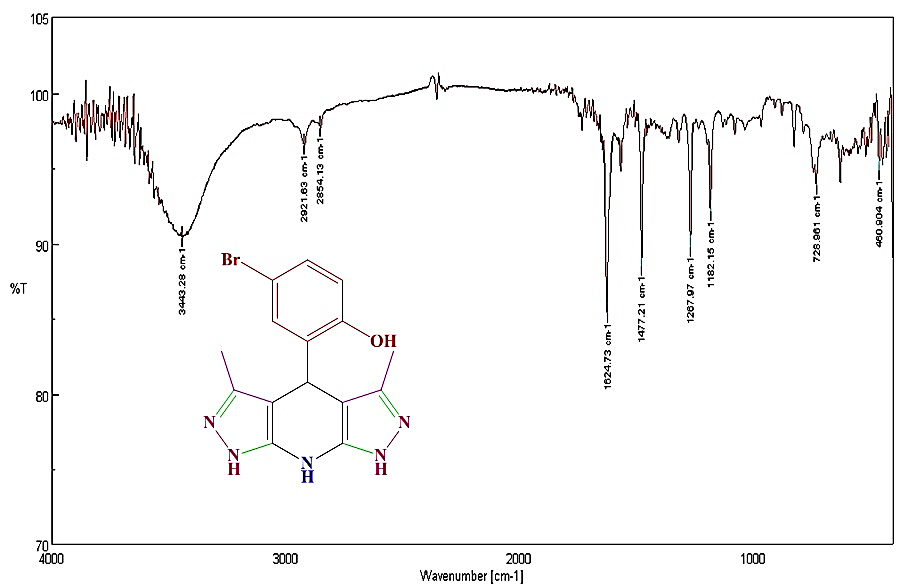
*

**FIGURE S34.** FT-IR spectrum of Compound **5p**.

***
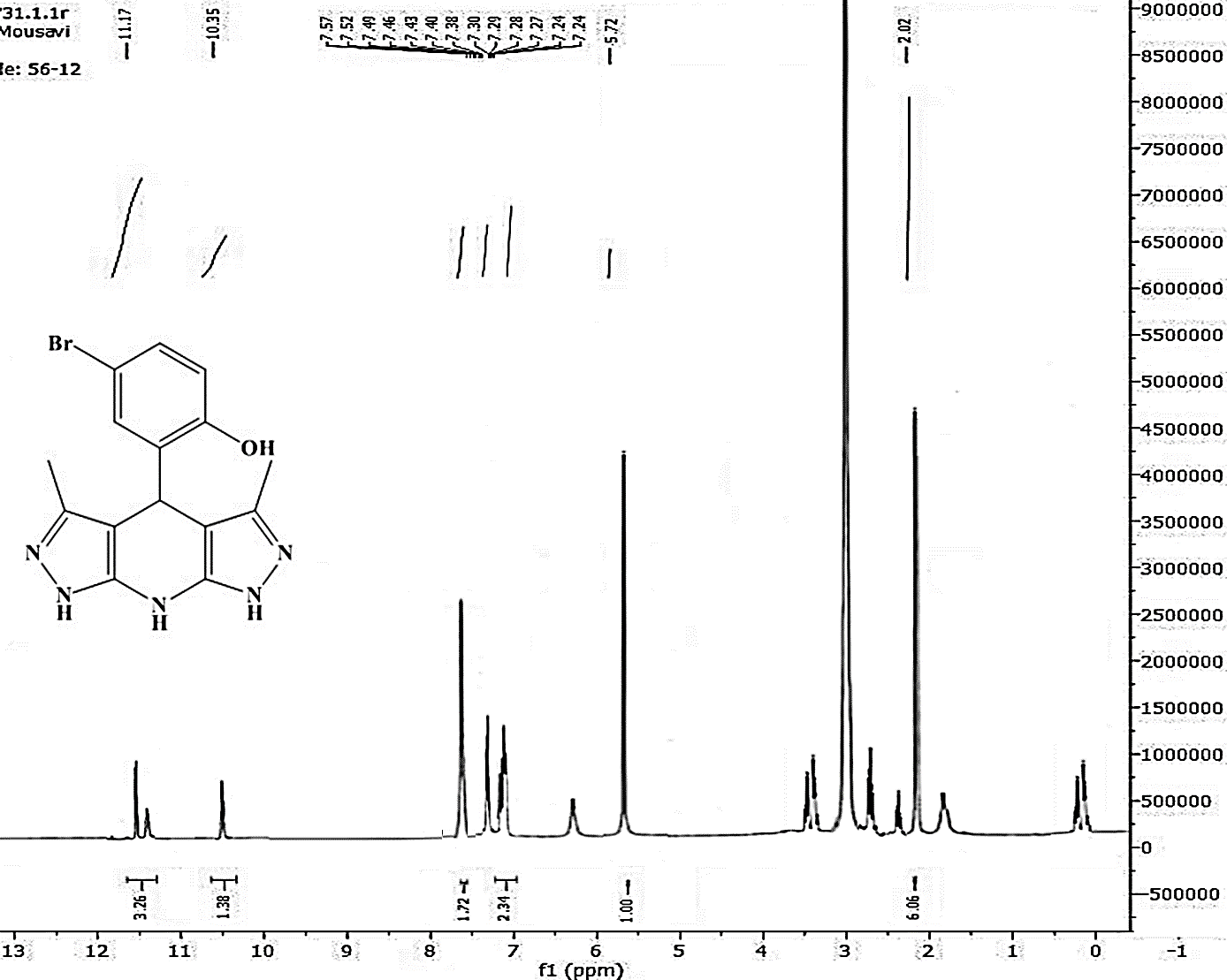
***

**FIGURE S35.** ^1^H NMR spectrum of Compound **5p**


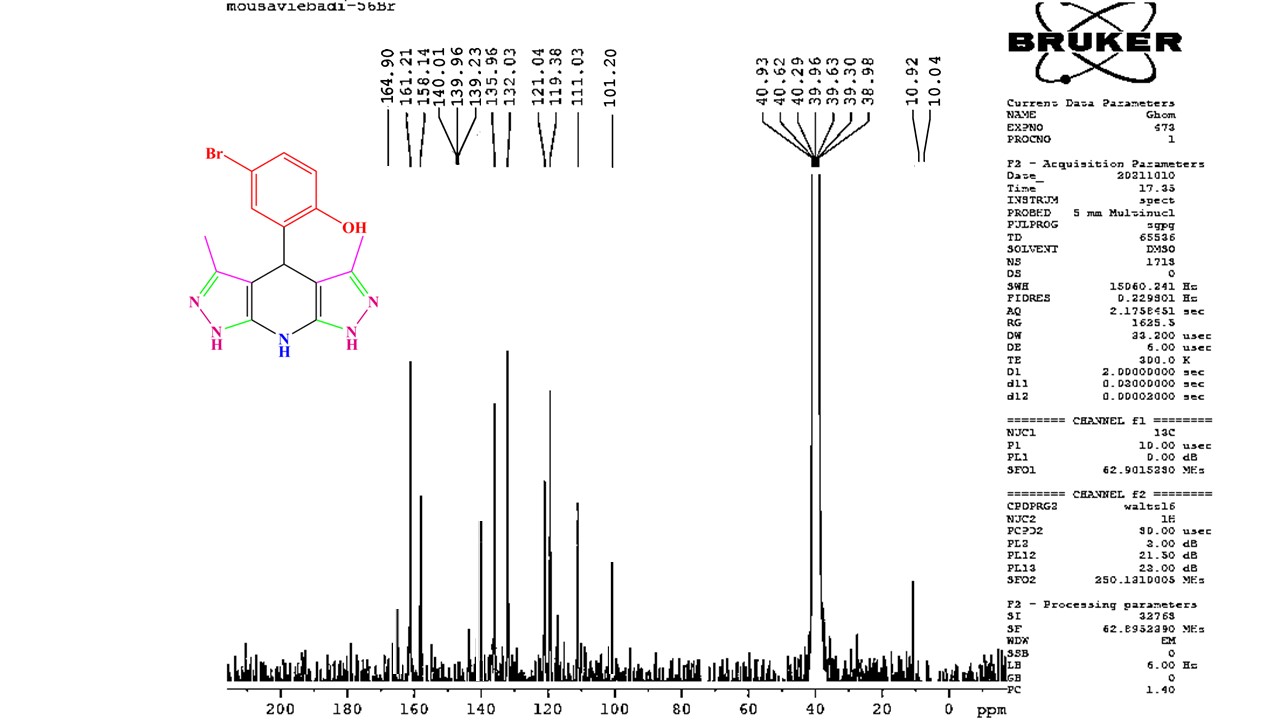


**FIGURE S36.** ^13^C NMR spectrum of Compound **5p**.


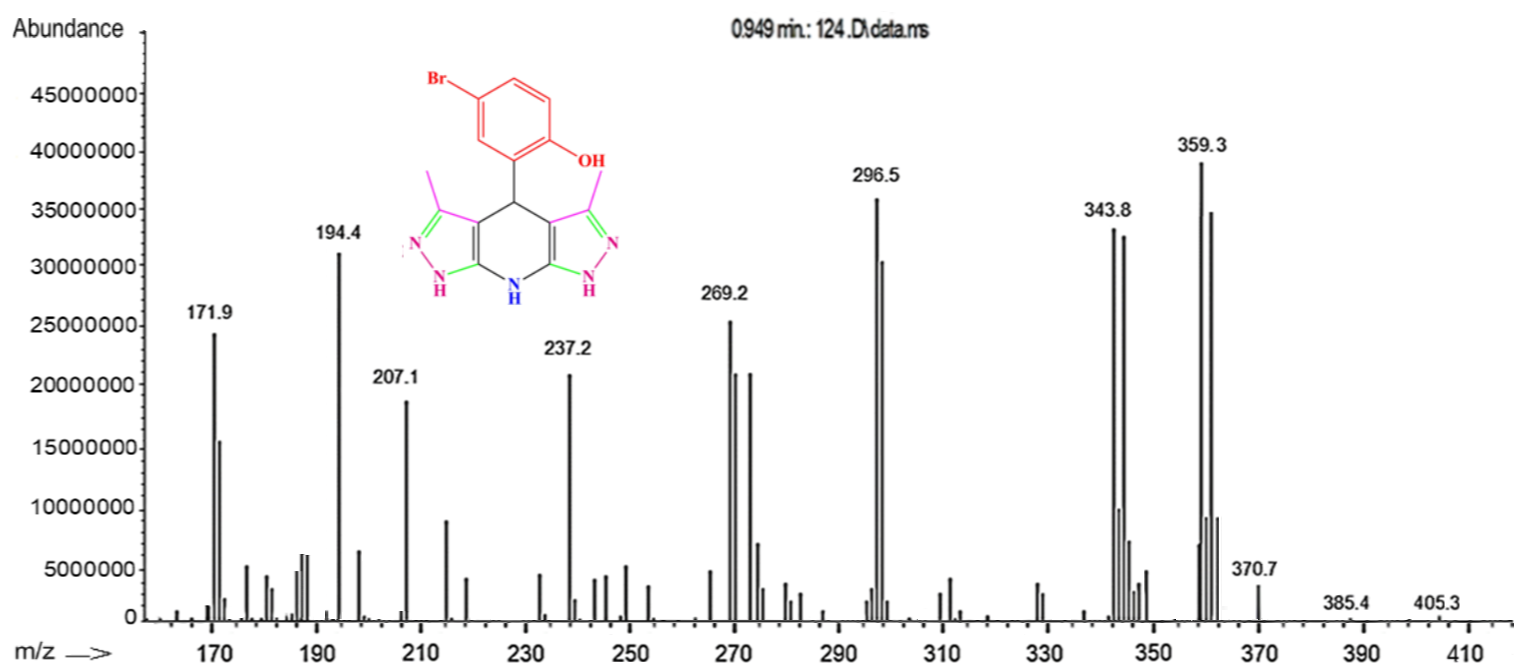


**FIGURE S37.** Mass spectrum of Compound **5p**.


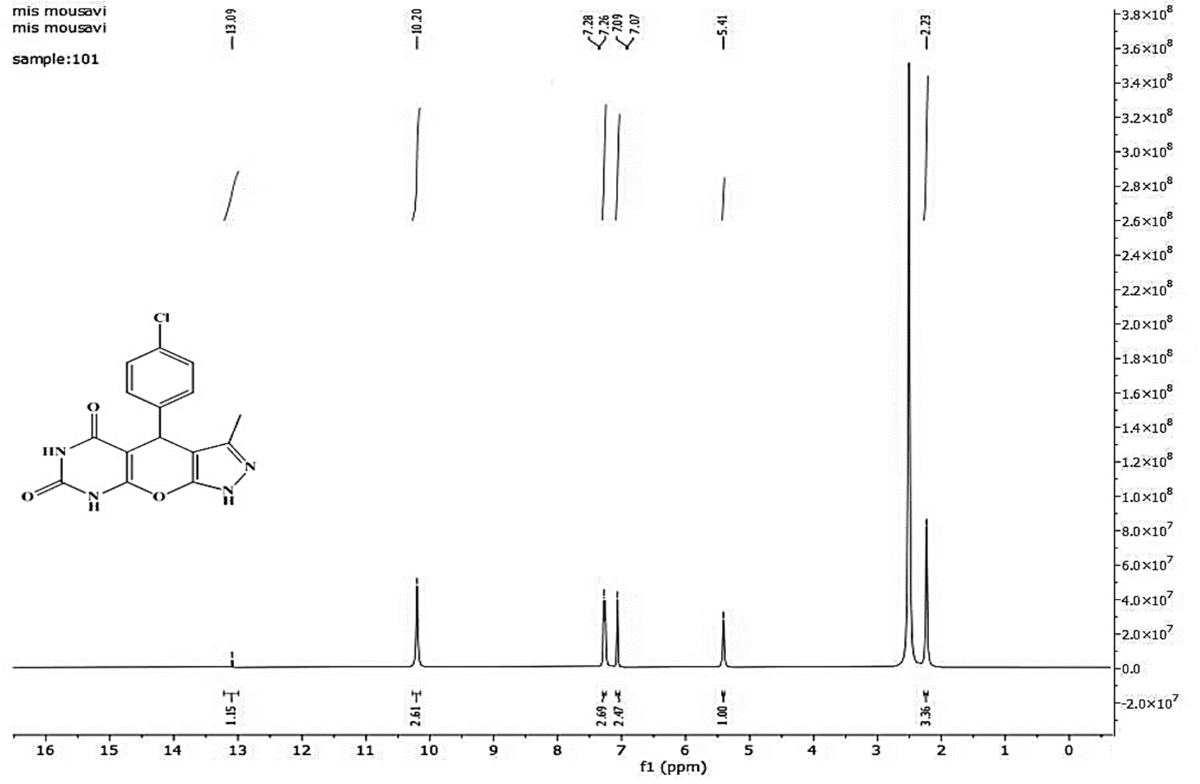


**FIGURE S38.** ^1^H NMR spectrum of Compound **7a**.


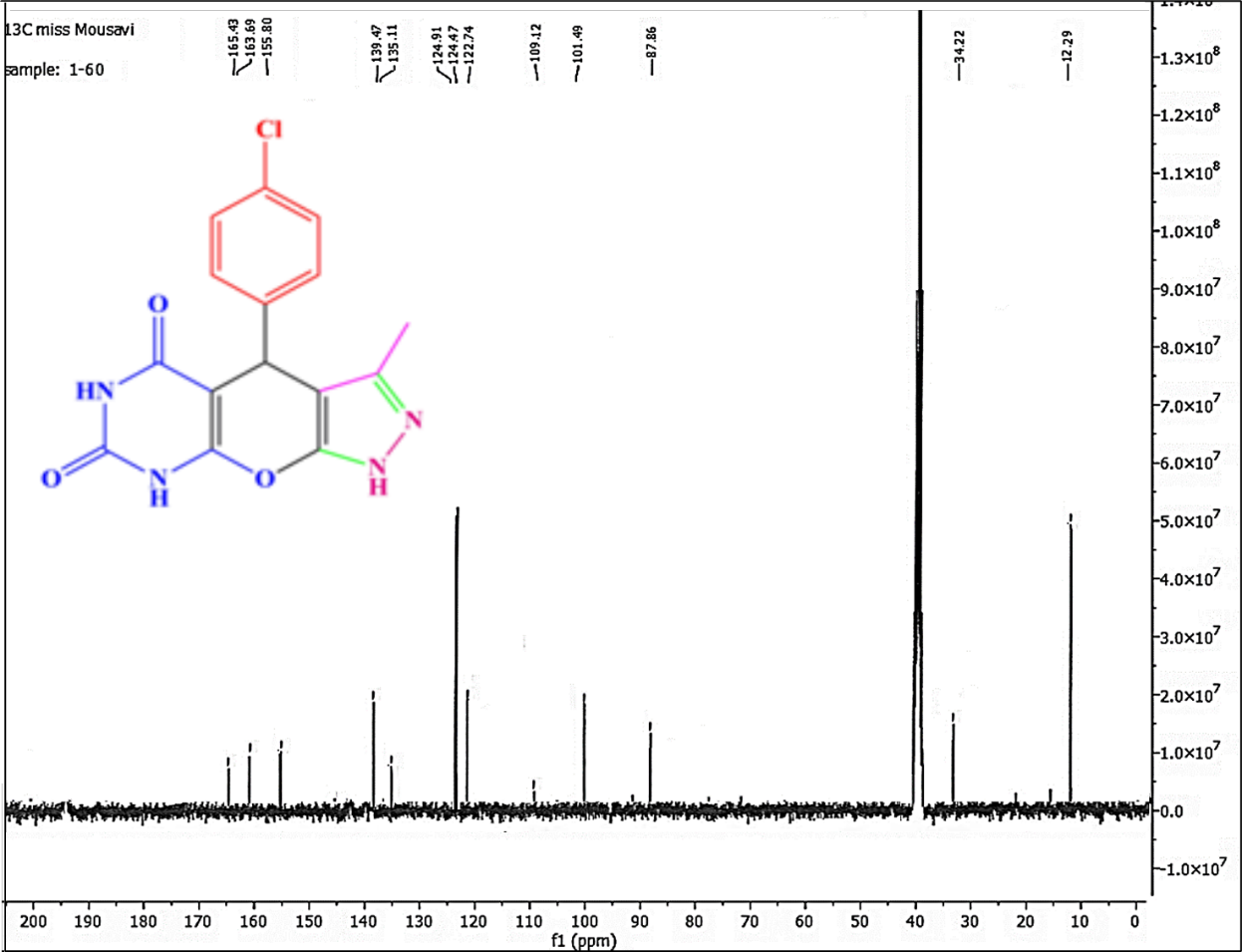


**FIGURE S39.** ^13^C NMR spectrum of Compound **7a**.


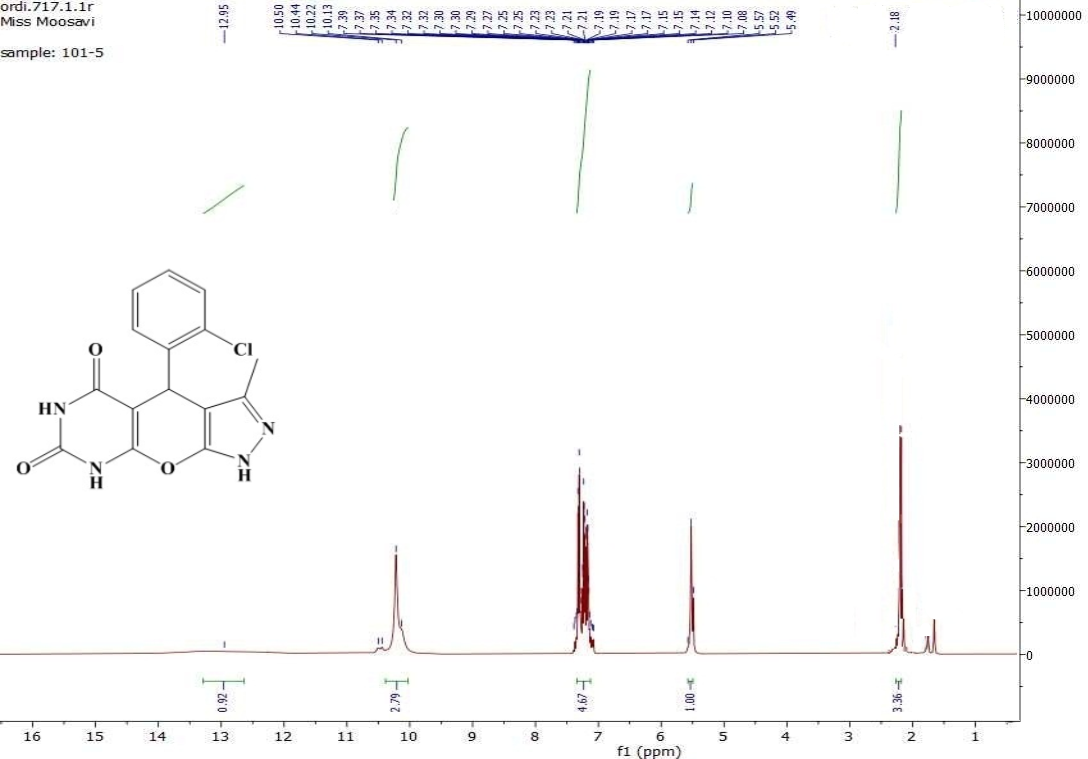


**FIGURE S40.** ^1^H NMR spectrum of Compound **7b**.


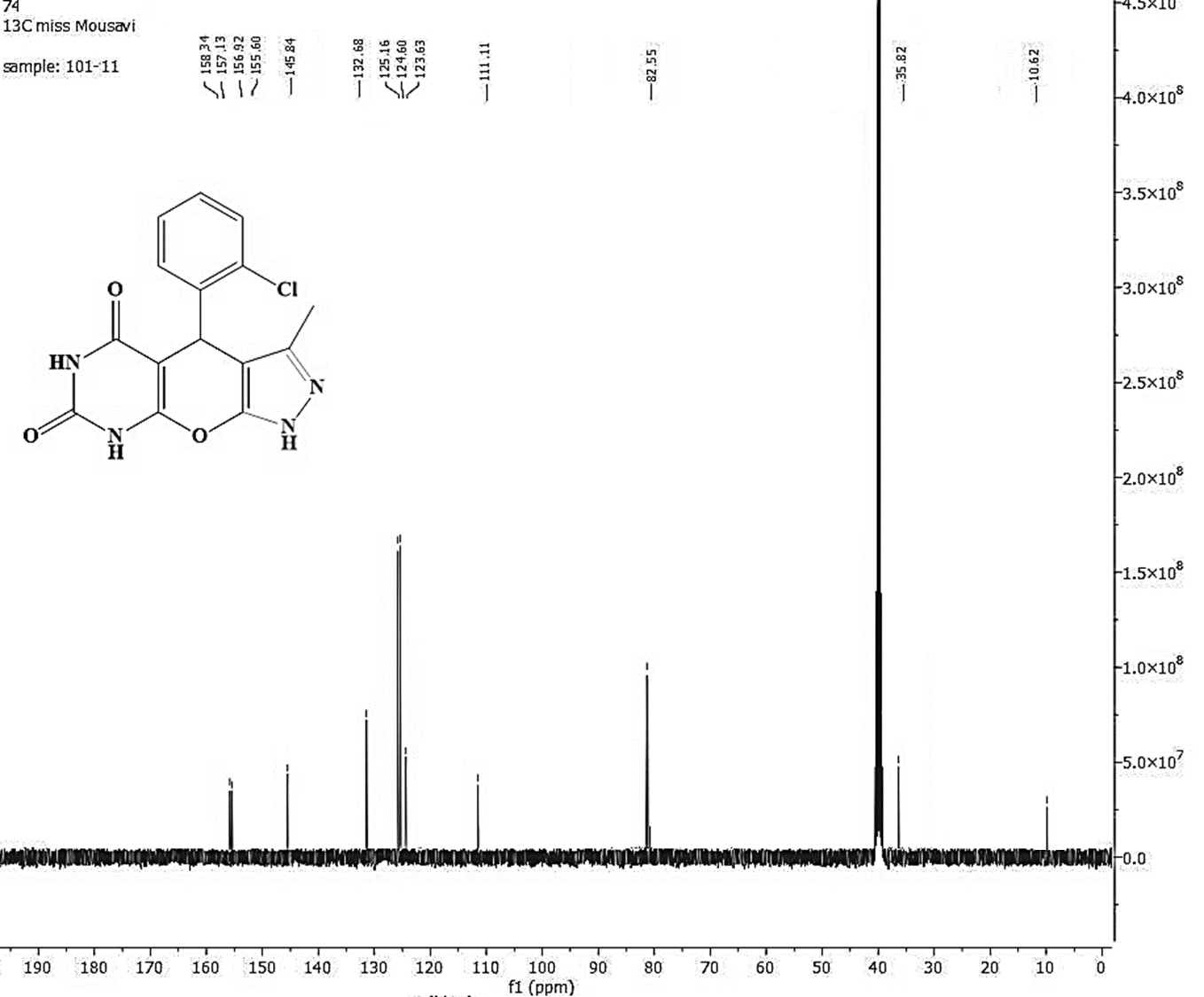


**FIGURE S41.** ^13^C NMR spectrum of Compound **7b**.


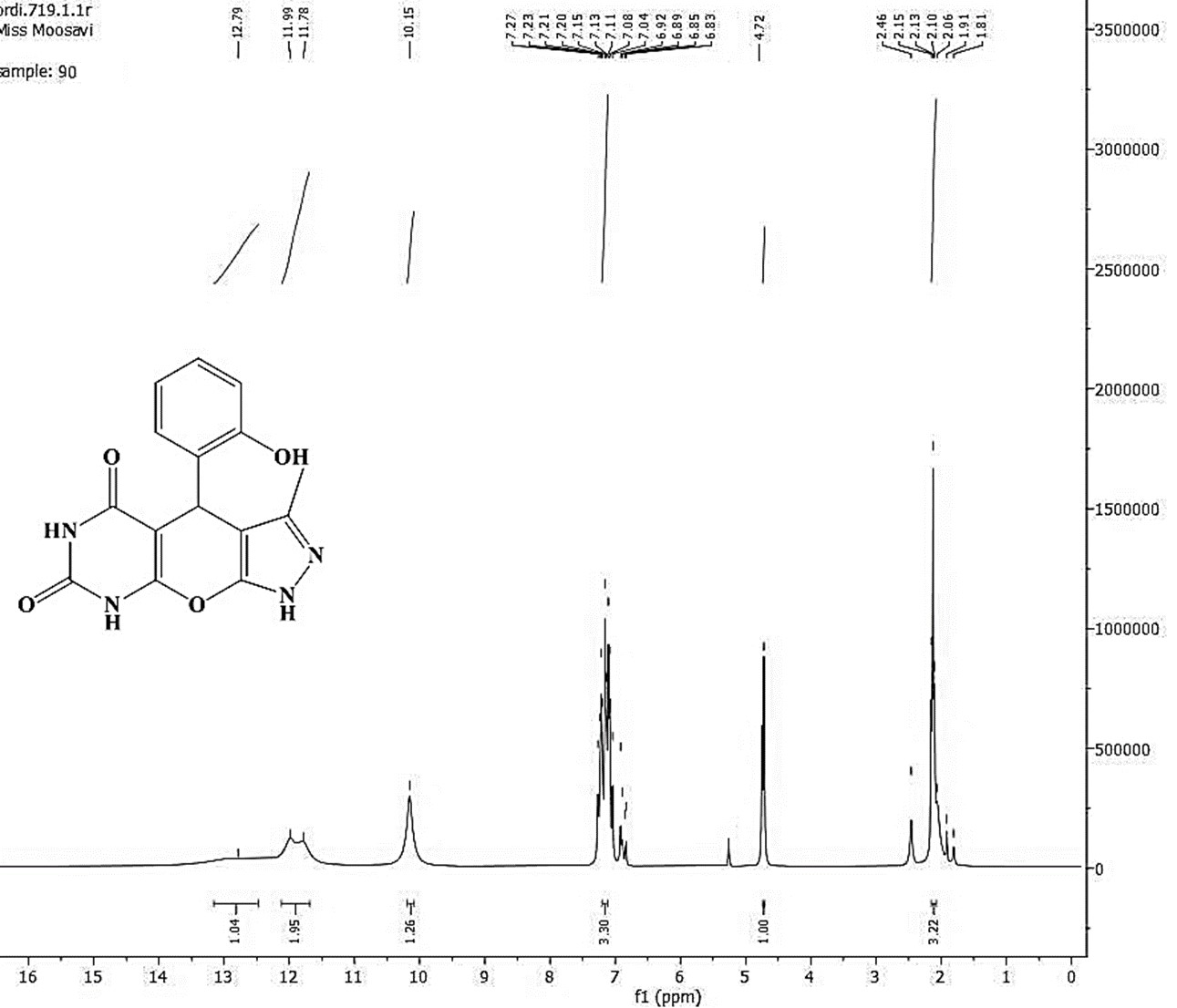


**FIGURE S42.** ^1^H NMR spectrum of Compound **7c**.


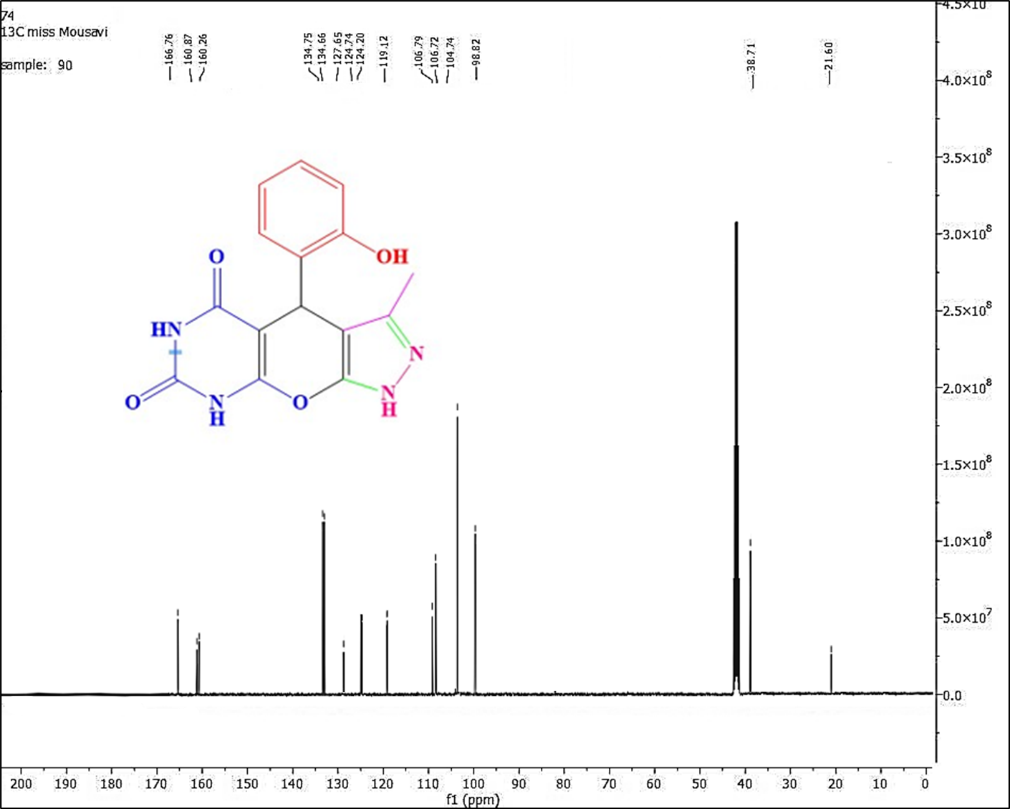


**FIGURE S43.** ^13^C NMR spectrum of Compound **7c**.


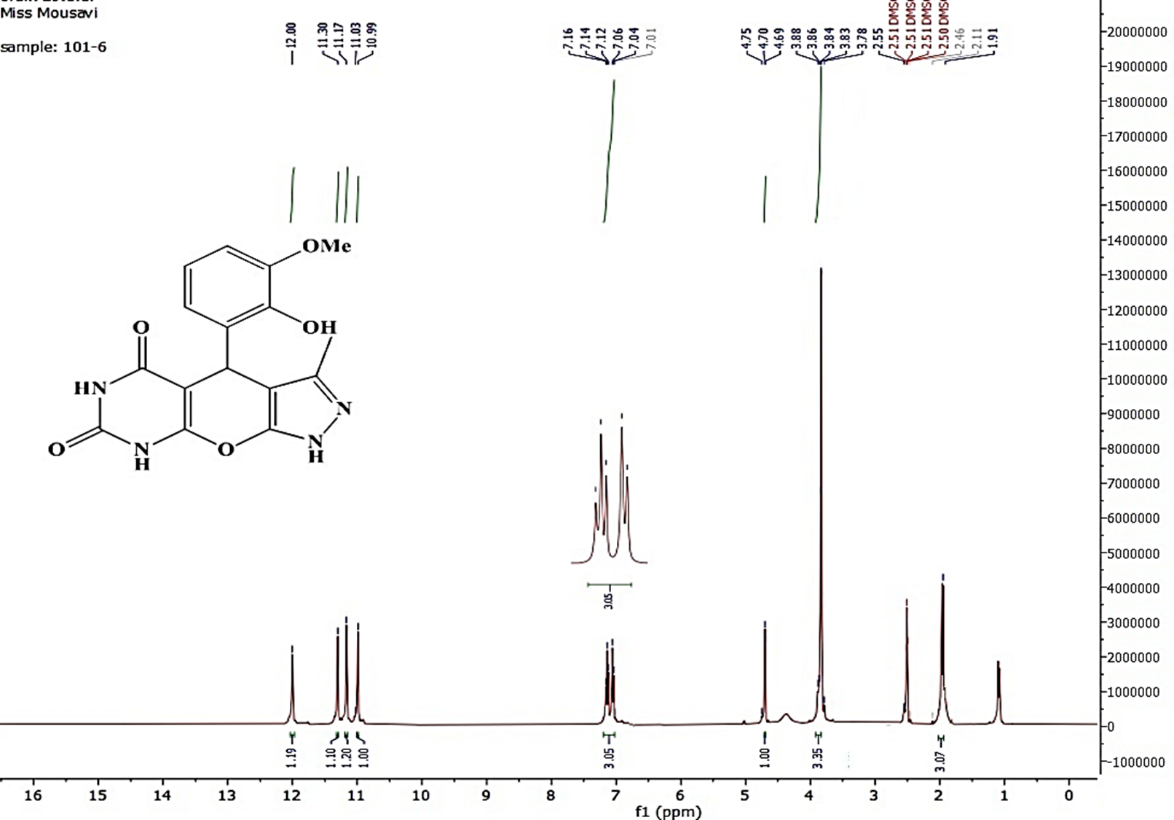


**FIGURE S44.** ^1^H NMR spectrum of Compound **7d.**

***
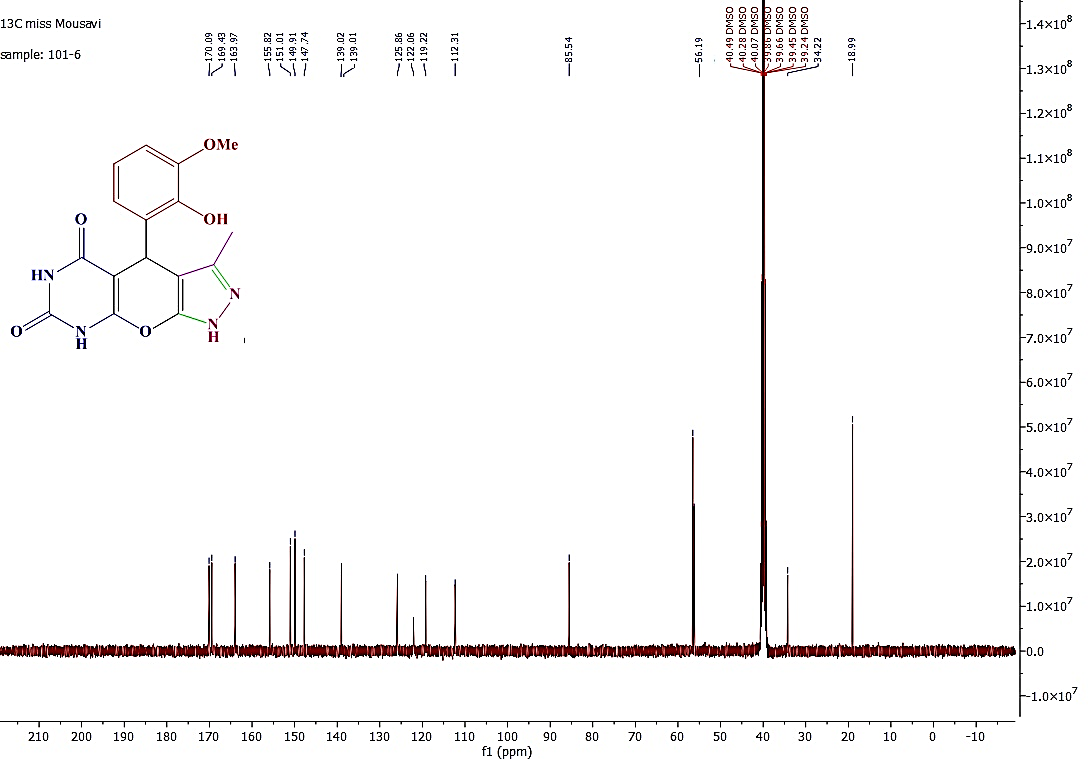
***

**FIGURE S45.** ^13^C NMR spectrum of Compound **7d.**


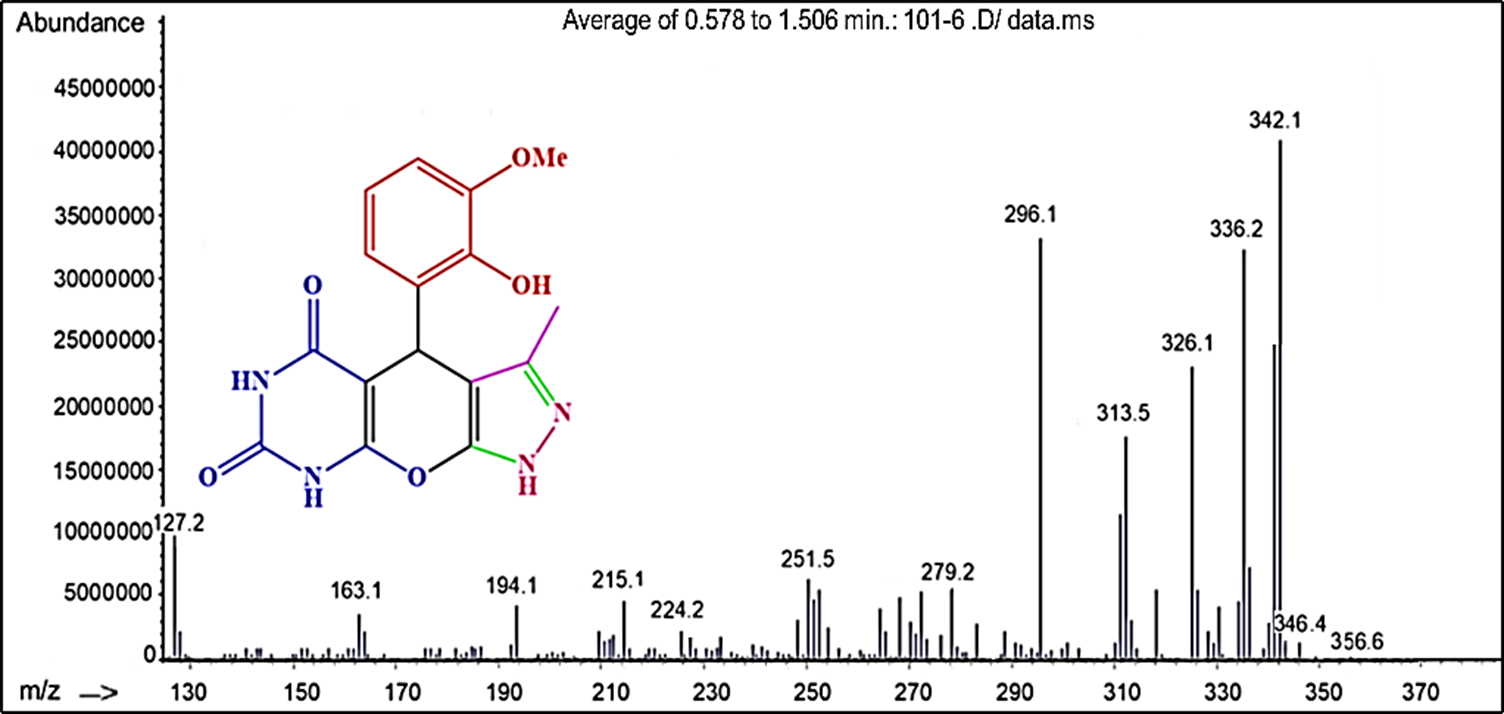


**FIGURE S46.** Mass spectrum of Compound **7d.**


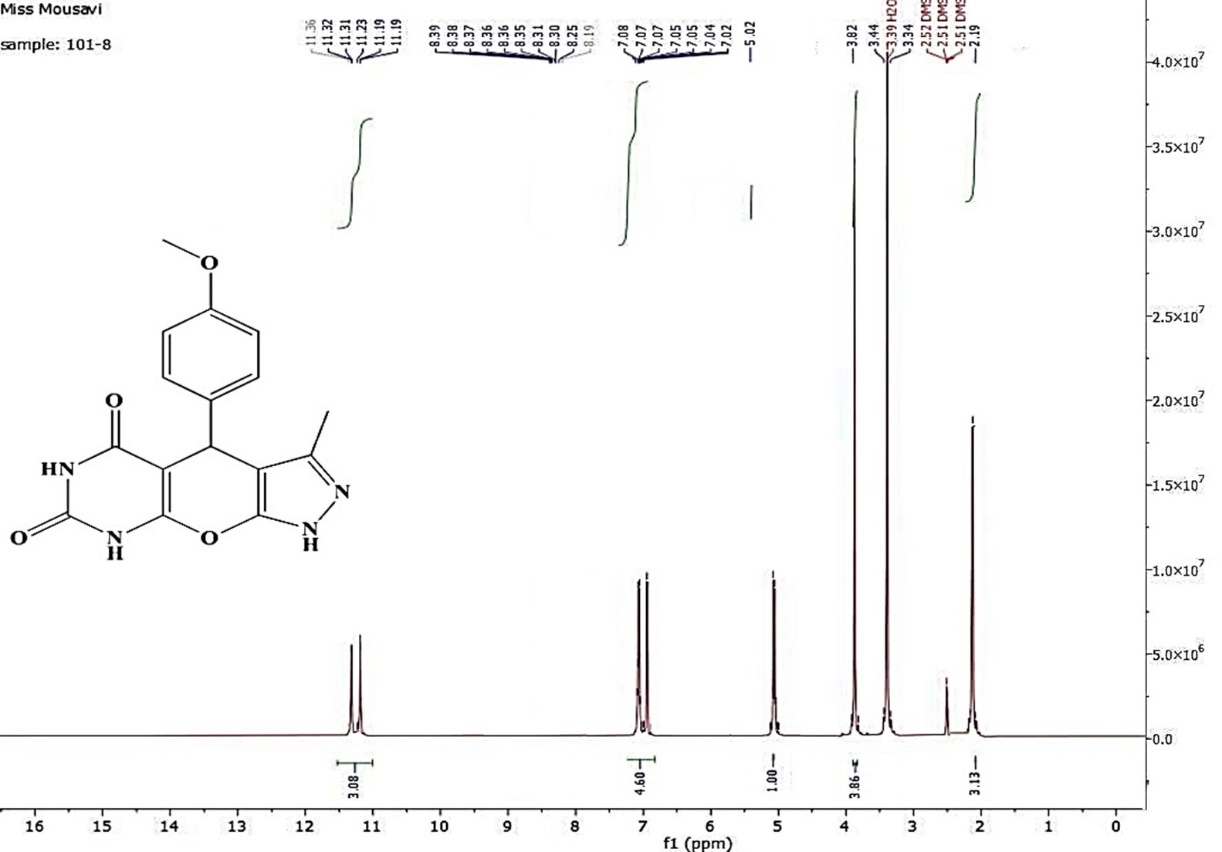


**FIGURE S47.** ^1^H NMR spectrum of Compound **7e.**


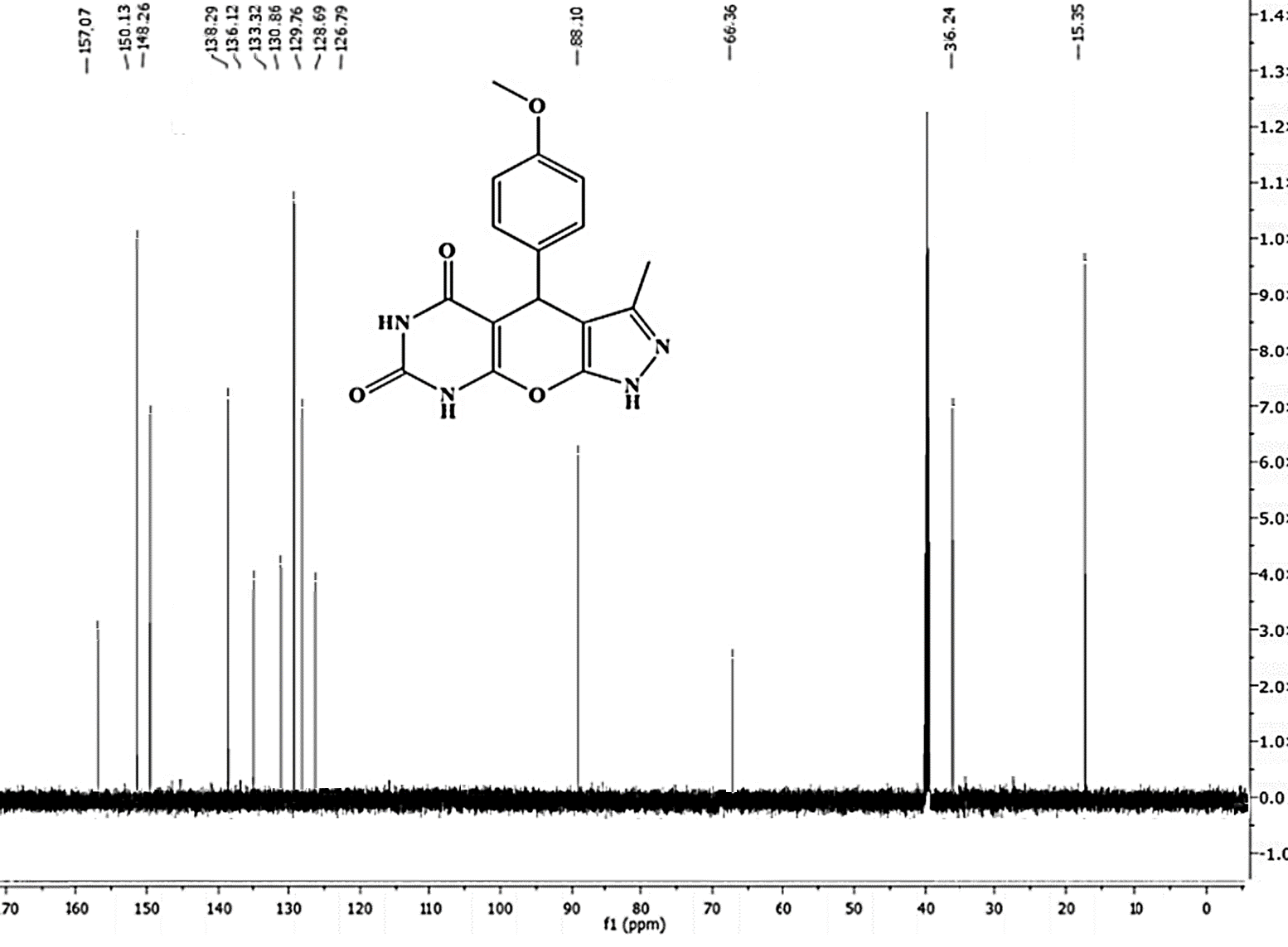


**FIGURE S48.** ^13^C NMR spectrum of Compound **7e.**


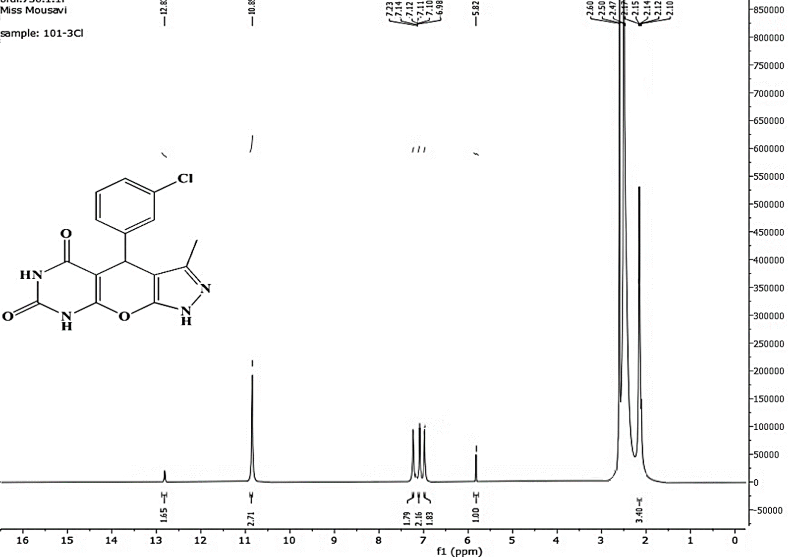


**FIGURE S49.** ^1^H NMR spectrum of Compound **7f.**


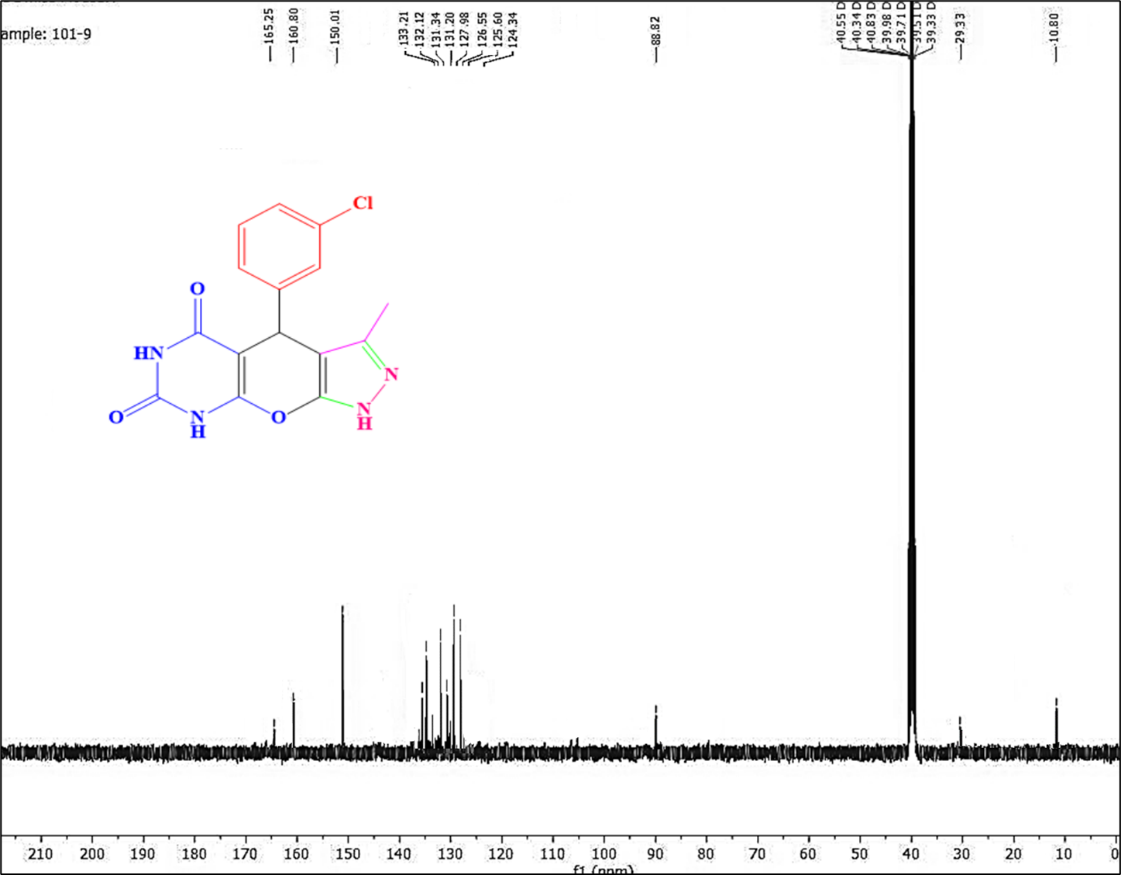


**FIGURE S50.** ^13^C NMR spectrum of Compound **7f.**


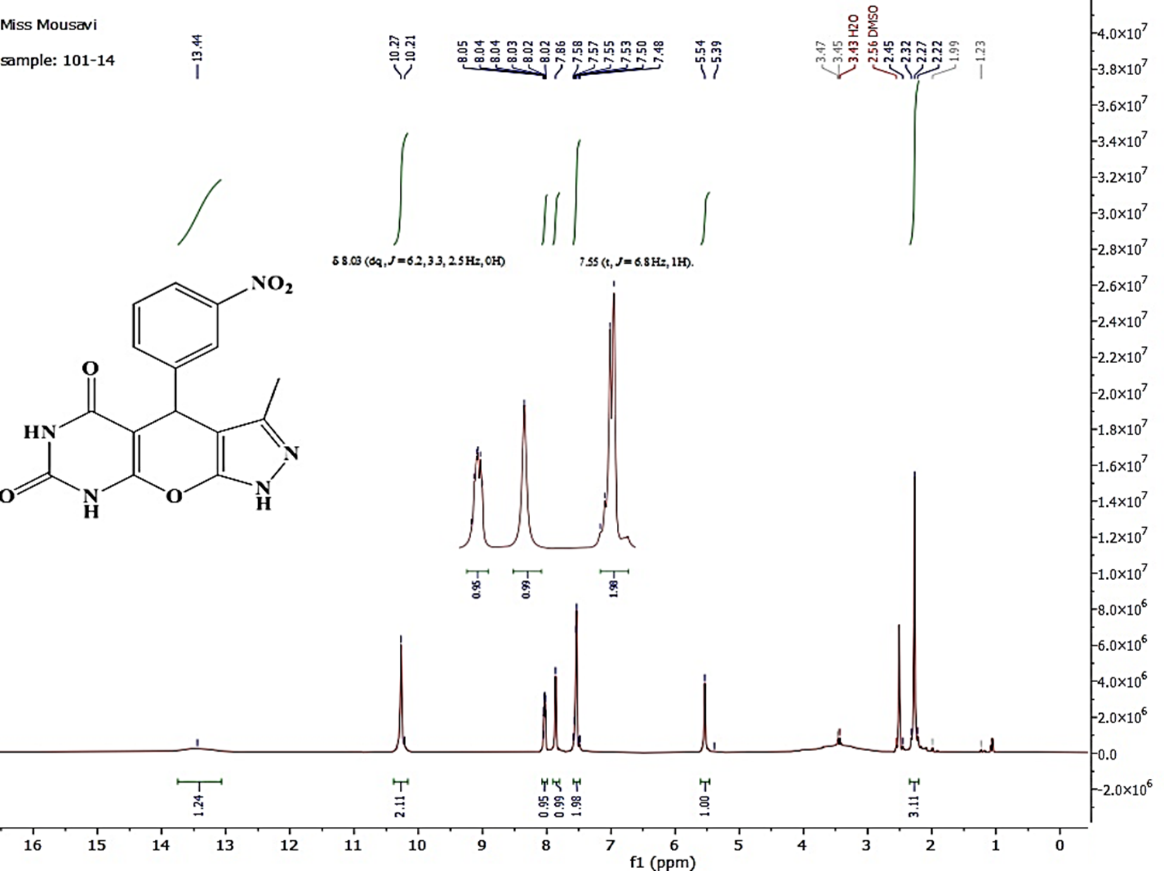


**FIGURE S51.** ^1^H NMR spectrum of Compound **7g.**


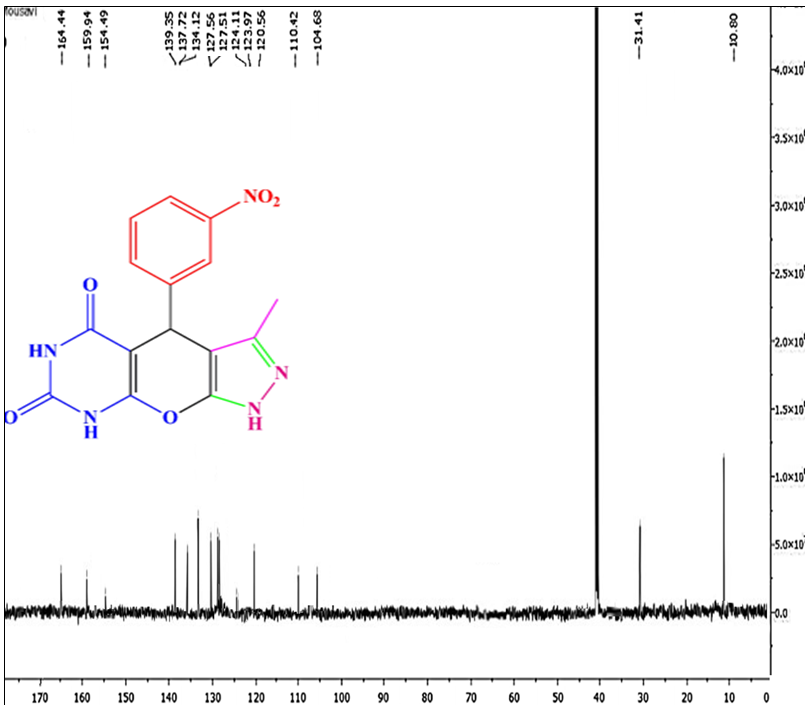


**FIGURE S52.** ^13^C NMR spectrum of Compound **7g.**


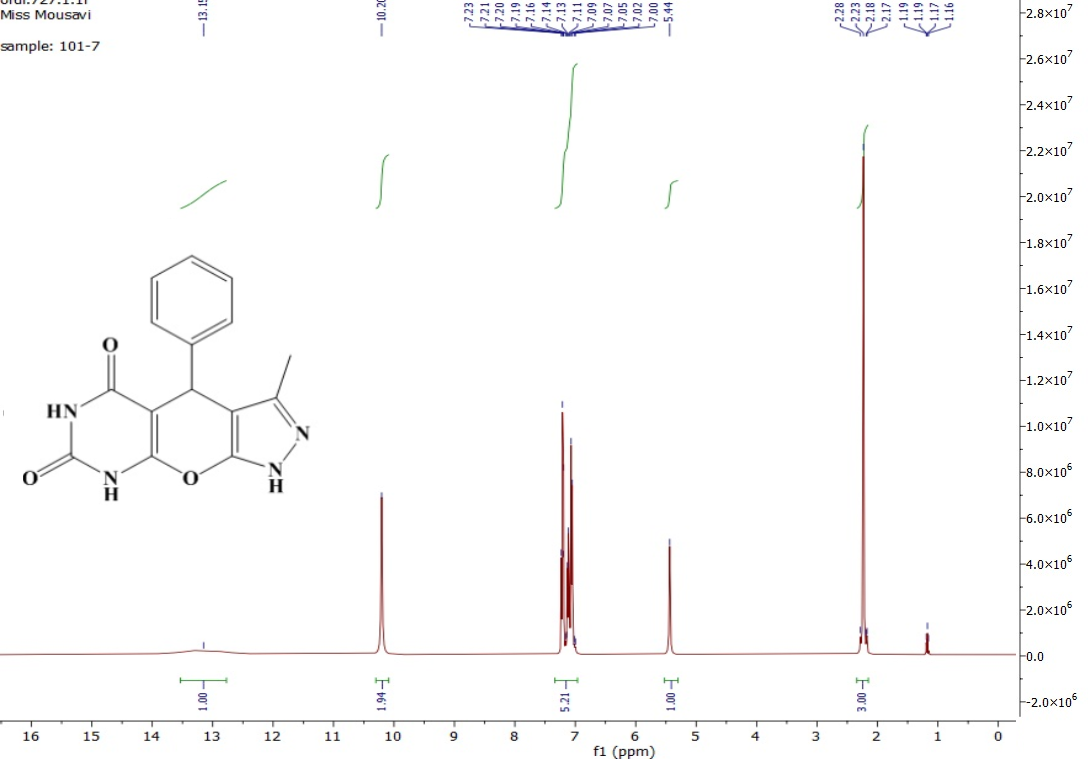


**FIGURE S53.** ^1^H NMR spectrum of Compound **7h.**


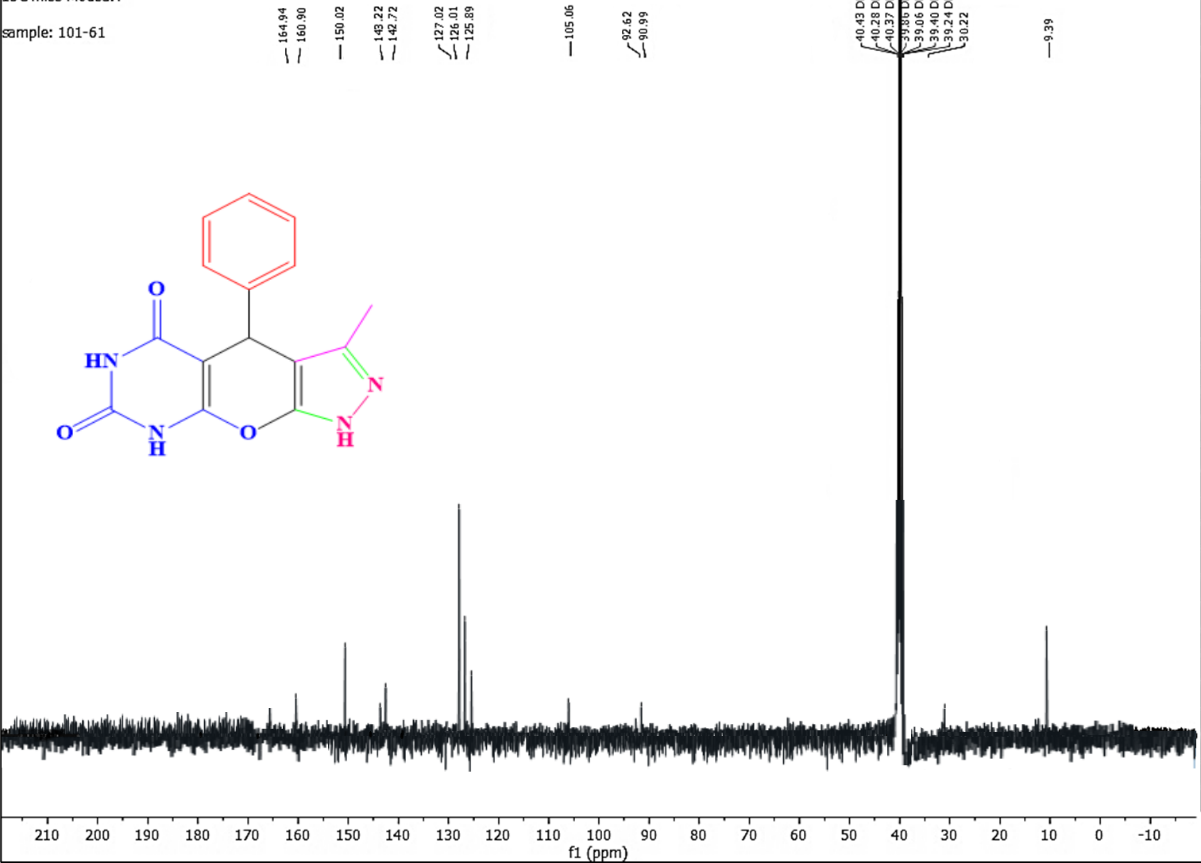


**FIGURE S54.** ^13^C NMR spectrum of Compound **7h.**


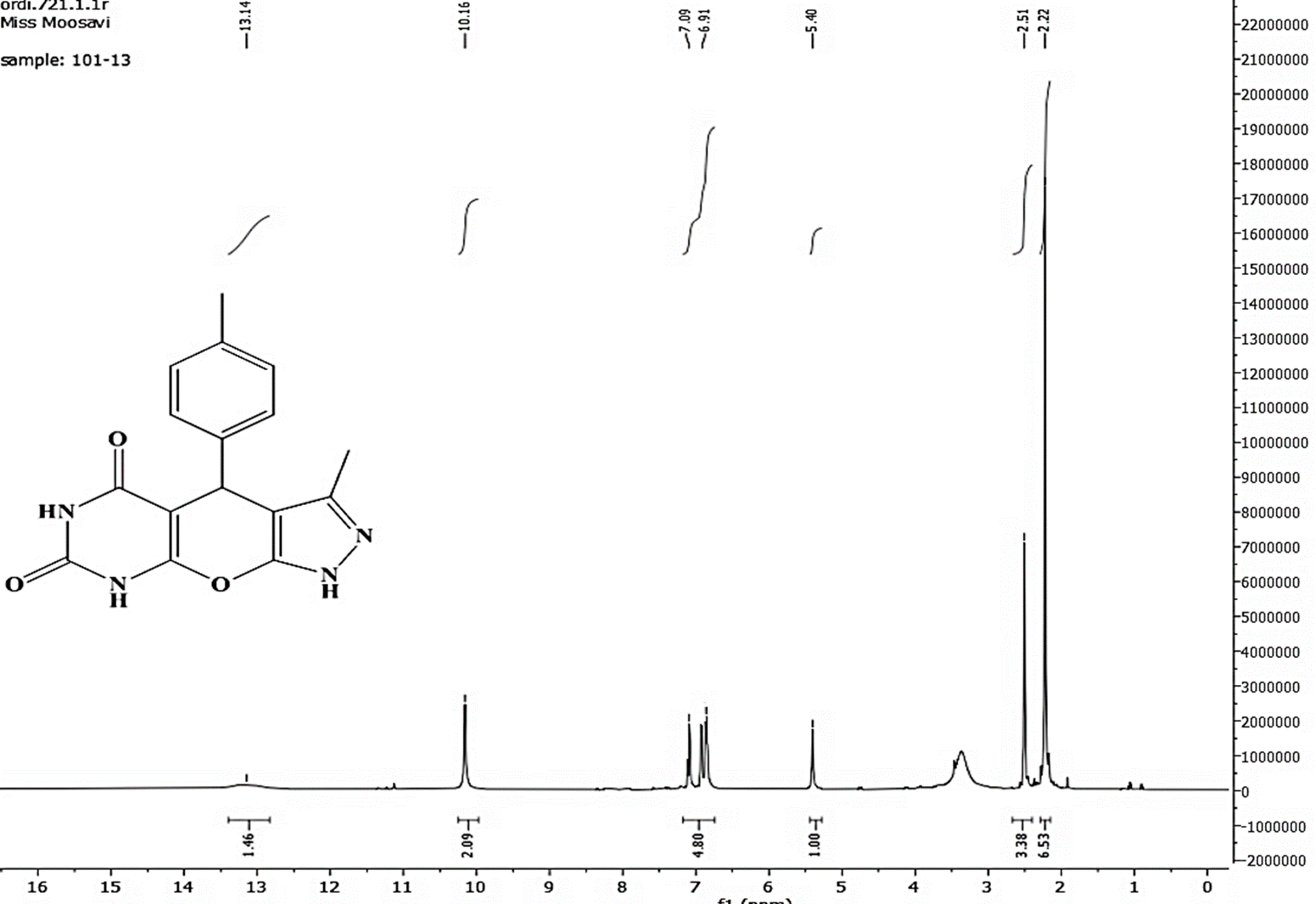


**FIGURE S55.** ^1^H NMR spectrum of Compound **7i.**


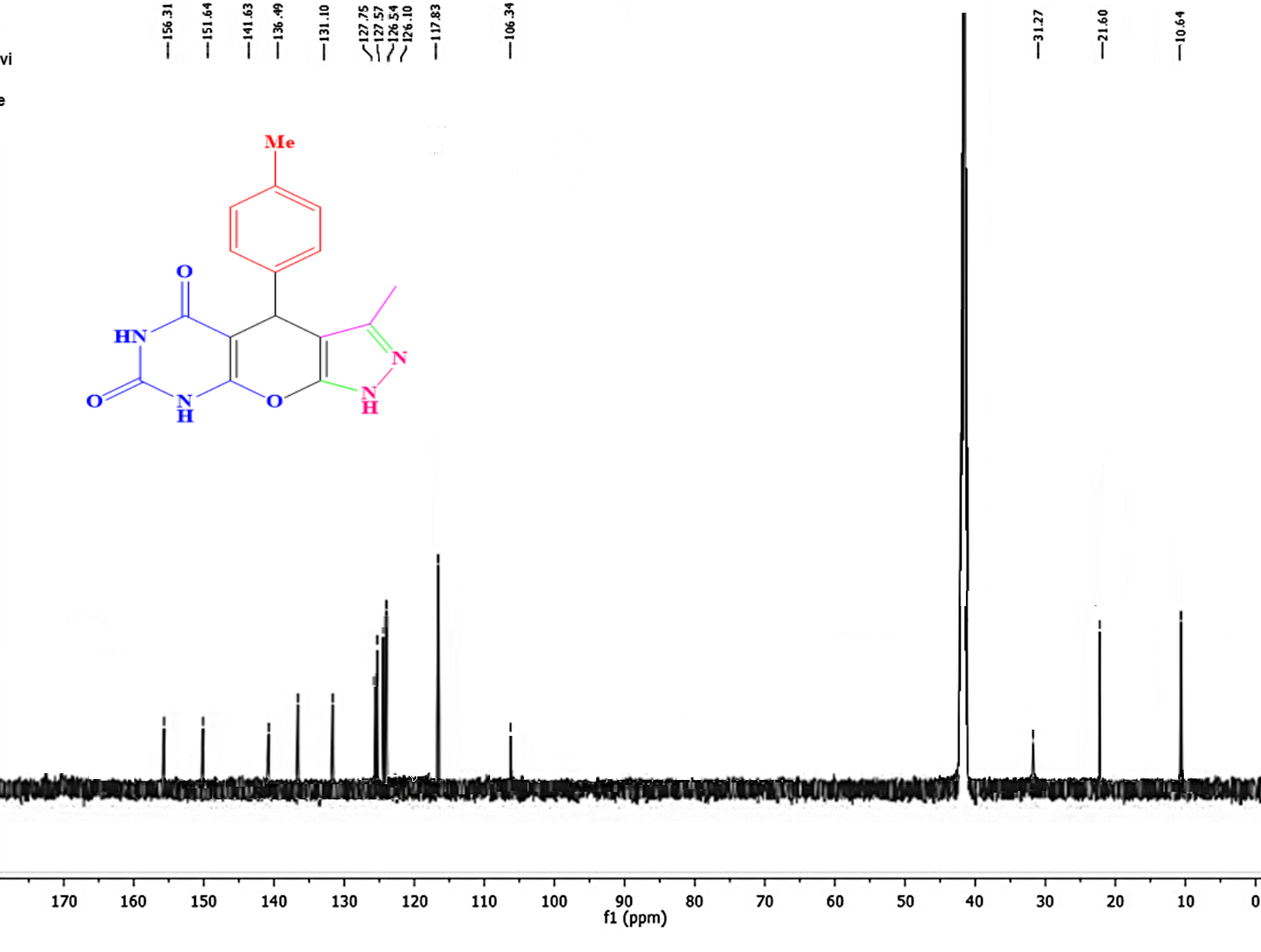


**FIGURE S56.** ^13^C NMR spectrum of Compound **7i.**

***
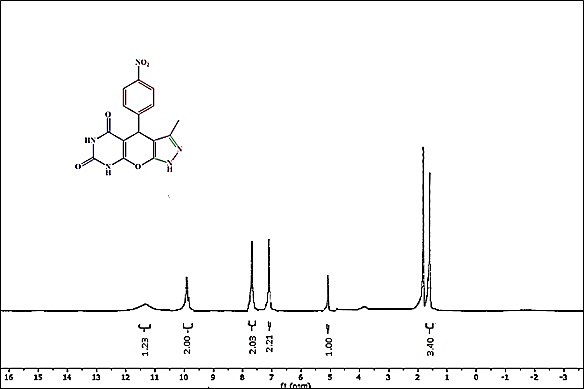
***

**FIGURE S57.** ^1^H NMR spectrum of Compound **7j.**


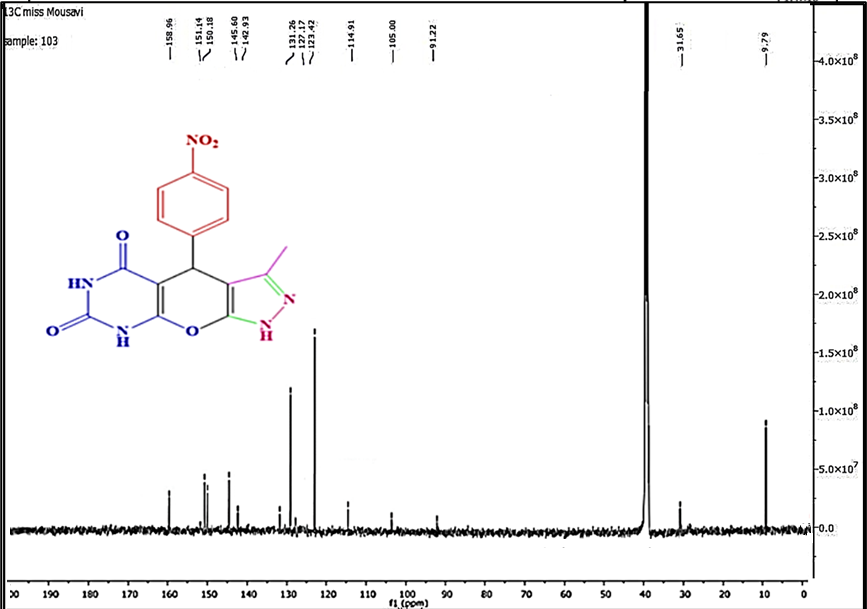


**FIGURE S58.** ^13^C NMR spectrum of Compound **7j.**

***
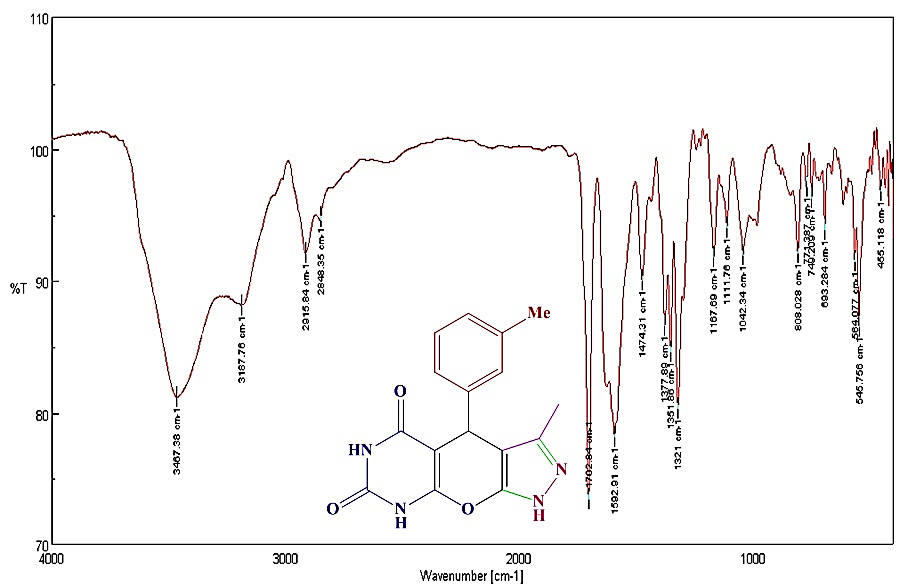
***

**FIGURE S59.** FT-IR spectrum of Compound **7k.**


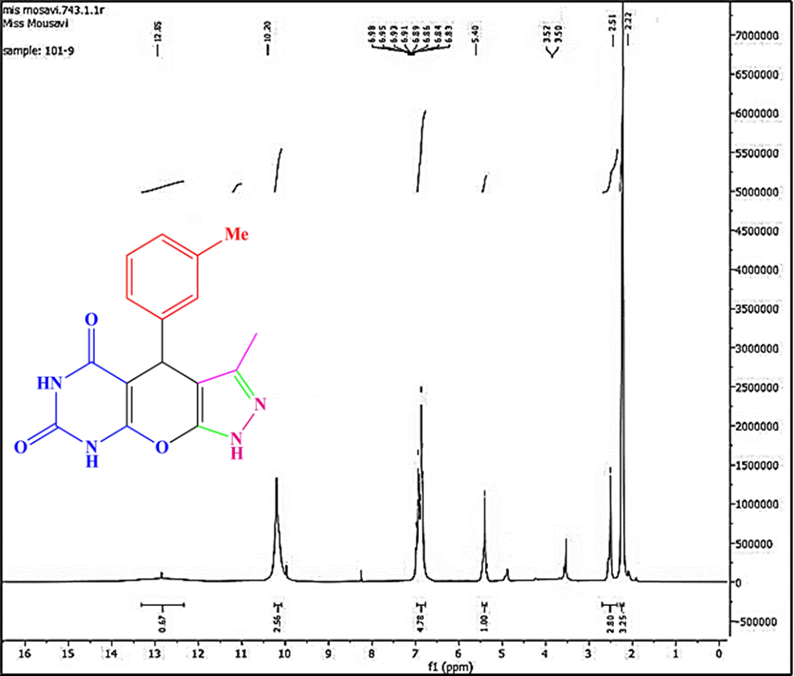


**FIGURE S60.** ^1^H NMR spectrum of Compound **7k.**

***
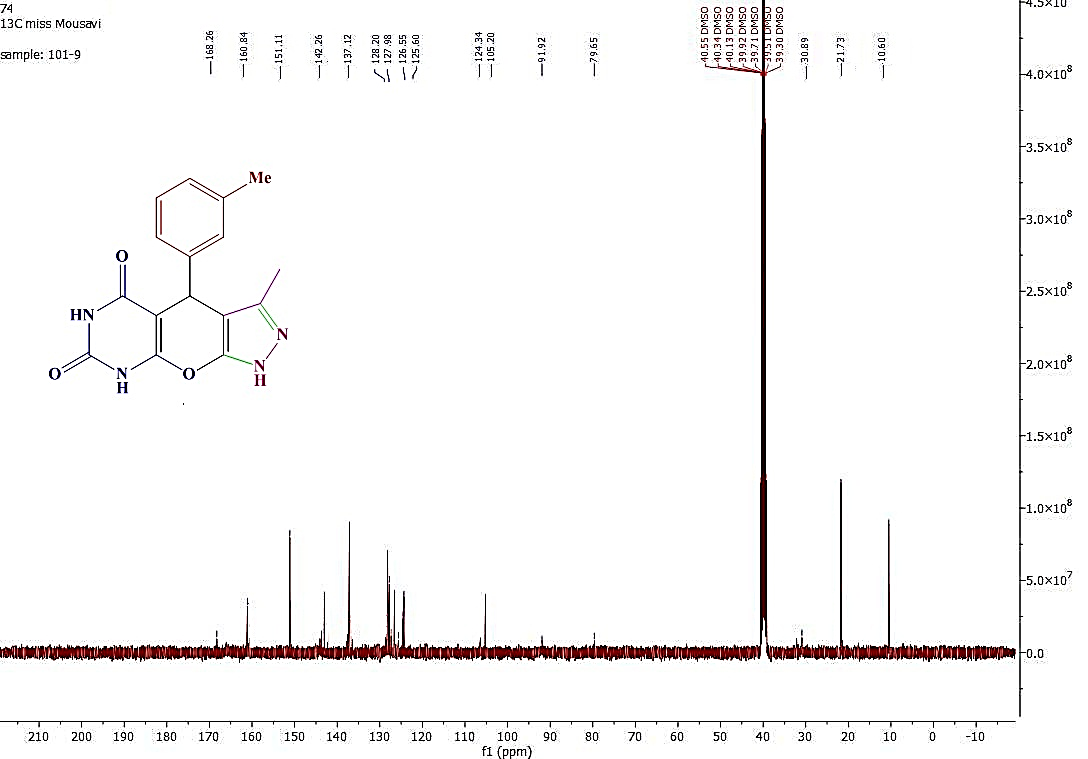
***

**FIGURE S61.** ^13^C NMR spectrum of Compound **7k.**


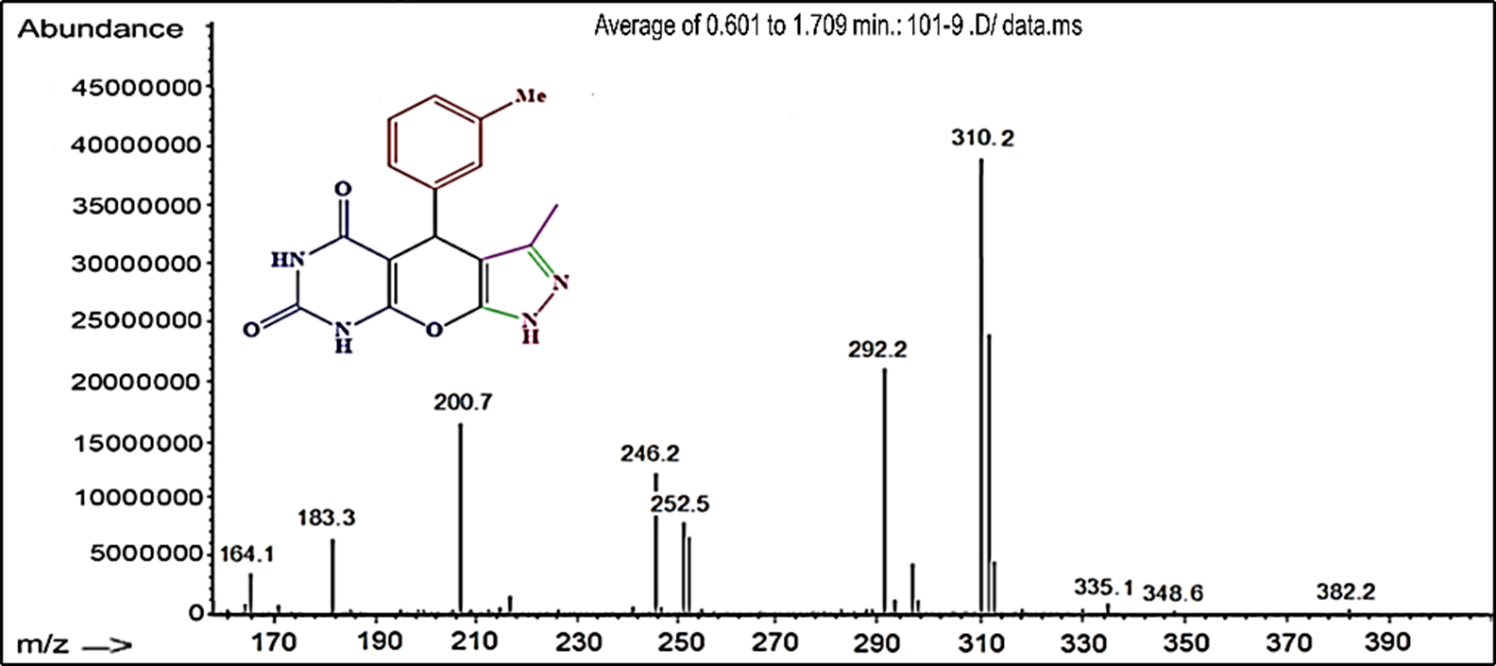


**FIGURE S62.** Mass spectrum of Compound **7k.**


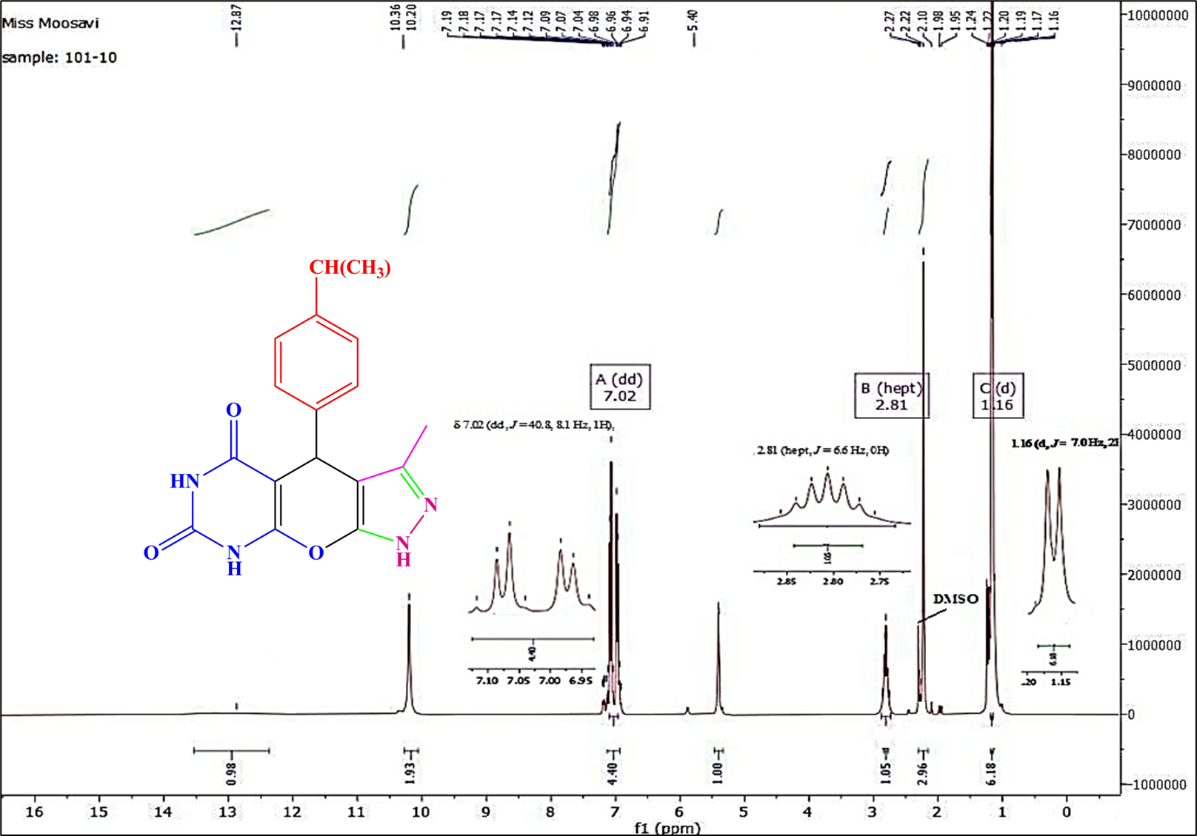


**FIGURE S63.** ^1^H NMR spectrum of Compound **7l.**


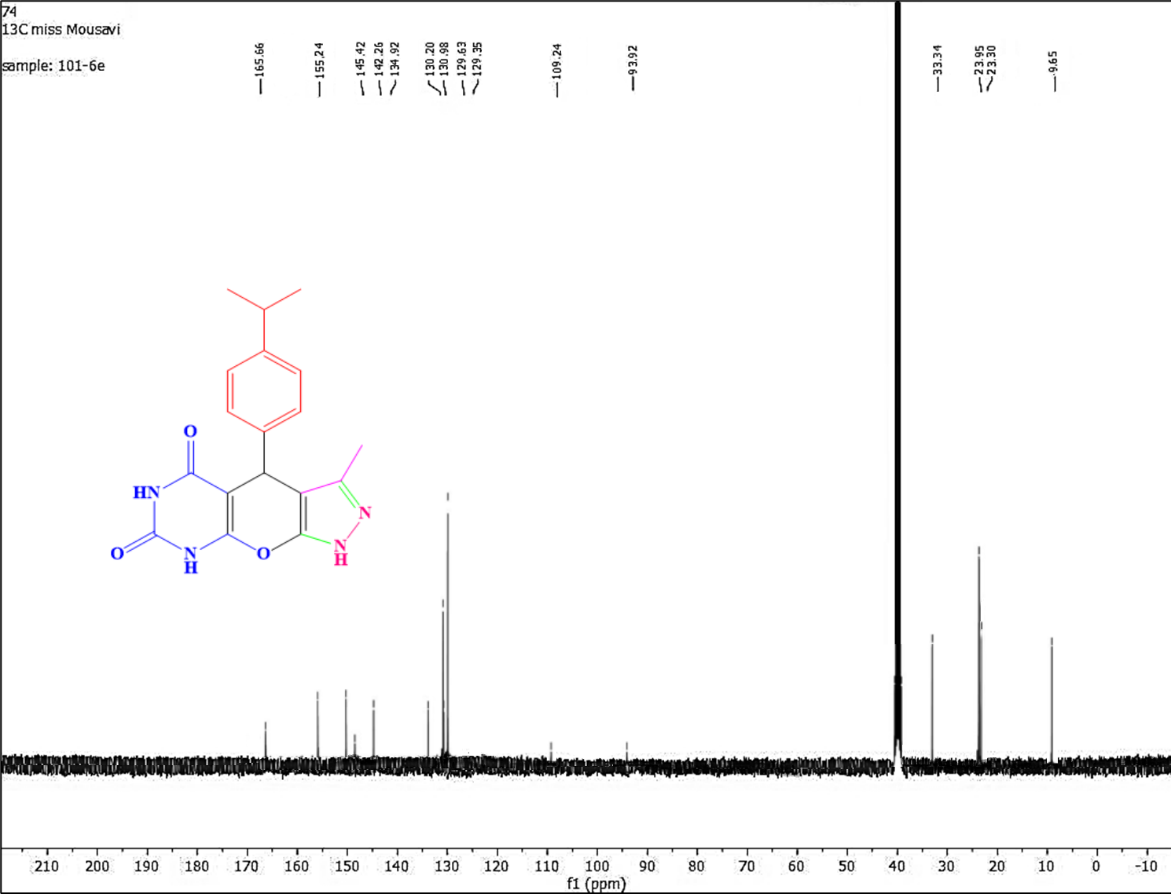


**FIGURE S64.** ^13^C NMR spectrum of Compound **7l.**


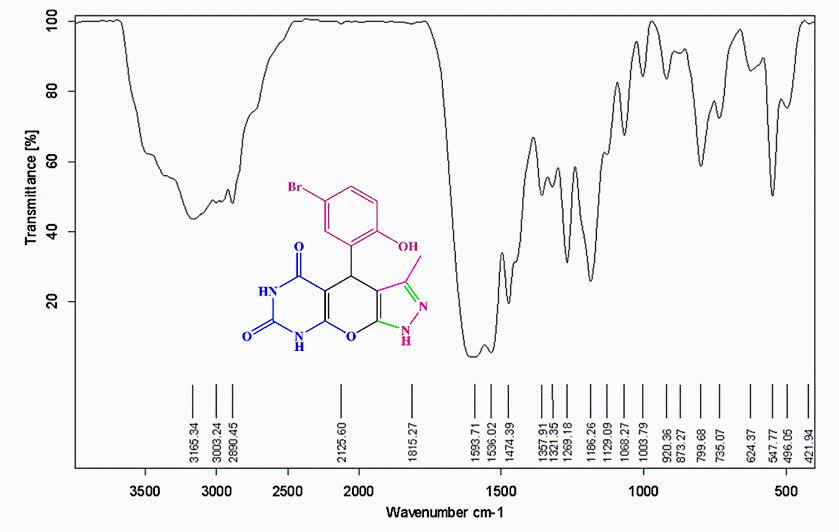


**FIGURE S65.** FT-IR spectrum of Compound **7m.**


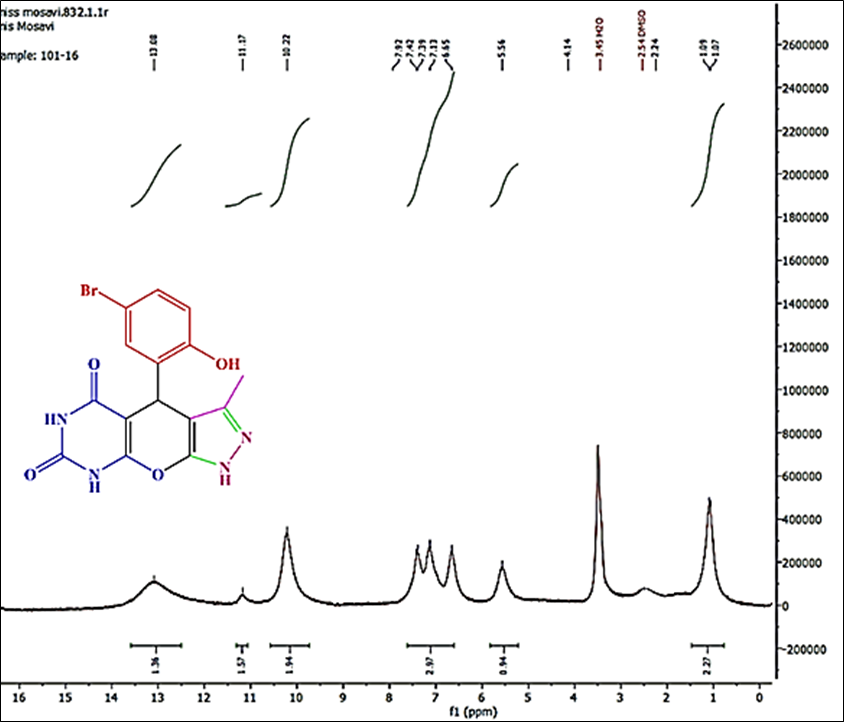


**FIGURE S66.** ^1^H NMR spectrum of Compound **7m.**


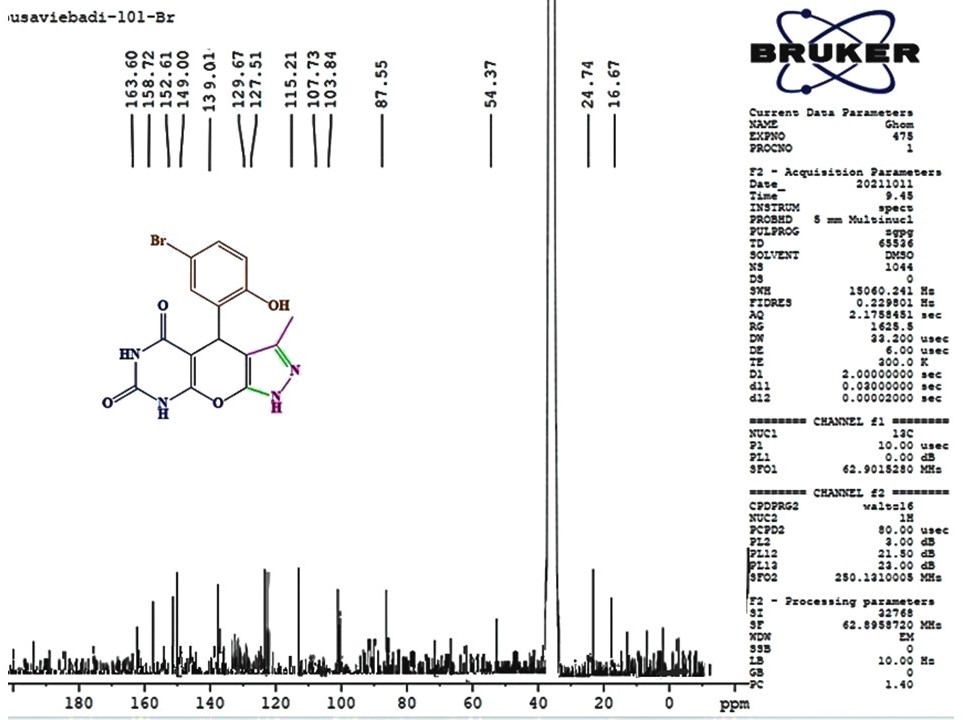


**FIGURE S67.** ^13^C NMR spectrum of Compound **7m.**


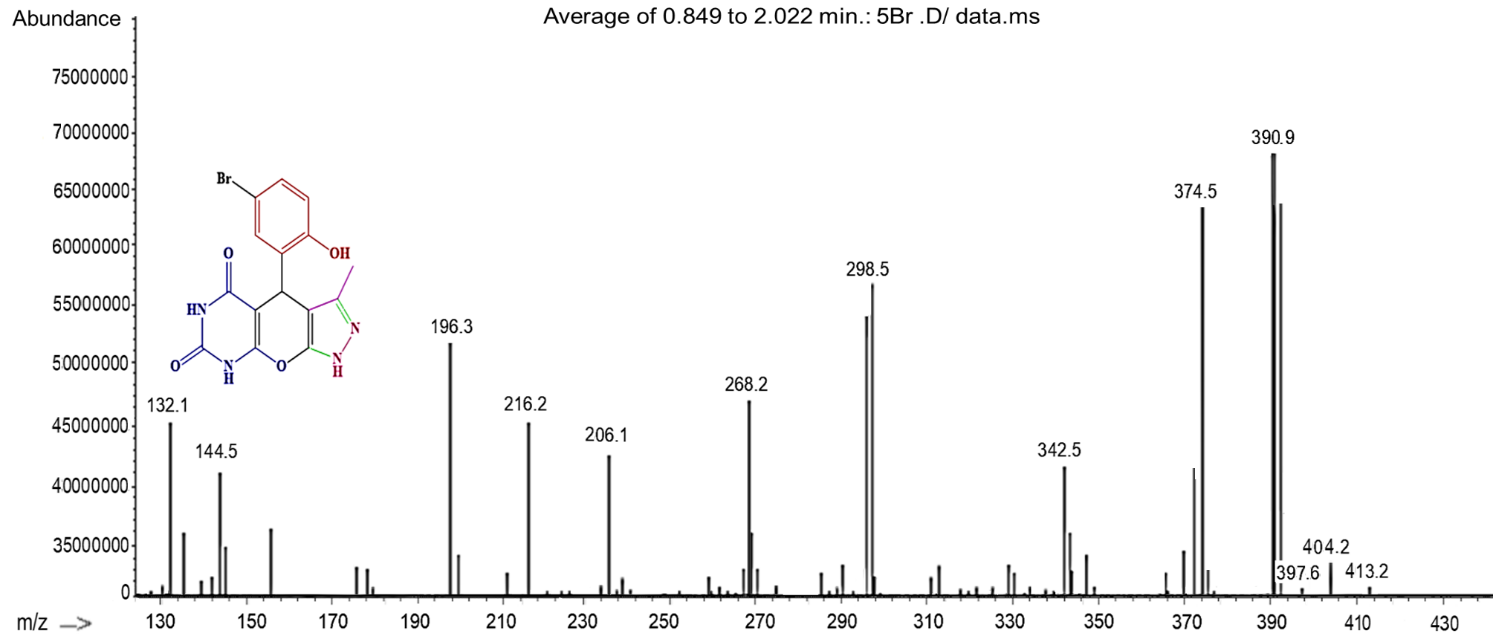


**FIGURE S68.** Mass spectrum of Compound **7m**


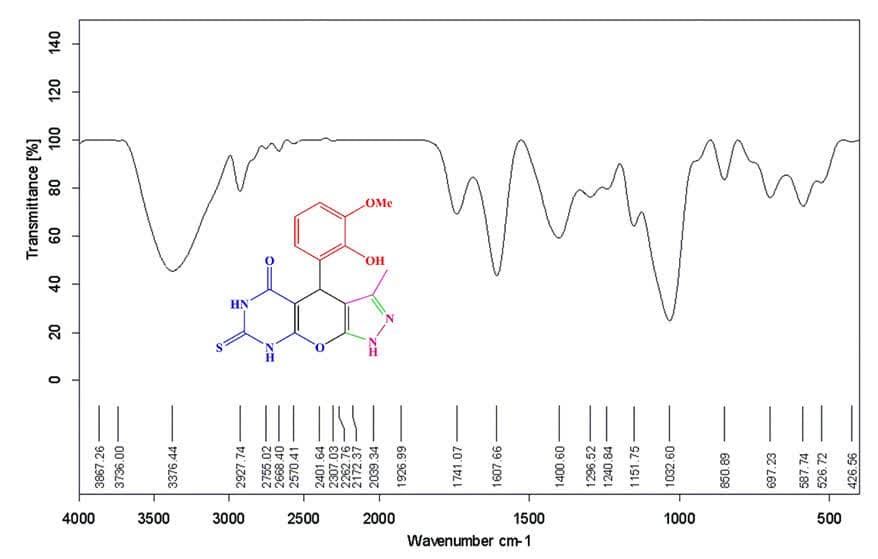


**FIGURE S69.** FT-IR spectrum of Compound **7n.**


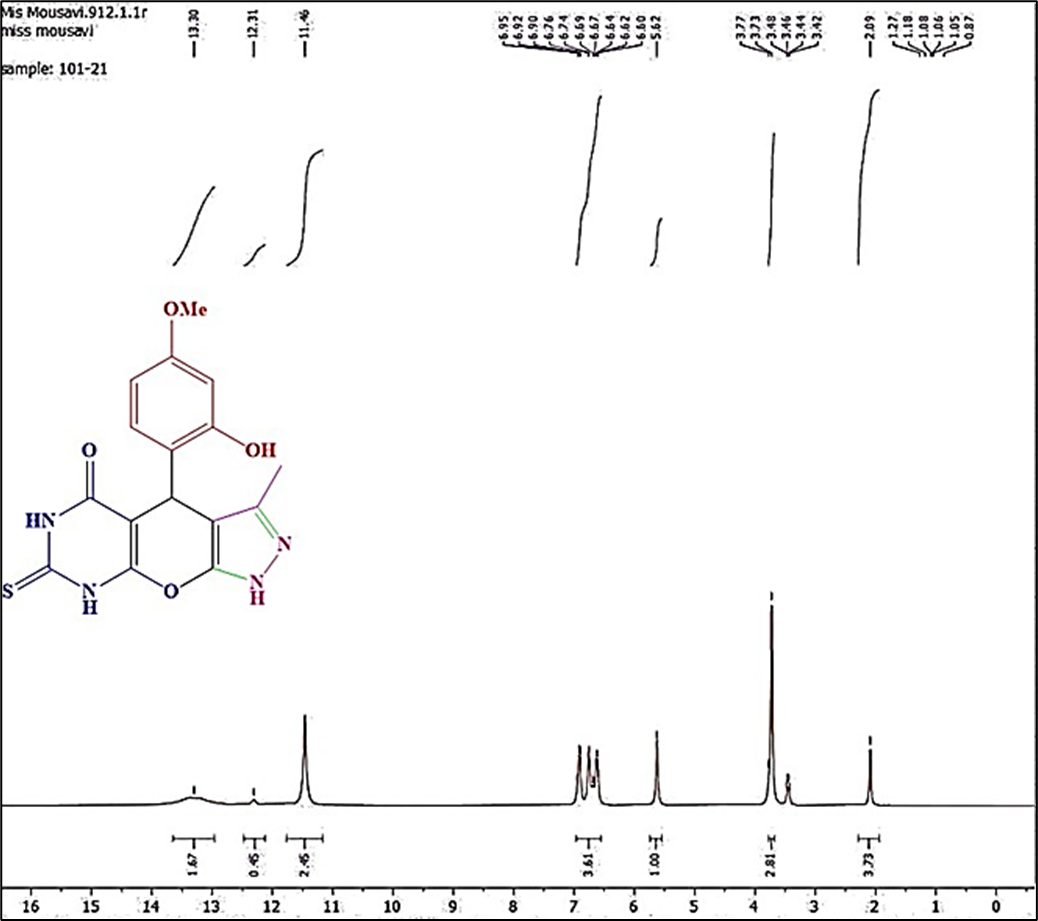


**FIGURE S70.** ^1^H NMR spectrum of Compound **7n.**


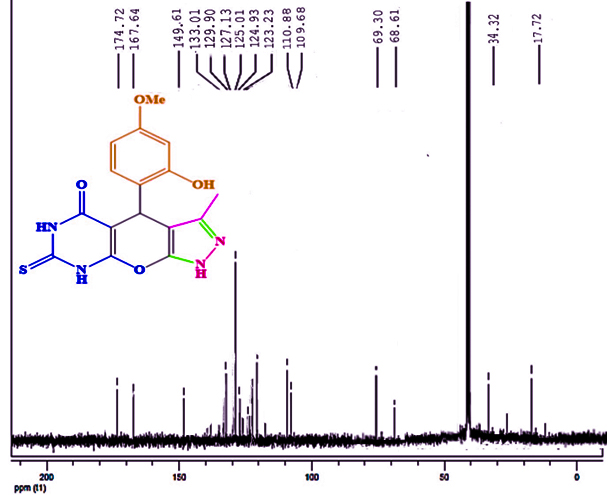


**FIGURE S71**. ^13^C NMR spectrum of Compound **7n**.


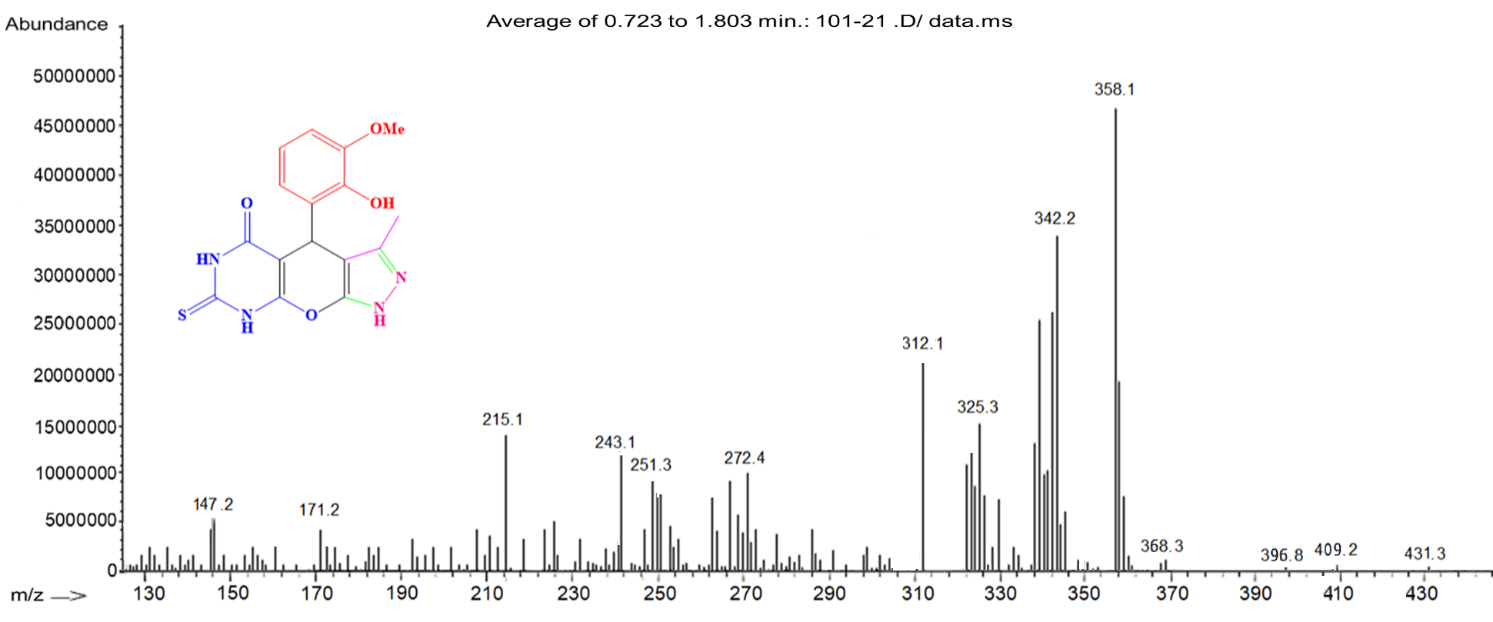


**FIGURE S72**. Mass spectrum of Compound **7n**.

References

Mohammadi Ziarani, G., Khademi, M., Mohajer, F., Anafcheh, M., Badiei, A., & Ghasemi, J. B. (2022). Solvent-free one-pot synthesis of 4-aryl-3, 5-dimethyl-1, 4, 7, 8-tetrahydrodipyrazolo [3, 4-b: 4′, 3′-e] pyridines using Fe3O4@ SiO2@(BuSO3H) 3 catalytic Fe3+ system as selective colorimetric. Res. Chem. Intermed. 48(5), 2111-2133.‏

Omidi, M., & Mobinikhaledi, A. (2022). Sulfonic acid pyridinium chloride-functionalized nanoparticles (MnCoFe2O4@ Niacin-SO3H)+ Cl− as a novel and reusable catalyst for synthesis of tetrahydrodipyrazolopyridines and pyranopyrazoles. Res. Chem. Intermed. 48(10), 4347-4371.‏

Shahbazi-Alavi, H., Safaei-Ghomi, J., Eshteghal, F., Zahedi, S., Nazemzadeh, S. H., Alemi-Tameh, F., ... & Lashkari, M. R. (2016). Nano-CuCr2O4: an efficient catalyst for a one-pot synthesis of tetrahydrodipyrazolopyridine, J. Chem. Res. 40(6), 361-363.‏

Salehi, N., & Mirjalili, B. B. F. (2018). Nano-ovalbumin: a green biocatalyst for biomimetic synthesis of tetrahydrodipyrazolo pyridines in water. Res. Chem. Intermed. 44, 7065-7077.‏

Safaei-Ghomi, J., Shahbazi-Alavi, H., Sadeghzadeh, R., & Ziarati, A. (2016). Synthesis of pyrazolopyridines catalyzed by nano-CdZr 4 (PO4) 6 as a reusable catalyst. Res. Chem. Intermed. 42, 8143-8156.‏

Nagasundaram, N., Peroli, U., Venkatesh, R., Vinoth, N., & Lalitha, A. (2023). Eosin Y as a direct HAT photocatalyst for the synthesis of tetrahydrodipyrazolopyridines under white LED irradiation. Tetrahedron Lett. 117, 154366.‏

Vanegas, S., Rodríguez, D., & Ochoa‐Puentes, C. (2019). An Efficient and Eco‐Friendly One‐Pot Synthesis of Pyrazolopyridines Mediated by Choline Chloride/Urea Eutectic Mixture. ChemistrySelect, 4(11), 3131-3134

Tamaddon, F., & Arab, D. (2019). Urease covalently immobilized on cotton-derived nanocellulose-dialdehyde for urea detection and urea-based multicomponent synthesis of tetrahydro-pyrazolopyridines in water. RSC Adv, 9(71), 41893-41902.‏

Azizi, S., Shadjou, N., & Hasanzadeh, M. (2019). KCC-1-NH2-DPA: an efficient heterogeneous recyclable nanocomposite for the catalytic synthesis of tetrahydrodipyrazolopyridines as a well-known organic scaffold in various bioactive derivatives. Nanocomposites, 5(4), 124-132.‏

Maleki, A., Jafari, A. A., & Yousefi, S. (2017). Green cellulose-based nanocomposite catalyst: design and facile performance in aqueous synthesis of pyranopyrimidines and pyrazolopyranopyrimidines. Carbohydr. Polym. 175, 409-416.‏

Lotfian, N., Heravi, M. M., Mirzaei, M., & Daraie, M. (2020). Investigation of the uncommon basic properties of [Ln (W5O18) 2] 9–(Ln= La, Ce, Nd, Gd, Tb) by changing central lanthanoids in the syntheses of pyrazolopyranopyrimidines, J. Mol. Struct. 1199, 126953.‏

Rana, S., Maddila, S., Yalagala, K., Maddila, S., & Jonnalagadda, S. B. (2015). Covalent Modification of Organo‐Functionalized Graphene Oxide and its Scope as Catalyst for One‐Pot Pyrazolo‐Pyranopyrimidine Derivatives. Open Chem. J. 4(6), 703-707.‏

Ziarani, G. M., Aleali, F., Lashgari, N., Badiei, A., & Soorki, A. A. (2018). Efficient synthesis and antimicrobial evaluation of pyrazolopyranopyrimidines in the presence of SBA-Pr-SO3H as a nanoporous acid catalyst. Iran J. Pharm. Res. 17(2), 525.‏

Yekke-Ghasemi, Z., Heravi, M. M., Malmir, M., Jahani, G., Bisafar, M. B., & Mirzaei, M. (2022). Fabrication of heterogeneous-based lacunary polyoxometalates as efficient catalysts for the multicomponent and clean synthesis of pyrazolopyranopyrimidines. Inorg. Chem. Commun. 140, 109456.‏

Karrabi, M., Malmir, M., Heravi, M. M., & Hosseinnejad, T. (2023). A theoretical and experimental study on ecofriendly-one-pot synthesis of pyrazolopyranopyrimidines catalysed by CuO functionalized montmorillonite.  Inorg. Chem. Commun. 149, 110367.‏

Sadjadi, S., Heravi, M. M., & Daraie, M. (2017). Heteropolyacid supported on amine-functionalized halloysite nano clay as an efficient catalyst for the synthesis of pyrazolopyranopyrimidines via four-component domino reaction. Res. Chem. Intermed. 43, 2201-2214.‏

Tipale, M. R., Khillare, L. D., Deshmukh, A. R., & Bhosle, M. R. (2018). An efficient four component domino synthesis of pyrazolopyranopyrimidines using recyclable choline chloride: urea deep eutectic solvent, J. Heterocycl. Chem. 55(3), 716-728.‏

Amini, H., Neamani, S., & Moradi, L. (2021). Green synthesis of pyrazolo pyrano pyrimidine derivatives using ZnFe2O4/GA as a new effective catalyst in water media. Chemistry, ChemistrySelect, 6 (2), 9608-9615.
