# Supplementary material for: Melamine phosphate-modified magnetic chitosan: a novel biocompatible catalyst for the synthesis of biological tetrahydrodipyrazolopyridine and pyrazolopyranopyrimidine derivatives
Source: Front Chem. 2024 May 15;12:1395008. doi: 10.3389/fchem.2024.1395008 (PMC11134575; doi:10.3389/fchem.2024.1395008)

# Melamine phosphate-modified magnetic chitosan: A novel biocompatible catalyst for the synthesis of biological tetrahydrodipyrazolopyridine and pyrazolopyranopyrimidine derivatives

**Maryam Mousavi-Ebadi[1], Javad Safaei-Ghomi[1]***

*[1]Department of Organic Chemistry, Faculty of Chemistry, University of Kashan, Kashan, 51167, I. R. Iran*

**Corresponding author, Tel: +98 31 55912385; E-mail: safaei@kashanu.ac.ir*

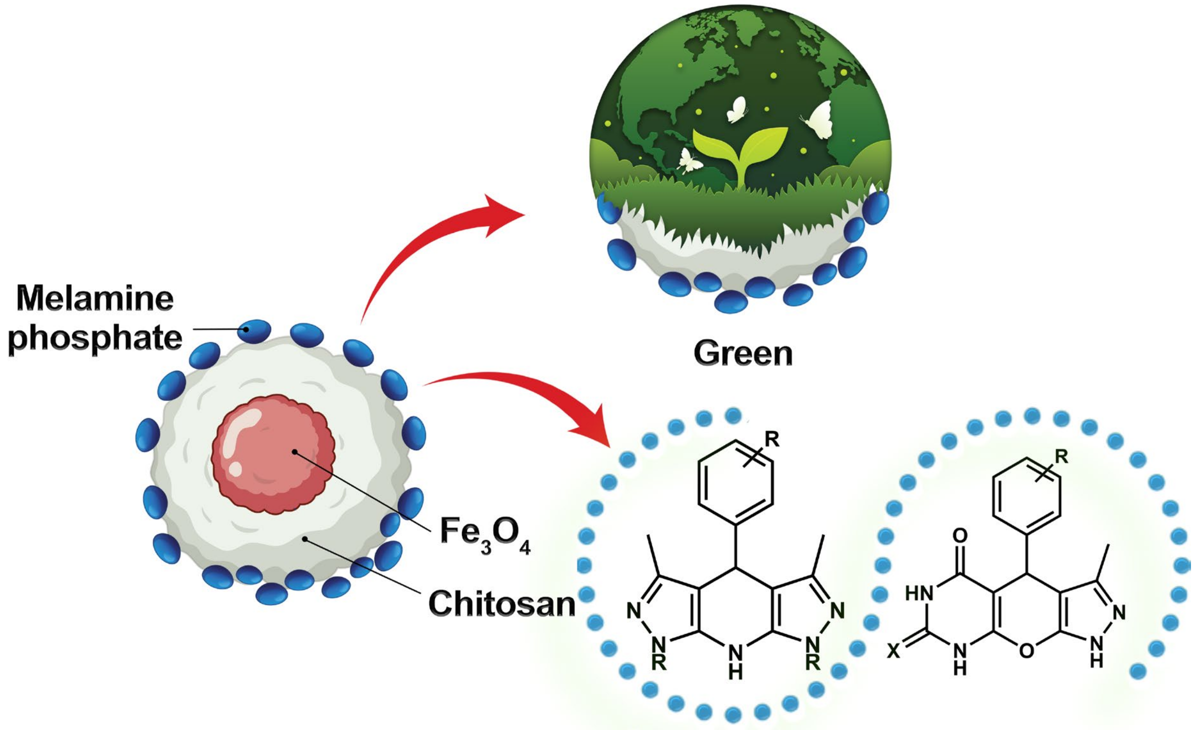

Supplement: Supplementary file 2 [file Image1.pdf]
